# Brazilian recommendations for the management of tuberculosis infection in immune-mediated inflammatory diseases

**DOI:** 10.1186/s42358-025-00449-4

**Published:** 2025-03-20

**Authors:** Viviane Angelina de Souza, Ana Luiza Mendes Amorim Caparroz, Virginia Fernandes Moça Trevisani, Anna Carolina Faria Moreira Gomes Tavares, Ana Karla Guedes de Melo, Anete Trajman, Ana Cristina de Medeiros-Ribeiro, Marcelo de Medeiros Pinheiro, Ricardo Machado Xavier, Odirlei Andre Monticielo, Maria Fernanda Brandão de Resende Guimarães, Flavio Sztajnbok, Sidney Bombarda, Liliana Andrade Chebli, Adriana Maria Kakehasi, Ana Luiza Bierrenbach, Ana Paula Monteiro Gomides Reis, Blanca Elena Rios Gomes Bica, Claudia Diniz Lopes Marques, Cristina Flores, Denise Silva Rodrigues, Eduardo dos Santos Paiva, Eliana Dias Matos, Fernanda Dockhorn Costa Johansen, Helio Arthur Bacha, Joana Starling de Carvalho, José Roberto Provenza, Ketty Lysie Libardi Lira Machado, Licia Maria Henrique da Mota, Lilian David de Azevedo Valadares, Marco Antônio Araújo da Rocha Loures, Margareth Maria Pretti Dalcolmo, Maria Cecilia de Carvalho Bortoletto, Max Igor Banks Ferreira Lopes, Rejane Maria Rodrigues de Abreu Vieira, Ricardo Romiti, Rogerio Saad-Hossne, Rozana Mesquita Ciconelli, Valderilio Feijó Azevedo, Valéria Maria Augusto, Vitor Alves Cruz, Gecilmara Cristina Salviato Pileggi

**Affiliations:** 1 Hospital Universitário da Universidade Federal de Juiz de Fora, Juiz de Fora, Brazil; 2 Faculdade de Medicina de São José do Rio Preto, São José do Rio Preto, Brazil; 3 Universidade de Santo Amaro UNISA, Santo Amaro, Brazil; 4 Universidade Federal de São Paulo (UNIFESP/ EPM), São Paulo, Brazil; 5 Hospital das Clínicas -Universidade Federal de Minas Gerais, Belo Horizonte, Brazil; 6 Hospital Universitário Lauro Wanderley - Universidade Federal da Paraíba, João Pessoa, Brazil; 7 Universidade Federal do Rio de Janeiro (UFRJ), Rio de Janeiro, Brazil; 8 Hospital das Clínicas da Faculdade de Medicina da Universidade de São Paulo, São Paulo, Brazil; 9 Hospital de Clínicas de Porto Alegre - Universidade Federal do Rio Grande do Sul, Porto Alegre, Brazil; 10 Secretaria de Estado da Saúde de São Paulo, São Paulo, Brazil; 11 Faculdade de Medicina da Universidade Federal de Minas Gerais, Belo Horizonte, Brazil; 12 Instituto de Ensino e Pesquisa - Hospital Sírio-Libanês, São Paulo, Brazil; 13 Centro Universitário de Brasília, Brasília, Brazil; 14 Hospital das Clinicas - Universidade Federal de Pernambuco, Recife, Brazil; 15 Centro de Tratamento de Doenças Inflamatórias Intestinais e Imunomediadas, Porto Alegre, Brazil; 16 Instituto Clemente Ferreira - São Paulo, São Paulo, Brazil; 17 Hospital de Clinicas da Universidade Federal do Paraná, Curitiba, Brazil; 18 Hospital Especializado Octávio Mangabeira (Bahia), Salvador, Brazil; 19 Coordenação-Geral de Vigilância da Tuberculose, Micoses Endêmicas e Micobactérias não Tuberculosas, (Ministério da Saúde), Brasília, Brazil; 20 Hospital Israelita Albert Einstein, São Paulo, Brazil; 21 Hospital Universitário da PUC Campinas, Campinas, Brazil; 22 Hospital Universitário Cassiano Antonio Moraes da Universidade Federal do Espírito Santo (UFES), Vitória, Brazil; 23 Hospital Universitário de Brasília, Universidade de Brasília, Brasília, Brazil; 24 Hospital Getulio Vargas de Pernambuco, Recife, Brazil; 25 Universidade Estadual de Maringá/UEM, Maringá, Brazil; 26 Centro de Referência Professor Hélio Fraga – Fiocruz, Rio de Janeiro, Brazil; 27 Hospital Servidor Municipal São Paulo, São Paulo, Brazil; 28 Universidade de Fortaleza (UNIFOR), Fortaleza, Brazil; 29 Universidade Estadual do Ceará (UECE), Fortaleza, Brazil; 30 Faculdade de Medicina de Botucatu - Universidade Estadual Paulista – UNESP, São Paulo, Brazil; 31 Hospital Beneficência Portuguesa de São Paulo, São Paulo, Brazil; 32 Universidade Federal de Goiás (UFG), Goiás, Brazil; 33 Instituto D’Or de Pesquisa e Ensino-UNISA, São Paulo, Brazil

**Keywords:** Tuberculosis infection, Immune-mediated inflammatory disease, Systematic review, Immunosuppressive treatment

## Abstract

**Background:**

The risk of tuberculosis infection (TBI) and its progression to tuberculosis disease (TBD) among persons with immune-mediated inflammatory diseases (IMID) results from a complex interplay of patient and disease characteristics, immunosuppression level, and the epidemiological context. Brazilian recommendations are unclear about TBI screening and its preventive treatment (TPT) in persons with IMID.

**Objective:**

To provide a comprehensive and evidence-based guideline for managing TBI in persons with IMID in Brazil.

**Methods:**

This task force was constituded by 42 specialists with interest in IMID and TBD. A core leadership team (CLT) drafted fourteen clinical questions on the risk of tuberculosis and indications of TPT among persons with IMID who started, or are about to start immunosuppressive drugs. The CLT supervised the systematic reviews and formulated the recommendations. The experts voted using the Delphi Method.

**Results:**

Nine recommendations were established. More than 80% of panelists voted “agree” and “strongly agree” with all statements. In brief, all persons with IMID starting or about to start immunosuppressive treatment should undergo tuberculin skin testing (TST) or interferon-gamma release assays (IGRAs), a chest imaging test and investigation of contact with active pulmonary or laryngeal TBD. TPT is mandatory for those with any positive result after excluding TBD. Exceptions include individuals with a history of TBD or a past positive TBI infection test. IGRA is preferred only in persons BCG-vaccinated in the past 2 years. Those with inconclusive IGRA results can have the test repeated once, and TPT should be offered if it remains indeterminate. TST or IGRA should be repeated yearly, for three years, when the previous test was negative, when starting or changing to a different class of immunosuppressive drug. Overall, the included studies had a low quality of evidence and high risk of bias.

**Conclusions:**

These guidelines are meant to improve the management of TBI in IMID. Health professionals must consider the epidemiological risk, host features, the social scenario, the characteristics of the disease, the access to health resources, and the development of an individualized plan for every patient.

**Supplementary Information:**

The online version contains supplementary material available at 10.1186/s42358-025-00449-4.

## Background

Tuberculosis disease (TBD), caused by *Mycobacterium tuberculosis* (Mtb), in the last four years, was the second cause of death from a single infectious agent, after SARS-CoV-2 [1, 2]. The reported global number of people newly diagnosed with TBD was 7.5 million in 2022. This is the highest number since the World Health Organization (WHO) began global TBD monitoring in 1995. The number in 2022 probably includes a sizeable backlog of people who developed TBD in previous years, but whose diagnosis and treatment were delayed by COVID-related disruptions that affected access to and provision of health services [2]. In 2022, the total number of deaths caused by TBD (including those among people living with HIV) was 1.3 million [2], which will probably put TBD as the leading cause of death again, once SARS-CoV-2 pandemic has been controlled.

Latin America represents around 3% of the global TBD burden, with an estimated 268,000 new cases, 33% of which are located in Brazil. Therefore, the WHO considers the country a priority for worldwide disease control. In 2020, Brazil registered 66,819 new cases of TBD, with an incidence rate of 31.6 cases per 100,000 inhabitants. In 2019, around 4,500 deaths from the disease were reported, with a mortality rate of 2.2 deaths per 100,000 inhabitants [3].

Tuberculosis infection (TBI), the new term used for latent tuberculosis infection, is defined by the presence of viable Mtb and an associated host response without macroscopic pathology (with no disease); the individual has no symptoms or signs consistent with TBD, and this is a non-infectious condition [4].

It is estimated that 1.7 billion individuals have TBI, representing almost a quarter of the world population [5]. Management of TBI is critical because this condition can progress to TBD in 5–10% of individuals when the immune response to Mtb is ineffective. A higher risk of progression from TBI to TBD has been reported in recent contacts, especially in the last five years, people living with HIV, treatment with biologic therapy, diabetes, and malnutrition [6].

According to the Brazilian Guidelines, TBI screening should be accessed in individuals at increased risk of progression to TBD, including people using tumor necrosis factor (TNF) inhibitors or corticosteroids (equivalent to ≥ 15 mg/day of prednisone for more than one month) [1]. They should undergo TST or IGRA tests, imaging tests (chest X-ray or CT), and evaluation of the history of exposure to pulmonary or laryngeal TBD. If either of these tests is positive, or the history of exposure is present, tuberculosis preventive treatment (TPT) should be started, according to national guidelines. In Brazil, isoniazid, rifampicin, or a combination of isoniazid and rifapentine are available for TPT [1, 7].

Until the end of the 90s, the therapeutic arsenal of immune-mediated inflammatory diseases (IMID), including rheumatoid arthritis (RA), psoriatic arthritis (PsA), spondyloarthritis (SpA), psoriasis (PsO), and inflammatory bowel disease (IBD), was based on corticosteroid therapy, immunosuppressants, and conventional synthetic disease-modifying antirheumatic drugs (csDMARDs) [8]. Over the last twenty years, the therapeutic scenario for IMID has changed with the introduction of the biological DMARDs (bDMARDs), a class of medications that inhibit different stages of the immune response or blockade cytokines. These agents altered the natural course of these diseases, preventing (or delaying) damage and complications [8]. In addition to bDMARDs, the so-called signaling pathway inhibitors, or target-synthetic DMARDs (tsDMARDs), have also demonstrated efficacy in controlling IMID [8]. (Table 1).

**Table 1:** Medications available in Brazil for the treatment of IMID

Class	Drugs
▪ Immunosuppressants	Azathioprine, calcineurin inhibitors, mofetil mycophenolate, cyclophosphamide
▪ csDMARDs	Methotrexate, leflunomide, hydroxychloroquine, sulfasalazine
▪ bDMARDs TNF inhibitors	Infliximab, etanercept, adalimumab, golimumab, certolizumab pegol
▪ bDMARDs non-TNF inhibitors	Rituximab (anti-CD20), belimumab (anti-BlyS), anifrolumab (anti-IFN1), abatacept (anti-CD28), tocilizumab (anti-IL6r), secukinumab (anti-IL17), ixekizumab (anti-IL17), guselkumab (anti-IL23), risankizumab (anti-IL23), ustekinumab (anti-IL12/23), vedolizumab (anti-integrins)
▪ tsDMARDs	Tofacitinib, baricitinib, upadacitinib

Source: adapted from Gasparin AA, et al., 2023 [9].

Overall, the presence of IMID itself is already associated with a higher risk of TBD, ranging from 2.0 to 8.9 in RA patients not treated with biologic agents, and a lower risk is observed in PsA or SpA, which may be explained by more significant immunosuppression in RA patients [8]. Other risk factors related to the host are age, recent TBD (less than two years), smoking, alcoholism, and the presence of comorbidities, such as diabetes, and chronic kidney diseases [10].

TNF plays a fundamental role in maintaining granuloma integrity, and blocking TNF is associated with increased susceptibility to TBD in individuals with TBI. Keane et al. reported, in 2001, an increased occurrence of TBD in patients under infliximab treatment. The authors observed that in the USA, the incidence of TBD was 6.2/per 100,000 inhabitants before the introduction of TNF inhibitors (TNFi), and afterward, the incidence increased to 24.4/per 100,000 inhabitants. On this occasion, the authors suggested that patients diagnosed with TBI and requiring TNFi therapy should receive TPT [11].

On the other hand, inhibition of CD20, CD28, IL-1, IL-6, IL-12, IL-23, and IL-17a causes negligible or no effects on TB granuloma [12]. In particular, 19 studies reported the results of trials using tocilizumab, where no cases of TBD were observed, and the protocol did not include TBI screening as an inclusion criterion [12]. Similar results were observed in trials using rituximab (RTX), abatacept (ABA), ustekinumab (UTK), secukinumab (SEC), ixekizumab (IXK), risankizumab (RIZ), and guselkumab (GUS), with no or low occurrence of TBD cases [12, 13]. Recent studies available in the literature suggest that IL-17 or IL-23 inhibitors are highly safe in patients with TBI or at risk of TBD, especially when compared to TNFi, even suggesting that TPT should be dispensed before starting treatment with these bDMARDs, in patients with TBI and at risk of adverse events to treatment [14]. More data are needed for tsDMARDs [12, 13].

In persons with IMID, according to the Brazilian Guidelines, screening and treating TBI is indicated in those who will use chronic corticosteroid therapy and TNFi. This indication also applies to non-TNF bDMARDs and tsDMARDs [1]. As mentioned earlier, there is evidence suggesting that the risk of developing TBD varies among the different classes of DMARDs available. Therefore, it is important to assess which patients require this evaluation and treatment. The risk of TBI is multifactorial, depending on patient and disease characteristics, immunosuppression level, and the epidemiological context. Currently, there are still unresolved controversies and unmet needs in the management of TBI in individuals with IMID. This highlights the necessity for national recommendations that can provide guidance to specialists prescribing DMARDs for clinical management of IMID.

## Methods

### Task force

The Brazilian Society of Rheumatology conducted this project under the coordination of the Committee of Endemic and Infectious Diseases and in collaboration with the Brazilian Societies of Dermatology, Pneumology, Infectious diseases specialists and the Study Group of Inflammatory Bowel Disease in Brazil (GEDIIB). This task force consisted of 27 rheumatologists, 3 infectious diseases specialists, 4 pulmonologists, 3 gastroenterologists, 2 dermatologists, 1 internist, 1 epidemiologist, and 1 member of the division of Surveillance for Tuberculosis, Endemic Mycoses and Non-Tuberculous Mycobacteria, from the Ministry of Health.

These members were divided into 3 groups: a core leadership team (CLT), a literature review team (LRT), and a voting panel (VP).

The CLT was responsible for drafting the clinical questions, supervising the systematic review and data extraction, drawing up the recommendations based on the literature review results, organizing them, and sending them to the voting panel. They also summarized the voting results and drafted this publication and its supplementary material.

The LRT was composed of professionals with experience in systematic reviews and was responsible for the stages of literature search, selecting and including studies, extracting data, analyzing the results, and drawing up the recommendations.

The VP comprised all the experts involved in the task force who evaluated the proposed recommendations and voted according to their experience.

The table with the full name and proficiency of each member of the CLT, the LRT and the VP is available in Appendix 1 of the Supplementary Material.

### Establishing key principles and clinical questions development

Initially, the CLT established the following key topics that should guide the literature review: the establishment of a routine for evaluating TBI in patients with IMID, the indications for when to start TPT in these patients, the clinical, laboratory, and radiological parameters for diagnosing TBI in patients with IMID, evaluation of the efficacy, limitations, and interpretation of the results of the TST (tuberculin skin test) and IGRA (interferon-gamma release assay) tests in patients with IMID, the possible interference of the BCG vaccine in these results, and also, in which clinical situations there would be a need to repeat these tests.

Based on these key topics, 14 clinical questions (CQ) were drawn up, which served as the basis for choosing the terms that shaped the literature search strategies. These clinical questions were as follows:

- CQ 1: What is the difference in TBD risk in IMID patients being treated with the different classes of DMARDs?
- CQ 2: In patients with previous TBI, what is the risk of developing TBD after starting treatment with DMARDs?
- CQ 3: What is the risk of a new episode of TBD for IMID patients with a history of treated TBD before starting immunosuppression?
- CQ 4: What is the sensitivity, specificity, negative and positive predictive values of TST compared to IGRA in screening for TBI before initiation, and during immunosuppressive treatment in IMID patients?
- CQ 5: Does performing both IGRA and TST tests increase the sensitivity of diagnosing TBI? Should the diagnosis of TBI be made based on only one or both positive tests? When one of the tests is negative, should the other test be performed? What is the best course of action in case of conflicting results between these tests?
- CQ 6: What is the sensitivity, specificity, positive and negative predictive values of the available IGRA tests: Quantiferon gold in tube, Quantiferon gold plus and Elispot TB?
- CQ 7: What is the clinical significance of indeterminate or inconclusive IGRA, and how to proceed based on this result?
- CQ 8: In TBD endemic areas, should the TST cutoff point in persons with IMID using DMARD be reduced to less than 5 mm for diagnosing TBI?
- CQ 9: In IMID, if pre-treatment TBI screening with TST or IGRA is negative, how often should we repeat this screening throughout immunosuppressive treatment?
- CQ 10: How should we manage persons with IMID using or about to start the use of DMARD with a history of previous TBD or a positive IGRA/TST?
- CQ 11: In IMID patients with previous negative TBI screening, if a change in immunosuppressive treatment is needed, should TBI screening be repeated?
- CQ 12: In IMID patients exposed to TBD in the past who will start immunosuppressive treatment, should TPT treatment be offered, regardless of the results of the TST or IGRA tests? When TBI tests are not accessible, should we recommend TBI treatment in IMID patients re-exposed to TBD who will begin immunosuppressive treatment?
- CQ 13: Is TBI treatment indicated for IMID patients previously exposed to TBD and who will use biological or immunosuppressive treatment, considering contact and exposure regardless of TST or IGRA result, or if it is impossible to carry out this research?
- CQ 14: What is the impact of the BCG vaccine on the TST and IGRA results? After which interval should we consider that this immunization no longer influences the results of these tests?

### Literature search

To identify relevant evidence for the 14 CQ, the literature search strategies were devised by the LRT, considering the population of interest for this study, the focus of each clinical question (intervention, treatment or complementary investigation), the comparators, and the most relevant outcomes.

The searches were carried out on PubMed/MEDLINE, Cochrane Library, including Cochrane Central Register of Controlled Trials (CENTRAL), Latin America and Caribbean Health Sciences Literature (LILACS) and Embase, and Google Scholar, as well as manual search of the reference list of included studies and previously published reviews on similar topics.

The first literature search was carried out in September 2019 and updated annually until September 2023, to include possible new studies.

The supplementary material (Appendix 1) provides details on the search strategy for each CQ.

### Eligibility criteria and study selection

The eligibility criteria varied according to the CQ, involving patients with or without a history of TBD or TBI. The questions related to risk factors involved exposure to corticosteroids, immunosusuppressive drugs, synthetic, biological or tsDMARDs, comparing exposed and unexposed populations. The questions related to diagnostic tests addressed the accuracy of the TST and/or IGRA. For each CQ, the relevant outcomes of interest concerning the management of TBI in IMID were selected.

We included cross sectional, cohort and case-control, randomized or non-randomized clinical trials.

Studies in languages other than English, Portuguese or Spanish were excluded, as were studies with insufficient data for analysis, even after contacting the main authors.

Eligible reports underwent full-text screening by two independent reviewers. Disagreements were resolved through discussion or, if required, by consulting a third author.

The selection and inclusion stages of the studies were reported following the recommendations of the Preferred Reporting Items for Systematic reviews and Meta-Analyses (PRISMA) statement, and are detailed in the supplementary material (Appendix 1).

### Data analysis

The ROB-2 tool was used to assess the risk of bias in randomized clinical trials and the ROBINS-I tool was used to assess the risk of bias in observational studies [15, 16]. The LRT assessed the overall quality of the evidence for each CQ outcome based on the risk of bias, degree of imprecision, inconsistency in reported results among studies, indirectness, and possible publication bias according to the GRADE method [17, 18]. The GRADE specifies four categories in which the quality of evidence may be rated: high, moderate, low, and very low [17, 18], as presented in Table 2.

**Table 2:** Grading of the certainty of evidence for recommendations

Quality rating	Interpretation
High	Very confident that the true effect lies close to that of the estimate of the effect
Moderate	Moderately confident in the effect estimate; the true effect is likely to be close to the estimate of the effect but there is a possibility that it is substantially different
Low	Our confidence in the effect estimate is limited; the true effect may be substantially different from the estimate of the effect
Very low	Very little confidence in the effect estimate; the true effect is likely to be substantially different from the estimate of the effect

The CLT reviewed the evidence report and addressed possible evidence gaps prior to presentation to the VP. For each of the recommendations presented in this guideline, we will provide the certainty of the evidence, assessed using the GRADE method [17, 18].

### Consensus building

After analyzing the evidence presented in the 14 CQ, the CLT drew up 9 statements with recommendations on the management of TBI in IMID patients being treated with the various drugs available.

Following the Delphi Method, these statements were sent by email to each member of the VP via a Google^®^ Form for individual and anonymous voting [19].

For each of the 9 statements, members should indicate their level of agreement: (1) Strongly disagree; (2) Disagree, (3) Neither agree nor disagree (neutral); (4) Agree; and (5) Strongly agree [20].

To support their decisions, all members of the VP also received an evidence report that summarized the entire process of collecting and analyzing the evidence obtained in the literature review.

After the voting process, the CLT reviewed the statements and drafted the manuscript with the relevant recommendations. The drafted manuscript was sent to all task force members for approval before submission.

This report followed the AGREE guidelines [21].

## Results

### Statements and recommendations

Overall, the studies included were retrospective, had a non-comparative longitudinal design, with a high risk of bias and heterogeneity, and included persons from different geographic areas and epidemiological risks for TBD. In this way, all the recommendations provided here are conditional and health professionals must consider the epidemiological risk, individual factors, the social context, the characteristics of the disease, access to health resources, and the patient’s preferences to choose the ideal course of action for each patient at a given point in their treatment.

The recommendations generated in this document were established through consensus among the panelists, in which more than 80% of panelists voted for options 4—agree—and 5—strongly agree in all scenarios.

Table 3 summarizes the nine recommendations and corresponding CQs, whose search strategies provided the evidence used to develop each recommendation, the overall quality of evidence across all critical outcomes (assessed using GRADE), and the level of agreement (LOA) between panelists.

**Table 3:** Summary of recommendations

Recommendations	Clinical question	Certainty of evidence (GRADE)*	Level of agreement**
1. Routine investigation for TBI, and consequent TPT, when indicated, should be carried out in all persons with IMID who will undergo immunosuppressive treatment, regardless of the class of drug chosen, if there is no recent history of treatment for TBD or TBI.	CQ1, CQ2, CQ3	Very low	95.0%
2. TBI diagnosis should be considered and TPT indicated in the following situations: 1. TST ≥ 5 mm; 2. A positive IGRA; 3. Signs of lung TBD sequelae in imaging tests (chest X-ray or CT) in the patient not previously treated for TBD; 4. Recent exposure to pulmonary or laryngeal TBD, if there is no clinical, and/or imaging evidence of TBD.	CQ8, CQ10, CQ12, CQ13	Moderate	100.0%
3. In case TST/IGRA are not available: -Persons with previous history of TBI/TBD treatment: once TBD is excluded, TPT is not mandatory, even in the absence of a TBI test. -Persons with no history of TBI/TBD treatment: once TBD is excluded, TPT should be recommended in a shared decision with the patient, regardless of the class of medication to be used.	CQ2, CQ3, CQ12, CQ13	Low	95.0%
4. Both TST and IGRA can be used to diagnose TBI in IMID persons, since there is no gold standard test for diagnosing TBI in clinical practice.	CQ4	Moderate	100.0%
5. When screening for TBI in persons with IMID, it is neither mandatory nor recommended to perform TST and IGRA tests simultaneously, and immunosuppressive treatment should not be postponed in order to perform both tests. If the first test is negative, the other can be considered. TPT should be started at any time if one of the tests is positive.	CQ4	Moderate	97.5%
6. In the event of an indeterminate IGRA test result, it is recommended to repeat the test as soon as possible. If the result remains inconclusive, consider TPT.	CQ4, CQ5, CQ7	Moderate	97.5%
7. If the pre-treatment TST/IGRA test is negative, annual repetition of the test is recommended until the third year of treatment, especially in IMID patients taking TNFi. After this period, clinical and epidemiological surveillance is recommended during the immunosuppressive treatment, regardless of the class. In persons with a previous history of treatment for TBI or TBD, screening should not be repeated.	CQ4, CQ9, CQ10, CQ12	Moderate	85.0%
8. In IMID, If it is necessary to change the medication, regardless of the class, if there is a previous negative TBI screening, TST/IGRA should be performed annually for the next 3 years, according to recommendation 7.	CQ1, CQ2, CQ3, CQ4, CQ9, CQ10, CQ11	Very low	85.0%
9. In persons with IMID vaccinated with BCG in the two years before starting immunosuppressive treatment, IGRA is preferable to TST for TBI screening. If BCG was administered more than two years before the introduction of treatment, a positive TST or IGRA result should be interpreted as a diagnosis of TBI and TPT should be started as soon as TBD is ruled out.	CQ4, CQ6, CQ14	Low	100.0%

* GRADE quality rating

** Sum of the percentage of votes in “Strongly agree” and “Agree” on 5-point Likert scale

Complete references of all included studies as well as their main characteristics are available in the supplementary material (Appendix 2).

Recommendation 1. Routine investigation for TBI, and consequent TPT when indicated, should be carried out in all persons with IMID who will undergo immunosuppressive treatment, regardless of the class of drug chosen, if there is no recent history of treatment for TBD or TBI.

For this analysis, 10,713 reports were initially evaluated, with data from 17,246 patients. After exclusion, 124 studies were analysed.

The incidence rates (and 95% confidence intervals) of TBD in persons with IMID treated with the different classes of DMARDs extracted from the included studies were combined, determining an overall incidence rate of TBD in each of these groups. These pooled incidences are shown in Table 4.

The Incidence Rate Ratio (IRR) was used as the measure of effect to compare the incidence rates of TBD across different DMARD classes. The analysis was conducted using the general inverse variance (IV) method with a fixed-effects model.

**Fig. 1:**
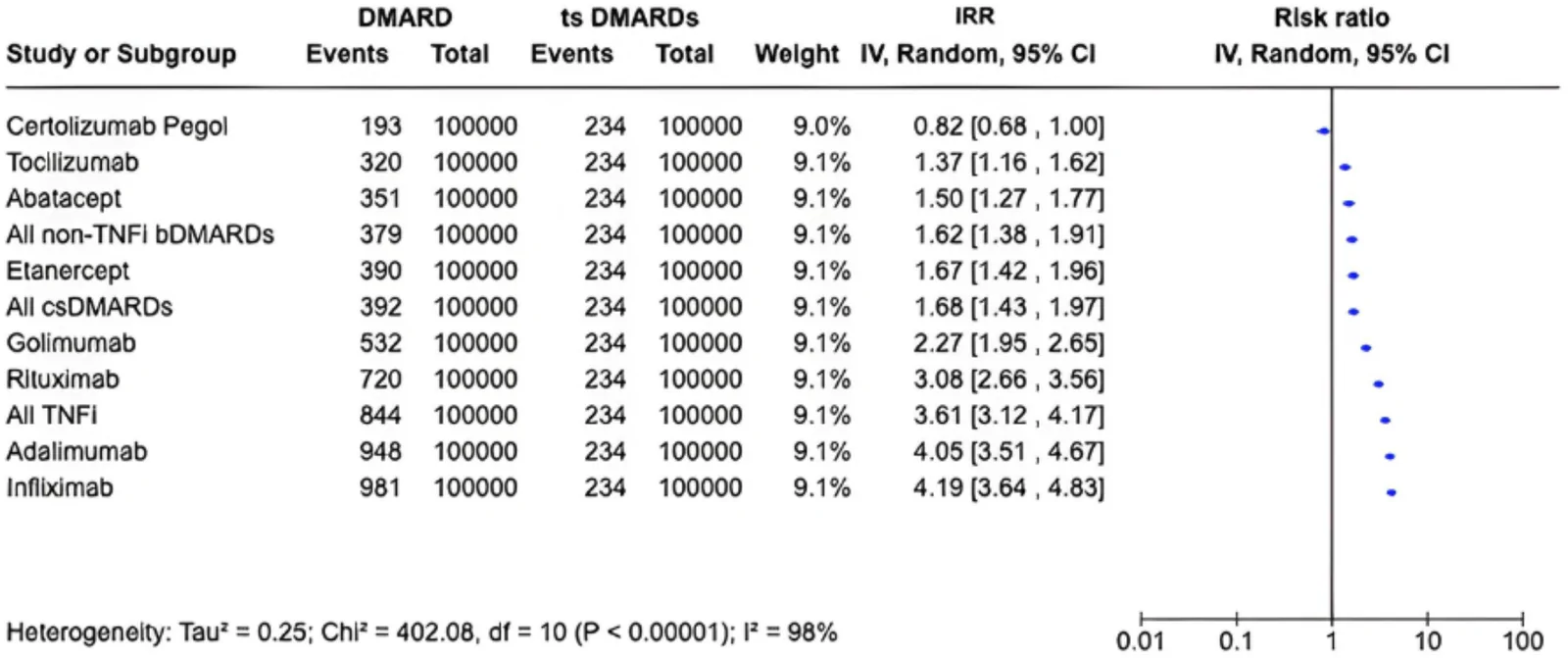
Incidence rate ratio (IRR) of TBD in IMID patients being treated with the different classes of DMARDs

The tsDMARDs were the medications with the lowest incidence rates of TBD in persons with IMID, and were therefore, used as the main comparator concerning the other classes. Table 5 and Fig. 1 show the results of IRR of TBD in persons with IMID being treated with the different classes of DMARDs.

**Table 4:** Pooled incidences of TBD among IMID patients treated with different classes of DMARDs

DMARD	Pooled incidences of TBD/100,000py (% IC 95%)	Test for overall effect
All TNFi Etanercept Adalimumab Infliximab Golimumab Certolizumab	0.84 (0.7–0.97) 0.39 (0.24–0.54) 0.85 (0.62–1.07) 0.98 (0.60–1.36) 0.47 (0.28–0.66) 0.19 (0.02–0.36)	*p* < 0.00001 *p* < 0.00001 *p* < 0.00001 *p* < 0.00001 *p* < 0.00001 *p* = 0.03
All non-TNFi bDMARDs Rituximab Abatacept Tocilizumab	0.35 (0.25–0.46) 0.72 (-0.63-2.07) 0.32 (0.21–0.43) 0.32 (0.01–0.63)	*p* < 0.00001 *p* = 0.3 *p* < 0.00001 *p* < 0.00001
All tsDMARDs	0.18 (0.09–0.27)	*p* < 0.00001
All csDMARDs	0.20 (0.05–0.34)	*p* = 0.009

**Table 5:** Incidence rate ratio (IRR) of TBD in IMID patients being treated with the different classes of DMARDs

DMARD	Incidence/100,000 py	IRR	*p*-value
tsDMARDs	234	Comparator	Comparator
csDMARDs	392	1.6748	< 0.00001
All TNFi	844	3.6056	< 0.00001
Etanercept	390	1.6672	< 0.0001
Adalimumab	948	4.0488	< 0.0001
Infliximab	981	4.1910	< 0.0001
Certolizumab pegol	193	0.8248	0.0474
Golimumab	532	2.2752	< 0.0001
Non-TNFi bDMARDs	379	1.6198	< 0.0001
Tocilizumab	320	1.3690	0.0003
Rituximab	720	3.0783	< 0.0001
Abatacept	351	1.4977	< 0.0001

Although TNFi had an increased risk compared with non-TNFi, all bDMARDs increased the incidence of TBD.

TNFi users had a higher incidence rate of TBD than tsDMARD users, except for certolizumab, whose analyses were not statistically relevant. Adalimumab and infliximab increased the rates of TBD in a very similar way—an incidence approximately 2.5 times higher compared to users of tsDMARD.

Etanercept, adalimumab, infliximab, and golimumab considerably increased the rates of TBD in their users when compared to patients treated with certolizumab.

The other classes of DMARDs, when compared to tsDMARDs, showed varied results: rituximab users had a 1.8 times higher rate of TBD, while the risk among users of csDMARDs, tocilizumab, and abatacept did not seem to differ. Rituximab increased the risk of TBD by 2.2 times compared to patients receiving tocilizumab.

Although the statistical analyses point to a higher incidence of TBD among patients being treated with TNFi, especially infliximab and adalimumab, when compared to the other classes of DMARDs, the interpretation of these results must consider some critical limitations.

Some literature data corroborate these results from our systematic review. A recent study evaluated the occurrence of TBD as the primary outcome, with a follow-up of three years, in patients with RA from the British Society for Rheumatology Biologics Register for Rheumatoid Arthritis (BSRBR-RA), who initiated bDMARD from the first to fifth line of therapy between 2001 and 2019, but tsDMARDS were not included. A total of 33,897 treatment courses were included in this analysis, 10,643 with etanercept, 7,835 with adalimumab, 4,430 with infliximab, 1,614 with certolizumab, 5,556 with rituximab, 2,633 with tocilizumab and 1186 with abatacept, comprising 62,513 person-years of follow-up. There were 49 cases of active TBD during the study, with an incidence rate of 8.5 (95% CI 5.7, 9.9) per 10,000 patient-years. No cases were observed in the tocilizumab group. The crude incidence rates of active TBD were higher with adalimumab and infliximab than etanercept. The unadjusted HR (95% CI), with etanercept as the reference were 3.3 (1.4 to 7,8) for adalimumab, 3.4 (1.6 to 7.3) for infliximab, 1.6 (0,3 to 7.2) for certolizumab, 0.2 (0.0-1.4) for rituximab and 1.2 (0.1–9.4) for abatacept. It is not recorded whether patients in the BSRBR-RA had received TPT prior to starting bDMARDs [22].

The anti-cytokines IL-17, IL-12 and IL-23 bDMARDs are not mentioned in our review because the studies retrieved contained a small number of cases of TBD in persons with IMID treated with these drugs, making comparisons with other DMARDs difficult.

In line with our findings, the Skin Inflammation & Psoriasis International Network–Fondation René Touraine (SPIN-FRT) has published a recommendation for the treatment of psoriasis with bDMARD and tsDMARD in psoriasis patients with TBI or at risk for TBD progression. This task force highlighted that IL-12/23, IL-17, IL-23, and TYK2 inhibitors have different mechanisms of action related to TNFi, and current evidence suggests that some of these agents are arguably not associated with an increased risk of TBD reactivation. They identified, with limited evidence, a low TBD incidence with IL-17 and IL-23 inhibitors in this population and prompted the need for updates to the existing guidelines [23].

The evidence regarding the risk of TBD associated with the use of tsDMARDs in IMID, suggests that this risk may be lower compared to other immunosuppressive therapies, like TNFi, probably due to a more selective mechanism of action, as they inhibit specific cytokine signaling pathways (JAK-STAT pathway), such as IL-6, IL-23, and interferon-gamma, which are involved in the inflammatory response. This low incidence was observed in clinical trials with these medications and also in real-life studies [24].

Various studies have shown an increased risk of TBD with the use of bDMARDs or csDMARDs. This is probably due to T cell dysfunction and depletion, changes in cytokines, complement dysfunction, and metabolic abnormalities [25–27]. A recent Chinese study has evaluated 270 patients with rheumatic diseases which developed TBD from 2009 to 2022. Based on the medication used for rheumatic immune diseases before TBD, the patients were divided into three groups: bDMARDs, which included the use of TNFi, IL-17 and IL-6 inhibitors, abatacept, and B-cell targeted therapies; no immunotherapy group, with patients who did not receive these drugs to treat their rheumatic disease, and the csDMARDs group, that included the use of sulfasalazine, methotrexate, leflunomide, azathioprine, cyclophosphamide, and corticosteroids. There were 31 cases of TBD in the bDMARDs group, 98 cases in the no immunotherapy group, and 141 cases in the csDMARDs group. The bDMARDs group had more extrapulmonary TBD and the csDMARDs group more cavitations [28].

Some important aspects should be considered. Firstly, we highlight the low quality of the evidence gathered through the literature search. Most of the studies selected in this analysis are retrospective, non-comparative longitudinal studies with a high risk of bias, factors which significantly influence the strength of the recommendations resulting from their analysis.

There is a publication bias regarding TNFi compared to the other classes of DMARDs. This can be explained by the fact that many of the studies included were conducted more than a decade ago, when this class of drugs was widely used and was, therefore, the focus of the publications. Thus, we observed a disproportion in the number of studies on TNFi compared to the other medications.

Many of the included studies do not discriminate whether there has been TPT or treatment of active TBD before the analysis of TBD occurrence, thus it is impossible to assess the impact of these critical factors on the incidence rates of this disease.

Another important point is that the search did not retrieve studies that assessed the risk of TBD independently with the various csDMARDs, as well as immunosuppressants, according to the classes of medications listed in Table 1. The studies evaluated these classes in combotherapy with bDMARDs or tsDMARDs, therefore, it was not possible to assess the risk of each of these classes independently.

Finally, the studies were carried out in different geographical regions, with different population incidence rates of TBD and, therefore, different epidemiological risks of developing this infection, regardless of the underlying disease or the use of the different classes of drugs.

These facts altogether result in a high degree of heterogeneity in the analysis and reduce the strength of the recommendation.

We conclude that, despite an extensive database search and the examination of around 10,000 titles, the existing literature does not allow us to differentiate, with a reasonable degree of statistical certainty, the risk of TBD in patients with IMID being treated with the different classes of DMARDs. However, as the risk of developing TBD is higher in patients with IMID taking DMARDs [8], we conditionally recommend that TBI screening should be carried out in all patients taking DMARDs, regardless of class, especially in Brazil and other countries with high TBD incidence rates.

 **LOA: 67.5% Strongly agree; 27.5% Agree. Sum of the percentage of “Strongly agree” and “Agree”: 95%. Overall quality of evidence across all critical outcomes: Very low.**

Recommendation 2. TBI diagnosis should be considered and TPT indicated in any the following situations: TST ≥ 5 mm; a positive IGRA; signs of lung TBD sequelae in imaging tests (chest X-ray or CT) in the patient not previously treated for TBD; recent exposure to pulmonary or laryngeal TBD, if there is no clinical, and/or imaging evidence of TBD.

In persons with IMID, including candidates for TNFi treatment, a combined approach based on immunological tests, clinical history, chest X-ray, or computed tomography (CT) scan casual findings can be helpful for the indication of TPT [1].

There is no gold standard test for TBI diagnosis. The TST/IGRA results, the presence of TBD sequelae in imaging studies (chest X-ray or chest CT scan), and known exposure to an index patient with active pulmonary or laryngeal TBD should be considered for the diagnosis of TBI.

The use of TNFi therapy and other classes of DMARDS increases the risk of TBD, and this risk can be substantially reduced with rigorous TBI screening and TPT [1].

Both TST and IGRA have advantages and limitations. Both tests can be used for TBI evaluation in persons with IMID, as in other indications. TST cut-off for persons using DMARDs should be 5 mm [1]. There is insufficient evidence to recommend reducing the TST cutoff below 5 mm in IMID patients [29–33]. As for IGRA tests, positivity is considered as by the manufacturer’s recommendations.

Chest X-ray is needed before starting DMARDs. Chest X-ray demonstrating any sign of TBD sequelae such as fibrotic sequelae, calcified nodules in the upper lobe, apical pleural thickening, upper lobe bronchiectasis, interstitial granulomatous calcification, cavitation, and lymph node or pericardial or pleural calcification in a person not previously treated for TBD indicates TBI and TPT should be offered, even in the absence of a positive TBI test [1].

CT scans are not routinely recommended in the screening of TBI, if a chest X-ray is available, but casual findings of the same TBD sequelae in CT scans performed for other reasons in persons using DMARDs should prompt TPT prescription, regardless of TBI test results [1].

In cases of confounding images or in the presence of interstitial lung disease, pleural effusion or something that compromises the accuracy of the X-ray, a chest CT scan should be requested, as well as a specialist opinion (pulmonologists/infectious diseases specialists) [1].

History of known recent exposure (less than two years), to persons with pulmonary or laryngeal TBD, defined as present or past household or close community contact with known cases of TBD in the past two years in persons using DMARDs, should be considered as high-risk for TBI and TBD, and TPT should be indicated [1].

Household contact is someone who shares the same enclosed living space with the index case for one or more nights or frequent/extended daytime periods during the three months before the start of the current treatment [29–32].

A close community contact is a person who is not in the household but shares an enclosed space, such as a social gathering place, workplace, school or facility, for extended periods during the day with the index case, during the previous three months [29–32].

In summary, this committee recommends that TPT should be offered in the presence of at least one of the following: positivity of TST or IGRA, signs of TBD sequelae in lung imaging tests or history of recent TBD exposure.

 **LOA: 82.5% Strongly agree; 17.5% Agree. Sum of the percentage of “Strongly agree” and “Agree”: 100%. Overall quality of evidence across all critical outcomes: Very low.**

Recommendation 3. In case TST/IGRA are not available:

-Persons with previous history of TBI/TBD treatment: once TBD is excluded, TPT is not mandatory, even in the absence of a TBI test.
-Persons with no history of TBI/TBD treatment: once active TBD is excluded, TPT should be recommended in a shared decision with the patient, regardless of the class of medication to be used.

In a scenario where tests for diagnosing TBI (TST and/or IGRA) are unavailable, if the patient has already undergone treatment for TBI or TBD previously, there is no indication to treat TBI again. If TBI/TBD has not been previously treated, TPT should be recommended if the tests are unavailable, in a shared decision with the patient since TBD is excluded, regardless of the class of medication to be used. In both situations, it is important to consider chest image exams and patient’s epidemiology.

Although there is evidence of an increased risk of developing TBD in persons treated with bDMARDs or other immunosuppressive drugs with a positive TBI test compared to those with a negative test, there is no evidence of the risk in persons without the test results [6, 33–35].

According to the Brazilian Society of Rheumatology, patients with RA, when using bDMARDs, with a negative history of contact with a case of pulmonary TBD should receive TPT if both tests (TST/IGRA) are unavailable. The decision to treat TBI should be individualized and consider risks and benefits. When assessing the potential benefit of treatment, consider increased epidemiological risk (high disease burden in the living environment) and synergy of risk factors for progression to TBD [36].

Because of the high risk of disease in high transmission settings in immunosuppressed persons, we extrapolate the WHO recommendations for people living with HIV [2] and recommend treating all persons who will be prescribed immunosuppressive drugs, regardless of the class, especially those who have had recent contact (within two years) with a person with active pulmonary or laryngeal TBD if a TBI test is not available, after excluding TBD with symptom screen and a chest image exam, in a shared decision with the patient.

 **LOA: 60% Strongly agree; 35% Agree. Sum of the percentage of “Strongly agree” and “Agree”: 95%. Overall quality of evidence across all critical outcomes: Low.**

Recommendation 4. Both TST and IGRA can be used to diagnose TBI in IMID persons, since there is no gold standard test for diagnosing TBI in clinical practice.

IGRA and TST are recommended as screening methods for TBI, along with epidemiological risk assessment, and radiographic evaluation [37].

The TST includes a mixture of precipitated proteins from mycobacteria culture, being less specific to *Mycobacterium tuberculosis* when compared to IGRA, that is driven for more specific peptides (ESAT-6 and CFP-10), which are not present in the bacillus Calmette–Guérin (BCG) vaccine, neither in most of the other non-tuberculous mycobacteria. However, it is necessary to consider the prevalence of TBI in every country for a more suitable interpretation. Thus, IGRA may be indicated as a feasible option for TBI screening [37].

Both IGRA and TST may present false negative results due to anergy related to immunosuppression, particularly in those with RA, PsA and IBD using DMARDs. Thus, a negative TBI screening test does not entirely rule out the possibility of TBD after DMARD exposure over time [38, 39].

TST may have limited specificity because previous contact with other mycobacteria and BCG vaccination may influence the results. Likewise, immunosuppression, performance or incorrect reading of the test can affect sensitivity. TST performance by an experienced professional is recommended to avoid variability in interpretation. On the other hand, IGRAs also have limitations and its sensitivity may be reduced in immunosuppressed persons and in children. The rate of indeterminate results has been reported to be up to 40%. These findings challenge previous evidence that IGRA may be more suitable than TST in the immunocompromised host, as the immunosuppressant causes a significant reduction in the number of T cells that are essential for the release of adequate levels of IGRA. Furthermore, several studies have suggested very different concordance rates between IGRA and TST, likely due to heterogeneity (e.g., TBD prevalence, varying immunosuppressive therapies, or underlying BCG status) [38, 39].

Comparing TST and IGRA in persons with IMID is complex, considering that there is no gold-standard tool for diagnosing TBI, the test prevalence varies worldwide and the influence of the degree of immunosuppression of these patients that may occur [38, 39].

Therefore, both tests can be used to assess TBI, depending on the availability and specific characteristics of each patient. The healthcare professional must consider the limitations of each test when interpreting the result.

Table 6 summarizes the sensitivity and specificity of the tests in IMID, which resulted from our literature review. The analysis was carried out using either positive test as a gold standard reference. There was no superiority between them.

**Table 6:** The sensitivity, specificity, positive predictive value (PPV), and negative predictive value (NPV) of TST and IGRA having either test positive as the golden standard

	TST	IGRA
Sensitivity (%)	61 (95%CI: 58 a 64)	43 (95%CI: 41 a 46)
Specificity (%)	82 (95%CI: 81 a 83)	90 (95%CI: 89 a 91)
PPV	60	64
NPV	16	21

 **LOA: 80% Strongly agree; 20% Agree. Sum of the percentage of “Strongly agree” and “Agree”: 100%. Overall quality of evidence across all critical outcomes: Moderate**.

Recommendation 5. When screening for TBI in persons with IMID, it is neither mandatory nor recommended to perform TST and IGRA tests simultaneously, and immunosuppressive treatment should not be postponed in order to perform both tests. If the first test is negative, the other can be considered. TPT should be started at any time if one of the tests is positive.

The studies included in this analysis showed a heterogeneous prevalence of TBI (29%, ranging from 6 to 48%). The discordance rate between both classes of tests was high. Without a gold standard test, the analysis of the diagnostic performance of each of the tests, IGRA and TST, was carried out considering the other test as a reference. According to this analysis, using both tests simultaneously increased the chance of diagnosing TBI by 20%, as shown in Table 7.

**Table 7:** Performance analysis regarding TST or IGRA for the diagnosis of TBI, considering as reference the other test, in the absence of gold standard method

	IGRA	TST
Positive Likelihood Ratio (CI95%)	3.87 (2.81–5.34)	3.04 (2.32–3.97)
Negative Likelihood Ratio (CI95%)	0.66 (0.59–0.73)	0.51 (0.44–0.60)
Positive Predictive Value (pos-test probability) of each test individually	64%	60%
Pos-test probability if IGRA and TST are positive	84%	

Despite this increment, performing both tests is not mandatory or recommended, and the treatment of the underlying IMID should not be delayed. According to statement 4, either of the tests, TST or IGRA, can be used for TBI diagnosis.

 **LOA: 62.5% Strongly agree; 35% Agree. Sum of the percentage of “Strongly agree” and “Agree”: 97,5%. Overall quality of evidence across all critical outcomes: Moderate.**

Recommendation 6. In the event of an indeterminate IGRA test result, it is recommended to repeat the test as soon as possible. If the result remains inconclusive, consider TPT.

The observational studies included in this analysis showed a wide range of IGRA indeterminate results, from 2.1 to 31.5%. Some disease and treatment-associated factors, as well as methodological issues may have contributed to an indeterminate IGRA result, such as living in an endemic area for TBD, glucocorticoid and other immunosuppressive treatments, disease activity, older age, low serum protein and albumin, lymphopenia, and high C-reactive protein (CRP) levels [40–48].

In one study with 190 IBD patients, indeterminate IGRA occurred in 26 patients (13.7%). All 26 patients with indeterminate IGRA had a negative TST. Twenty-four of 26 patients received TNFi, with no cases of TB [44, 45]. At the same time, there were also controversial TBD outcomes related to an indeterminate IGRA, corroborating the present recommendation to repeat the test as soon as possible and consider TPT if the test remains inconclusive [40–48].

 **LOA: 62.5% Strongly agree; 35% Agree. Sum of the percentage of “Strongly agree” and “Agree”: 97,5%. Overall quality of evidence across all critical outcomes: Moderate.**

Recommendation 7. If the pre-treatment TST/IGRA test is negative, annual repetition of the test is recommended until the third year of treatment, especially in IMID patients taking TNFi. After this period, clinical and epidemiological surveillance is recommended during the immunosuppressive treatment, regardless of the class. In persons with a previous history of treatment for TBI or TBD, screening should not be repeated.

In the studies included in this analysis, all the patients had negative TBI tests (TST and/or IGRA) at baseline. The mean conversion rate after TNFi exposure was 12.8% over a mean time of 12.4 months (95% CI 8.8–17.4 months). Of patients who converted, 4% developed TBD. However, no prospective randomized clinical trials or observational cohorts demonstrated the effectiveness of different intervals for TST/IGRA conversion and incident TBD as the primary outcome related to the use of the different classes of DMARDs.

TBD diagnosis occurs early (less than three years after starting DMARDs, mainly TNFi) in most patients. Jauregui-Amezaga evaluated the risk of developing TBD in IBD patients under TNFi treatment, despite TBI screening. During the study period, 423 patients received TNFi therapy. Screening for TBI before TNFi treatment was positive in 30 patients (6.96%). Seven patients (1.65%) developed TBD while under TNFi treatment. In 4 of these patients, TBD was diagnosed within the first 16 weeks after starting TNFi therapy [33].

There is no consensus on the ideal timing for repeating TBI tests or how many times it should be performed. Although robust data on the effectiveness or cost-effectiveness of this strategy in clinical practice has yet to be collected, the ACR/EULAR has guided the annual repetition of the tests during TNFi/immunosuppressive treatment [49]. The Brazilian Ministry of Health recommends repetition of the test one year after the introduction of the immunosuppressive treatment, if the initial tests were negative [7].

Some aspects should be considered regarding this issue. The studies have heterogeneous results, such as different IMIDs (RA, axial SpA, PsA, vasculitis, juvenile idiopathic arthritis, PsO, and IBD), sample size, and follow-up time. The TST conversion rate may vary among IMIDs because of different pathophysiological characteristics, signaling pathways of the diseases themselves, impairment of cellular response, including anergy, and the different doses and classes of DMARDs and glucocorticoids. Finally, there is a lack of information regarding therapeutic measures introduced after the TST conversion rate, concerning TBD diagnosis, in the context of switching among biological DMARDs (TNFi versus non-TNFi), and TNFi discontinuation.

Two main hypotheses could better explain the higher positivity regarding the TST conversion rate after exposure to DMARDs. The main reason is that it would be directly related to re-exposure or new contact with the bacillus, since patients were previously negative and became positive with repetition. In this case, we would expect the frequency of TBD to increase over time, but most cases occur in the first 1–3 years, even in screened patients. Moreover, it could also be associated with an improved cellular response after immunosuppressive treatment, as well as reduction of glucocorticoid dosage and better control of disease activity with DMARDs [50, 51].

Indiscriminate repetition generates higher direct and indirect public health costs, especially in countries with cyclical shortages of TST. Therefore, prospective observational studies and randomized controlled clinical trials are needed to better define the effectiveness of repeating the TST.

This committee recommends the annual repetition of TST/IGRA during the first three years of DMARD treatment, mainly with TNFi. If either of the tests is positive, TPT should be carried out. After this period, clinical and epidemiological surveillance is recommended.

 **LOA: 32.5% Strongly agree; 52.5% Agree. Sum of the percentage of “Strongly agree” and “Agree”: 85%. Overall quality of evidence across all critical outcomes: Moderate.**

Recommendation 8. In IMID, if it is necessary to change the medication, regardless of the class, if there is a previous negative TBI screening, TST/IGRA should be performed annually for the next 3 years, according to recommendation 7.

There is a lack of information regarding TST or IGRA repetition when a specific therapeutic decision needs to be made, due to inadequate response or side effects related to current treatment. Some aspects must be considered, such as disease activity and severity, comorbidities, concomitant medication, and other safety aspects. Moreover, some details could interfere with the need to rescreen patients for TBI diagnosis, such as the baseline result of the tests, time since the first screening, preventive measures implemented, and the order of bDMARDs used [50, 51].

For patients with positive baseline screening and adequate proven treatment for TBI, the repetition of TST or IGRA is not necessary. Similar recommendations may be made for those with a previous history of TBD who were adequately treated [1].

Considering the TST/ IGRA conversion rate in IMIDs during immunosuppressive therapy, the repetition might be considered in patients with negative baseline screening who need to change DMARDs, especially for increasing the detection of initial false-negative cases and to enhance the identification of new cases after exposure to these drugs. If the patient is on TNFi and does not develop TBD, the risk may be reduced over time if changed to a non-TNFi agent. However, if the change is for a TNFi, there could be a higher TBD risk.

Therefore, prospective observational studies and randomized controlled clinical trials are needed to better define the effectiveness of repeating the TST/ IGRAs in this scenario.

 **LOA: 32.5% Strongly agree; 52.5% Agree. Sum of the percentage of “Strongly agree” and “Agree”: 85%. Overall quality of evidence across all critical outcomes: Very Low.**

Recommendation 9. In persons with IMID vaccinated with BCG in the two years before starting immunosuppressive treatment, IGRA is preferable to TST for TBI screening. If BCG was administered more than two years before the introduction of treatment, a positive TST or IGRA result should be interpreted as a diagnosis of TBI and TPT should be started as soon as TBD is ruled out.

The BCG vaccine, derived from a live attenuated strain of *Mycobacterium bovis*, protects against severe and disseminated forms of TBD, and its introduction into national immunization programs around the world has dramatically changed the epidemiology of this disease, especially in the pediatric age group. Its application, however, can cause cross-reaction with the TST in an estimated proportion of up to 8.5 false-positive tests for every 100 individuals vaccinated [52, 53].

The magnitude of the impact of BCG on TST results is still unclear. According to Menzies et al., over 90% of those vaccinated with BCG will present a TST > 10 mm approximately 8 to 12 weeks after the vaccine application. However, the duration of this effect has yet to be entirely elucidated. While some studies suggest that BCG may influence TST reactivity for periods ranging from 4 to 25 years after immunization, other studies show that “in high TBD burden countries” the effect of BCG on the TST response in adults is negligible, regardless of age at vaccination. In persons with IMID, BCG vaccination is an independent predictive factor for a greater induration of TST [54].

However, there is concordance among the specialists that the effect of BCG on TST positivity wanes after two years. Thus, in children younger than two years vaccinated with BCG, performing TST as screening for TBI should be avoided due to the high risk of false-positive results, according to the American Academy of Pediatrics [55]. At the same time, IGRA tests are also not indicated in this age group, although recent evidence suggests its high accuracy.

IGRA is the best test for TBI screening for patients vaccinated with BCG in the previous year, especially in the pediatric age group [56]. However, in patients with IMID, the proportion of indeterminate IGRA is higher than in the general population, as stated in recommendation 6. This effect varies according to the diagnosis (being more frequent in lupus patients), degree of disease activity, and immunosuppressive therapeutic regimen [57].

A higher proportion of TST positivity may be attributed to BCG in countries where BCG is administered repeatedly. For example, TST positivity in Korea and Japan reduced after these countries reduced the number of BCG doses in their national vaccination programs [58]. In Brazil, the current practice is only one dose of BCG, but repeated BCG can be used for leprosy [59]. In this scenario, a positive TST in patients who will be started on DMARDs should be interpreted as TBI and TPT prescribed, regardless of age.

BCG can also impact the TST result in patients tested repeatedly, an effect known as booster response, especially in the first 15 years after vaccination. Individuals vaccinated with BCG are 1.5 times more likely to present the booster effect within one week to 1 year after performing the previous TST [60].

The Centers for Disease Control and Prevention (CDC) recommends the preferential use of the IGRA among people vaccinated with BCG to increase the diagnosis’s specificity and reduce the proportion of false positive diagnoses [61]. In low-income countries, the routine performance of IGRA for TBI screening may be impractical due to the high costs of the test and the need for specialized laboratories. In these cases, a two-step approach is recommended, indicating the performance of IGRA in patients vaccinated with BCG who have a positive TST, especially in children younger than five years.

In conclusion, this committee recommends that in adults or children candidates for DMARD introduction, a TST ≧ 5 mm, if BCG was administered more than two years before, or in the context of a positive IGRA, TBI should be considered and TPT started after TBD is excluded.

 **LOA: 70% Strongly Agree, 30% Agree. Sum of the percentage of “Strongly agree” and “Agree”: 100%. Overall quality of evidence across all critical outcomes: Low.**

## Conclusions

This task force, which included several experts dealing with IMID and their relationship to TBD, has proposed recommendations for the diagnosis and management of this condition, based on a systematic review and consensus of experts and according to the Brazilian scenario related to TBI and TBD.

These recommendations have been created to help healthcare professionals manage patients with IMID, but they must consider the epidemiological risk, host features, the social scenario, the characteristics of the disease, the access to health resources, and the development of an individualized plan of action for every patient, taking in consideration the patient’s perspective and the management of their underlying condition.

It is of paramount importance to consider the limitations related to the scarcity of robust evidence on many of the issues discussed, highlighting the need for further studies that evaluate TBD and TBI in IMID.

## Electronic supplementary material

Below is the link to the electronic supplementary material.

Supplementary Material 1

Supplementary Material 2

## Data Availability

Data is provided within the manuscript and supplementary material files (Appendix 1 and 2).

## Abbreviations

TBD: Tuberculosis disease
Mtb: Mycobacterium tuberculosis
WHO: World Health Organization
TBI: Tuberculosis infection
IMID: Immune mediated inflammatory disease
LWHIV: Living with human immunodeficiency virus
TNF: Tumor necrosis fator
RA: Rheumatoid arthritis
PsA: Psoriatic arthritis
SpA: Axial spondyloarthritis
PsO: Psoriasis
IBD: Inflammatory bowel disease
CD: Crohn disease
UC: Ulcerative colitis
DMARDs: Disease-modifying antirheumatic drugs
bDMARD: Biological DMARDs
tsDMARD: Targeted-synthetic DMARDs
csDMARD: Conventional synthetic DMARDs
TNFi: Tumor necrosis factor inhibitors
RTX: Rituximab
ABA: Abatacept
UTK: Ustekinumab
SEC: Secukinumab
IXK: Ixekizumab
RIZ: Risankizumab
GUS: Guselkumab
GRADE: Grading of Recommendations, Assessment, Development, and Evaluations
BCG: Bacillus Calmette-Guérin
HCP: Healthcare provider
IGRA: Interferon-gamma release assay
LoA: Level of agreement
TPT: Tuberculosis preventive treatment
TST: Tuberculin skin test

## References

[1] Saude Md. Manual de recomendações para o controle da tuberculose no Brasil. In: transmissíveis. SdVeSDdVdd, editor. http://bvsms.saude.gov.br/bvs/publicacoes/manual_recomendacoes_controle_tuberculose_brasil_2_ed.pdf.2019

[2] WHO. WHO Global Tuberculosis Report 2023. In. WHO, editor. https://iris.who.int/bitstream/handle/10665/373828/9789240083851-eng.pdf?sequence=12023

[3] Saúde Md. Boletim Epidemiológico número especial. Brasil livre da tuberculose: evolução dos cenários epidemiológicos e operacionais da doença.In: Saúde. SdVe, editor. 2021. https://www.gov.br/saude/pt-br/centrais-de

[4] Coussens AK, Zaidi SMA, Allwood BW, Dewan PK, Gray G, Kohli M, et al. Classification of early tuberculosis States to guide research for improved care and prevention: an international Delphi consensus exercise. Lancet Respir Med. 2024;12(6):484–98. 38527485 10.1016/S2213-2600(24)00028-6 PMC7616323

[5] Houben RM, Dodd PJ. The global burden of latent tuberculosis infection: A Re-estimation using mathematical modelling. PLoS Med. 2016;13(10):e1002152. 27780211 10.1371/journal.pmed.1002152 PMC5079585

[6] Campbell JR, Winters N, Menzies D. Absolute risk of tuberculosis among untreated populations with a positive tuberculin skin test or interferon-gamma release assay result: systematic review and meta-analysis. BMJ. 2020;368:m549. 32156698 10.1136/bmj.m549 PMC7190060

[7] NOTA INFORMATIVA Nº 4/2024-CGTM/.DATHI/SVSA/MS. Recomendações técnicas aos enfermeiros para orientar a indicação do tratamento da Infecção Latente da Tuberculose (ILTB), os algoritmos para idenficação e rastreio da ILTB, além de recomendações sobre o tratamento da infecção latente pelo Mycobacterium tuberculosis. Disponível em Acesso em 02 de setembro de 2024. https://www.gov.br/aids/pt-br/central-de-conteudo/notas-informativas/2024/nota-informativa-no-42024-cgtm-dathisvsa.pdf

[8] Goletti D, Petrone L, Ippolito G, Niccoli L, Nannini C, Cantini F. Preventive therapy for tuberculosis in rheumatological patients undergoing therapy with biological drugs. Expert Rev Anti Infect Ther. 2018;16(6):501–12. 29848120 10.1080/14787210.2018.1483238

[9] Gasparin AA, dAN, Hax V, et al. In: Moreira CSS, editor. Imunossupressores e Imunomoduladores. Livro da Sociedade Brasileira de Reumatologia manole editora; 2023.

[10] Cantini F, Nannini C, Niccoli L, Iannone F, Delogu G, Garlaschi G, et al. Guidance for the management of patients with latent tuberculosis infection requiring biologic therapy in rheumatology and dermatology clinical practice. Autoimmun Rev. 2015;14(6):503–9. 25617816 10.1016/j.autrev.2015.01.011

[11] Keane J, Gershon S, Wise RP, Mirabile-Levens E, Kasznica J, Schwieterman WD, et al. Tuberculosis associated with Infliximab, a tumor necrosis factor alpha-neutralizing agent. N Engl J Med. 2001;345(15):1098–104. 11596589 10.1056/NEJMoa011110

[12] Evangelatos G, Koulouri V, Iliopoulos A, Fragoulis GE. Tuberculosis and targeted synthetic or biologic DMARDs, beyond tumor necrosis factor inhibitors. Ther Adv Musculoskelet Dis. 2020;12:1759720X20930116. 32612710 10.1177/1759720X20930116 PMC7309385

[13] Cantini F, Niccoli L, Capone A, Petrone L, Goletti D. Risk of tuberculosis reactivation associated with traditional disease modifying anti-rheumatic drugs and non-anti-tumor necrosis factor biologics in patients with rheumatic disorders and suggestion for clinical practice. Expert Opin Drug Saf. 2019;18(5):415–25. 31066297 10.1080/14740338.2019.1612872

[14] Torres T, Chiricozzi A, Puig L, Le AM, Marzano AV, Dapavo P, et al. Treatment of psoriasis patients with latent tuberculosis using IL-17 and IL-23 inhibitors: A retrospective, multinational, multicentre study. Am J Clin Dermatol. 2024;25(2):333–42. 38265746 10.1007/s40257-024-00845-4 PMC10867072

[15] Schunemann HJ, Cuello C, Akl EA, Mustafa RA, Meerpohl JJ, Thayer K, et al. GRADE guidelines: 18. How ROBINS-I and other tools to assess risk of bias in nonrandomized studies should be used to rate the certainty of a body of evidence. J Clin Epidemiol. 2019;111:105–14. 29432858 10.1016/j.jclinepi.2018.01.012 PMC6692166

[16] Sterne JAC, Savovic J, Page MJ, Elbers RG, Blencowe NS, Boutron I, et al. RoB 2: a revised tool for assessing risk of bias in randomised trials. BMJ. 2019;366:l4898. 31462531 10.1136/bmj.l4898

[17] Schünemann H, Brożek J, Guyatt G, Oxman A, editors. GRADE handbook for grading quality of evidence and strength of recommendations. Updated October 2013. The GRADE Working Group, 2013. Available from guidelinedevelopment.org/handbook.).

[18] Santesso N, Glenton C, Dahm P, Garner P, Akl EA, Alper B, et al. GRADE guidelines 26: informative statements to communicate the findings of systematic reviews of interventions. J Clin Epidemiol. 2020;119:126–35. 31711912 10.1016/j.jclinepi.2019.10.014

[19] Hasson F, Keeney S, McKenna H. Research guidelines for the Delphi survey technique. J Adv Nurs. 2000;32(4):1008–15. 11095242

[20] Likert R. A technique for the measurement of attitudes. Archives Psychol. 1932;22(140):55.

[21] Brouwers MC, Kerkvliet K, Spithoff K, on behalf of the AGREE Next Steps Consortium. The AGREE reporting checklist: a tool to improve reporting of clinical practice guidelines. BMJ. 2016;352:i1152. 10.1136/bmj.i1152. 26957104 10.1136/bmj.i1152 PMC5118873

[22] Lauper K, Kearsley-Fleet L, Galloway JB, Watson KD, Hyrich KL, Lunt M, BSRBR-RA Contributors Group. Evaluation of serious infections, including Mycobacterium tuberculosis, during treatment with biologic disease-modifying anti-rheumatic drugs: does line of therapy matter?. 2024;63(7):1957–1964. 10.1093/rheumatology/kead515 PMC11215981 37758229

[23] Torres T, Brembilla NC, Langley RG, Warren RB, Thaci D, Kolios AGA, et al. Treatment of psoriasis with biologic and non-biologic targeted therapies in patients with latent tuberculosis infection or at risk for tuberculosis disease progression: recommendations from a SPIN-FRT expert consensus. J Eur Acad Dermatol Venereol. 2024 Aug;16. 10.1111/jdv.20287. 10.1111/jdv.20287 39149807

[24] Choi SR, Shin A, Ha Y, Lee YJ, Lee EB, Kang EH. Comparative risk of infections between JAK inhibitors versus TNF inhibitors among patients with rheumatoid arthritis: a cohort study. Arthritis Res Ther. 2023;25(1):129. 37495973 10.1186/s13075-023-03111-w PMC10369724

[25] Chiu YM, Chen DY. Infection risk in patients undergoing treatment for inflammatory arthritis: non-biologics versus biologics. Expert Rev Clin Immunol. 2020;16(2):207–28. 31852268 10.1080/1744666X.2019.1705785

[26] Agarwal SK. Biologic agents in rheumatoid arthritis: an update for managed care professionals. J Manag Care Pharm. 2011;17(9 B):S14–8. 22073935 10.18553/jmcp.2011.17.s9-b.S14 PMC10441186

[27] Chung TT, Ko HJ, Lau CS, Chung HY. A retrospective study on the risk of tuberculosis in patients with rheumatoid arthritis. Rheuma Int. 2020;40(6):983–90. 10.1007/s00296-020-04583-8 32318800

[28] Liu T, Yan H, Gao M. Tuberculosis infection in patients with rheumatic diseases under different treatments. J Infect Public Health. 2025;18(5):102703. 40020472 10.1016/j.jiph.2025.102703

[29] Turetz ML, Ma KC. Diagnosis and management of latent tuberculosis. Curr Opin Infect Dis. 2016;29(2):205–11. 26836374 10.1097/QCO.0000000000000253

[30] Getahun H, Matteelli A, Abubakar I, Aziz MA, Baddeley A, Barreira D, et al. Management of latent Mycobacterium tuberculosis infection: WHO guidelines for low tuberculosis burden countries. Eur Respir J. 2015;46(6):1563–76. 26405286 10.1183/13993003.01245-2015 PMC4664608

[31] Getahun H, Matteelli A, Chaisson RE, Raviglione M. Latent Mycobacterium tuberculosis infection. N Engl J Med. 2015;372(22):2127–35. 26017823 10.1056/NEJMra1405427

[32] Reichler MR, Khan A, Yuan Y, Chen B, McAuley J, Mangura B, et al. Duration of exposure among close contacts of patients with infectious tuberculosis and risk of latent tuberculosis infection. Clin Infect Dis. 2020;71(7):1627–34. 32044987 10.1093/cid/ciz1044 PMC8851607

[33] Jauregui-Amezaga A, Turon F, Ordas I, Gallego M, Feu F, Ricart E, et al. Risk of developing tuberculosis under anti-TNF treatment despite latent infection screening. J Crohns Colitis. 2013;7(3):208–12. 22677117 10.1016/j.crohns.2012.05.012

[34] Kurt OK, Kurt B, Talay F, Tug T, Soy M, Bes C, et al. Intermediate to long-term follow-up results of INH chemoprophylaxis prior to anti-TNF-alpha therapy in a high-risk area for tuberculosis. Wien Klin Wochenschr. 2013;125(19–20):616–20. 24061693 10.1007/s00508-013-0417-0

[35] Alasan F, Gulec Balbay E, Cangur S, Balbay O, Yilmaz Aydin L, Annakkaya AN. Should Isoniazid prophylaxis be prescribed to the patients under tumor necrosis factor-alpha antagonists independent of tuberculin skin test? Aging Male. 2020;23(5):1109–14. 31615316 10.1080/13685538.2019.1678582

[36] da Mota LM, Cruz BA, de Albuquerque CP, Goncalves D, Laurindo IM, Pereira IA, et al. [Preliminary guidelines of the Brazilian society of rheumatology for evaluation and treatment of tuberculosis latent infection in patients with rheumatoid arthritis, in face of unavailability of the tuberculin skin test]. Rev Bras Reumatol. 2015;55(4):390–3. 26165801 10.1016/j.rbr.2015.01.006

[37] WHO. Latent tuberculosis infection – Updated and consolidated guidelines for programmatic management. 2024 https://www.who.int/publications/i/item/97892400015032020.2020 30277688

[38] Jeong DH, Kang J, Jung YJ, Yoo B, Lee CK, Kim YG, et al. Comparison of latent tuberculosis infection screening strategies before tumor necrosis factor inhibitor treatment in inflammatory arthritis: IGRA-alone versus combination of TST and IGRA. PLoS ONE. 2018;13(7):e0198756. 29975703 10.1371/journal.pone.0198756 PMC6033383

[39] Bartalesi F, Vicidomini S, Goletti D, Fiorelli C, Fiori G, Melchiorre D, et al. QuantiFERON-TB gold and the TST are both useful for latent tuberculosis infection screening in autoimmune diseases. Eur Respir J. 2009;33(3):586–93. 19047313 10.1183/09031936.00107608

[40] Rousset S, Treiner E, Moulis G, Pugnet G, Astudillo L, Paricaud K, et al. High rate of indeterminate results of the QuantiFERON-TB gold in-tube test, third generation, in patients with systemic vasculitis. Rheumatology (Oxford). 2020;59(5):1006–10. 31518431 10.1093/rheumatology/kez390

[41] Kaur M, Singapura P, Kalakota N, Cruz G, Shukla R, Ahsan S, et al. Factors that contribute to indeterminate results from the QuantiFERON-TB gold In-Tube test in patients with inflammatory bowel disease. Clin Gastroenterol Hepatol. 2018;16(10):1616–21. e1. 29175527 10.1016/j.cgh.2017.11.038

[42] Vajravelu RK, Osterman MT, Aberra FN, Roy JA, Lichtenstein GR, Mamtani R, et al. Indeterminate QuantiFERON-TB gold increases likelihood of inflammatory bowel disease treatment delay and hospitalization. Inflamm Bowel Dis. 2017;24(1):217–26. 29272482 10.1093/ibd/izx019 PMC7007987

[43] Hradsky O, Ohem J, Zarubova K, Mitrova K, Durilova M, Kotalova R, et al. Disease activity is an important factor for indeterminate interferon-gamma release assay results in children with inflammatory bowel disease. J Pediatr Gastroenterol Nutr. 2014;58(3):320–4. 24126833 10.1097/MPG.0000000000000205

[44] Papay P, Eser A, Winkler S, Frantal S, Primas C, Miehsler W, et al. Predictors of indeterminate IFN-gamma release assay in screening for latent TB in inflammatory bowel diseases. Eur J Clin Invest. 2011;41(10):1071–6. 21413978 10.1111/j.1365-2362.2011.02502.x

[45] Papay P, Eser A, Winkler S, Frantal S, Primas C, Miehsler W, et al. Factors impacting the results of interferon-gamma release assay and tuberculin skin test in routine screening for latent tuberculosis in patients with inflammatory bowel diseases. Inflamm Bowel Dis. 2011;17(1):84–90. 20722065 10.1002/ibd.21427

[46] Magri GKPMG. Azzopardi N. Predictor factors for an indeteminate result on interfereon -gamma release assay. Gut. 2016(A146)

[47] Wolf WACC, Runge T, Eluri S, Weimer E, Schmitz J, Herfarth HH. Immunosuppression increases the odds of an indeterminate tuberculosis screen. Gastroenterology. 2015;148(4):S473.

[48] Nasr IGR, Ward M, Fong S, Patel K, Sastrillo M, et al. Indeterminate and inconclusive results are common when using interferon-gamma release assay as screening for TB in patients with IBD. Gut. 2014;63(june):A77.

[49] Fragoulis GE, Nikiphorou E, Dey M, Zhao SS, Courvoisier DS, Arnaud L, et al. 2022 EULAR recommendations for screening and prophylaxis of chronic and opportunistic infections in adults with autoimmune inflammatory rheumatic diseases. Ann Rheum Dis. 2023;82(6):742–53. 36328476 10.1136/ard-2022-223335

[50] Bautista-Molano W, Gonzalez L, Fernandez-Avila D, Cardozo R, Ruiz O. Frequency of positivity of the tuberculin intradermorreaction test in a cohort of patients with rheumatoid arthritis. Biomedica. 2021;41(3):472–80. 34559494 10.7705/biomedica.5416 PMC8519591

[51] Shimabuco AY, Medeiros-Ribeiro AC, Miossi R, Bonfiglioli KR, Moraes JCB, Goncalves CR, et al. Ankylosing spondylitis and psoriatic arthritis: revisiting screening of latent tuberculosis infection and its follow-up during anti-tumor necrosis factor therapy in an endemic area. Clin (Sao Paulo). 2020;75:e1870. 10.6061/clinics/2020/e1870 PMC7561058 33146355

[52] Mancuso JD, Mody RM, Olsen CH, Harrison LH, Santosham M, Aronson NE. The Long-term effect of Bacille Calmette-Guerin vaccination on tuberculin skin testing: A 55-Year Follow-Up study. Chest. 2017;152(2):282–94. 28087302 10.1016/j.chest.2017.01.001

[53] Farhat M, Greenaway C, Pai M, Menzies D. False-positive tuberculin skin tests: what is the absolute effect of BCG and non-tuberculous mycobacteria? Int J Tuberc Lung Dis. 2006;10(11):1192–204. 17131776

[54] Menzies D. What does tuberculin reactivity after Bacille Calmette-Guerin vaccination tell Us?? Clin Infect Dis. 2000;31(Suppl 3):S71–4. 11010826 10.1086/314075

[55] Nolt D, Starke JR. Tuberculosis infection in children and adolescents: testing and treatment. Pediatrics. 2021;148(6). 10.1542/peds.2021-054663 34851422

[56] Gabriele F, Trachana M, Simitsopoulou M, Pratsidou-Gertsi P, Iosifidis E, Pana ZD, et al. Performance of QuantiFERON(R)-TB gold In-Tube assay in children receiving disease modifying anti-rheumatic drugs. World J Pediatr. 2017;13(5):472–8. 28646434 10.1007/s12519-017-0050-5

[57] Cho H, Kim YW, Suh CH, Jung JY, Um YJ, Jung JH, et al. Concordance between the tuberculin skin test and interferon gamma release assay (IGRA) for diagnosing latent tuberculosis infection in patients with systemic lupus erythematosus and patient characteristics associated with an indeterminate IGRA. Lupus. 2016;25(12):1341–8. 26985011 10.1177/0961203316639381

[58] Lee SW, Oh SY, Lee JB, Choi CM, Kim HJ. Tuberculin skin test distribution following a change in BCG vaccination policy. PLoS ONE. 2014;9(1):e86419. 24466082 10.1371/journal.pone.0086419 PMC3900524

[59] Saude Md. Manual dos Centros de Referência para Imunobiológicos Especiais / In: Secretaria de Vigilância em Saúde e Ambiente dIeDI, Coordenação-Geral do Programa Nacional de Imunizações, editor. 6 edition ed. https://www.gov.br/saude/pt-br/vacinacao/arquivos/manual-dos-centros-de-referencia-para-imunobiologicos-especiais_6a-edicao_2023.pdf2023.

[60] Hizel K, Maral I, Karakus R, Aktas F. The influence of BCG immunisation on tuberculin reactivity and booster effect in adults in a country with a high prevalence of tuberculosis. Clin Microbiol Infect. 2004;10(11):980–3. 15522000 10.1111/j.1469-0691.2004.00970.x

[61] CDC. Latent TB infection testing and treatment summary of U.S. recommendations. In: Services UDoHaH, editor. 2020.

