# Correction: Brazilian recommendations for the management of tuberculosis infection in immune-mediated inflammatory diseases

**DOI:** 10.1186/s42358-025-00466-3

**Published:** 2025-07-21

**Authors:** Viviane Angelina de Souza, Ana Luiza Mendes Amorim Caparroz, Virginia Fernandes Moça Trevisani, Anna Carolina Faria Moreira Gomes Tavares, Ana Karla Guedes de Melo, Anete Trajman, Ana Cristina de Medeiros-Ribeiro, Marcelo de Medeiros Pinheiro, Ricardo Machado Xavier, Odirlei Andre Monticielo, Maria Fernanda Brandão de Resende Guimarães, Flavio Sztajnbok, Sidney Bombarda, Liliana Andrade Chebli, Adriana Maria Kakehasi, Ana Luiza Bierrenbach, Ana Paula Monteiro Gomides Reis, Blanca Elena Rios Gomes Bica, Claudia Diniz Lopes Marques, Cristina Flores, Denise Silva Rodrigues, Eduardo dos Santos Paiva, Eliana Dias Matos, Fernanda Dockhorn Costa Johansen, Helio Arthur Bacha, Joana Starling de Carvalho, José Roberto Provenza, Ketty Lysie Libardi Lira Machado, Licia Maria Henrique da Mota, Lilian David de Azevedo Valadares, Marco Antônio Araújo da Rocha Loures, Margareth Maria Pretti Dalcolmo, Maria Cecilia de Carvalho Bortoletto, Max Igor Banks Ferreira Lopes, Rejane Maria Rodrigues de Abreu Vieira, Ricardo Romiti, Rogerio Saad-Hossne, Rozana Mesquita Ciconelli, Valderilio Feijó Azevedo, Valéria Maria Augusto, Vitor Alves Cruz, Gecilmara Cristina Salviato Pileggi

**Affiliations:** 1 Hospital Universitário da Universidade Federal de Juiz de Fora, Juiz de Fora, Brazil; 2 Faculdade de Medicina de São José do Rio Preto, São José do Rio Preto, Brazil; 3 Universidade de Santo Amaro UNISA, Santo Amaro, Brazil; 4 Universidade Federal de São Paulo (UNIFESP/ EPM), São Paulo, Brazil; 5 Hospital das Clínicas -Universidade Federal de Minas Gerais, Belo Horizonte, Brazil; 6 Hospital Universitário Lauro Wanderley - Universidade Federal da Paraíba, João Pessoa, Brazil; 7 Universidade Federal do Rio de Janeiro (UFRJ), Rio de Janeiro, Brazil; 8 Hospital das Clínicas da Faculdade de Medicina da Universidade de São Paulo, São Paulo, Brazil; 9 Hospital de Clínicas de Porto Alegre - Universidade Federal do Rio Grande do Sul, Porto Alegre, Brazil; 10 Secretaria de Estado da Saúde de São Paulo, São Paulo, Brazil; 11 Faculdade de Medicina da Universidade Federal de Minas Gerais, Belo Horizonte, Brazil; 12 Instituto de Ensino e Pesquisa - Hospital Sírio-Libanês, São Paulo, Brazil; 13 Centro Universitário de Brasília, Brasília, Brazil; 14 Hospital das Clinicas - Universidade Federal de Pernambuco, Recife, Brazil; 15 Centro de Tratamento de Doenças Inflamatórias Intestinais e Imunomediadas, Porto Alegre, Brazil; 16 Instituto Clemente Ferreira - São Paulo, São Paulo, Brazil; 17 Hospital de Clinicas da Universidade Federal do Paraná, Curitiba, Brazil; 18 Hospital Especializado Octávio Mangabeira (Bahia), Salvador, Brazil; 19 Coordenação-Geral de Vigilância da Tuberculose, Micoses Endêmicas e Micobactérias não Tuberculosas, (Ministério da Saúde), Brasília, Brazil; 20 Hospital Israelita Albert Einstein, São Paulo, Brazil; 21 Hospital Universitário da PUC Campinas, Campinas, Brazil; 22 Hospital Universitário Cassiano Antonio Moraes da Universidade Federal do Espírito Santo (UFES), Vitória, Brazil; 23 Hospital Universitário de Brasília, Universidade de Brasília, Brasília, Brazil; 24 Hospital Getulio Vargas de Pernambuco, Recife, Brazil; 25 Universidade Estadual de Maringá/UEM, Maringá, Brazil; 26 Centro de Referência Professor Hélio Fraga – Fiocruz, Rio de Janeiro, Brazil; 27 Hospital Servidor Municipal São Paulo, São Paulo, Brazil; 28 Universidade de Fortaleza (UNIFOR), Fortaleza, Brazil; 29 Universidade Estadual do Ceará (UECE), Fortaleza, Brazil; 30 Faculdade de Medicina de Botucatu - Universidade Estadual Paulista– UNESP, São Paulo, Brazil; 31 Hospital Beneficência Portuguesa de São Paulo, São Paulo, Brazil; 32 Universidade Federal de Goiás (UFG), Goiás, Brazil; 33 Instituto D’Or de Pesquisa e Ensino-UNISA, São Paulo, Brazil

**Correction: Advances in Rheumatology (2025) 65:18**

10.1186/s42358-025-00449-4

The original publication of this article contained an error in the competing interests section. The incorrect and correct competing interests section are shown in this correction article. The original article has been updated.

Incorrect competing interests

The authors declare that the research was carried out in the absence of any commercial or financial relationship that could be interpreted as a potential conflict of interest. Several authors of these guidelines, including voting members, have interacted with the pharmaceutical industry including the manufacturers of some of the drugs mentioned in these recommendations. However, none of the authors received any support or fee directly or indirectly related to or influencing the development of these guidelines. AKGM received speaker fees and consulting from Abbvie, Johnson & Johnson. AMK received consuting fees from Abbvie, Lilly, UCB, Organon, and Janssen. CF received speaker fees from Takeda, Janssen, Abbvie, Amgen, Organon. ESP received speaker fees and consulting from Abbvie, Novartis, Lilly, Johnson & Johnson. JSC received speaker fees from GSK and Janssen. KLLLM received speaker fees and consulting from Novartis and Johnson & Johnson. LAC received speaker fees and consulting from Johnson & Johnson, Takeda, Abbvie. LDAV received speaker fees and consulting from Novartis, Johnson & Johnson. LMHM received speaker fees and consulting from Johnson & Johnson, Abbvie, Pfizer, Novartis, Amgen, Organon, Celltrion, UCB. MFBRG received speaker fees and consulting from Johnson & Johnson, Abbvie, Amgen, Lilly, UCB. MIBFL received speaker fees and consulting from Abbvie, Novartis, Takeda, Johnson & Johnson. MMP received consuting fees from Abbvie, Lilly and Janssen. RMX received Speaker fees, consulting, research funding from Abbvie, Pfizer, Amgen, Biogen, Novartis, Janssen, UCB. RR is/has served as a scientific consultant, speaker, or clinical study investigator for AbbVie, Boehringer Ingelheim, Bristol Myers Squibb, Galderma, Janssen-Cilag, Eli-Lilly, Leo-Pharma, Novartis, Pfizer, Sanofi, and UCB. RSH received speaker fees and consulting from Abbvie, Novartis, Takeda, Johnson & Johnson. OAM received consulting fees from AstraZeneca and GSK; received payment or honoraria for lectures or presentations from AbbVie, Janssen, GSK, UCB and AstraZeneca; and participated in a data safety monitoring board or advisory board for GSK, CellTrion, Bristol, Novartis, Janssen, and AstraZeneca. VAS received speaker fees and consulting from Abbvie, Novartis, Lilly, Johnson & Johnson, GSK, Astrazeneca. VFA received speaker fees, consulting, clinical researches from Celltiron, Amgen, Janssen, Organon, Sandoz, Abbvie, Fresenius Kabi, Takeda, LG, Boehringer-Ingelheim, Bristol Myers-Squibb, GSK, AstraZeneca. The authors not mentioned above declare no competing interests.

Correct competing interests

The authors declare that the research was carried out in the absence of any commercial or financial relationship that could be interpreted as a potential conflict of interest. Several authors of these guidelines, including voting members, have interacted with the pharmaceutical industry including the manufacturers of some of the drugs mentioned in these recommendations. However, none of the authors received any support or fee directly or indirectly related to or influencing the development of these guidelines. AKGM received speaker fees and consulting from Abbvie, Johnson & Johnson. AMK received consuting fees from Abbvie, Lilly, UCB, Organon, and Janssen. CF received speaker fees from Takeda, Janssen, Abbvie, Amgen, Organon. ESP received speaker fees and consulting from Abbvie, Novartis, Lilly, Johnson & Johnson. JSC received speaker fees from GSK and Janssen. KLLLM received speaker fees and consulting from Novartis and Johnson & Johnson. LAC received speaker fees and consulting from Johnson & Johnson, Takeda, Abbvie. LDAV received speaker fees and consulting from Novartis, Johnson & Johnson. LMHM received speaker fees and consulting from Johnson & Johnson, Abbvie, Pfizer, Novartis, Amgen, Organon, Celltrion, UCB. MFBRG received speaker fees and consulting from Johnson & Johnson, Abbvie, Amgen, Lilly, UCB. MIBFL received speaker fees and consulting from Abbvie, Novartis, Takeda, Johnson & Johnson. MMP received consuting fees from Abbvie, Lilly and Janssen. RMX received Speaker fees, consulting, research funding from Abbvie, Pfizer, Amgen, Biogen, Novartis, Janssen, UCB. RR is/has served as a scientific consultant, speaker, or clinical study investigator for AbbVie, Boehringer Ingelheim, Bristol Myers Squibb, Galderma, Janssen-Cilag, Eli-Lilly, Leo-Pharma, Novartis, Pfizer, Sanofi, and UCB. RSH received speaker fees and consulting from Abbvie, Novartis, Takeda, Johnson & Johnson. OAM received consulting fees from AstraZeneca and GSK; received payment or honoraria for lectures or presentations from AbbVie, Janssen, GSK, UCB and AstraZeneca; and participated in a data safety monitoring board or advisory board for GSK, CellTrion, Bristol, Novartis, Janssen, and AstraZeneca. VAS received speaker fees and consulting from Abbvie, Novartis, Lilly, Johnson & Johnson, GSK, Astrazeneca. VFA received speaker fees, consulting, clinical researches from Celltiron, Amgen, Janssen, Organon, Sandoz, Abbvie, Fresenius Kabi, Takeda, LG, Boehringer-Ingelheim, Bristol Myers-Squibb, GSK, AstraZeneca. Eduardo dos Santos Paiva is Co-EiC of Advances in Rheumatology. Ana Karla Guedes de Melo, Ana Cristina de Medeiros Ribeiro and Marcelo de Medeiros Pinheiro are Associate Editors of the journal Advances in Rheumatology; Ricardo Machado Xavier is a Consulting editor of the journal; Adriana Maria Kakehasi and Licia Maria Henrique da Mota are members of the editorial board of this journal. The peer-review of this paper was therefore overseen by an independent member of the editorial board while the submission was subject to the exact same review process as applied to any other manuscript. The authors not mentioned above declare no competing interests.

