# Supplementary material for: Brazilian recommendations for the management of tuberculosis infection in immune-mediated inflammatory diseases
Source: Adv Rheumatol. 2025 Mar 20;65:18. doi: 10.1186/s42358-025-00449-4 (PMC13488801; doi:10.1186/s42358-025-00449-4)
Supplement: Supplementary file 1 [file 42358_2025_449_MOESM1_ESM.docx]

# **Brazilian recommendations for the management of tuberculosis infection in immune-mediated inflammatory diseases - Supplementary material**

**Appendix 2**

Table 1 – Main characteristics of the included studies with risk of bias assessment

| AUTHORS | YEAR OF PUBLICATION | STUDY DESIGN | TIME OF DATA COLECTION | GEOGRAPHIC LOCATION | IMIDs | DMARDs | RISK OF BIAS |
| --- | --- | --- | --- | --- | --- | --- | --- |
| Aachari I^1^ | 2020 | Cross sectional | NP | Moroccan | AS | TNFi | VERY HIGH RISK |
| Abdala B ^2^ | 2021 | Cohort | 2018 to 2019 | Argentina | RA, JIA,AS, PsA, uveitis, interstitial keratitis | ADA,CZP, ETA, MTX, LFN, HCQ, SSA, AZA, MMF, steroids | LOW RISK |
| Aberra FN^3^ | 2009 | Cohort | NP | Switzerland | IBD | Immunomodulators, steroids, immunosuppressants, IFX, tacrolimus | LOW RISK |
| Abreu C^4^ | 2013 | Case Control | 2001 to 2012 | Portugal | IMID | TNFi | VERY HIGH RISK |
| Abreu C^5^ | 2016 | Cohort | 2012 to 2015 | Portugal | IBD | TNFi | VERY HIGH RISK |
| Abreu C^6^ | 2017 | Cohort | 2013 | Portugal | IBD | IFX | VERY HIGH RISK |
| Acar M^7^ | 2017 | Cohort | 2011 to 2016 | Turkey | IMID | TNFi, AZA, steroids, MTX, cyclosporine | LOW RISK |
| Agarwal A^8^ | 2018 | Cohort | 2005 to 2017 | India | IBD | IFX | HIGH RISK |
| Aggarwal R^9^ | 2009 | Cohort | 2001 to 2008 | USA | IMID | ETN | LOW RISK |
| Ahn GY^10^ | 2020 | Cohort | 1998 to 2019 | Korea | AS | TNFi | LOW RISK |
| Akici A^11^ | 2017 | Cohort | 2013 to 2015 | Turkey | RA, AS, JIA, PsA | ETN, ADA, IFX, GOLI, CZP | HIGH RISK |
| Akyuz F^12^ | 2019 | Cohort | 2000 to 2014 | Turkey | IBD | TNFi | LOW RISK |
| Al Hajoj S^13^ | 2016 | Cross sectional | 2012 to 2015 | Saudi Arabia | Healthy subjects | NP | LOW RISK |
| Al-Sohaim A^14^ | 2021 | Cohort | 2015 to 2019 | Saudi Arabia | IMID | ADA | LOW RISK |
| Al-Taweel T^15^ | 2018 | Cross sectional | 2010 to 2014 | Canada | IBD | Steroids, thiopurines, MTX | LOW RISK |
| Al-Taweel T^15^ | 2014 | Cross sectional | NP | Kwait | IBD | TNFi | LOW RISK |
| Alaşan F^16^ | 2020 | Cross sectional | 2008 to 2015 |  | RA, AS, PSO, PSA | MTX, SSA, steroids, IFX, ETA, ADA, GOLI | LOW RISK |
| Algaba A^17^ | 2018 | Cohort | 2012 to 2017 | Spain | IBD | IFX, ADA, GOLI | LOW RISK |
| Alkadi A^18^ | 2017 | Cohort | 2010 to 2011 | Saudi Arabia | RA, SLE, DM, overlap syndrome, JIA, polymyositis, GPA, MCTD, SS | RITUXI | HIGH RISK |
| Amorim RF^19^ | 2018 | Cross sectional | NP | Brazil | IBD | TNFi | LOW RISK |
| Andrisani G^20^ | 2013 | Cross sectional | 2008 to 2010 | Italy | IBD | AZA, MTX, steroids | LOW RISK |

| AUTHORS | YEAR OF PUBLICATION | STUDY DESIGN | TIME OF DATA COLECTION | GEOGRAPHIC LOCATION | IMIDs | DMARDs | RISK OF BIAS |
| --- | --- | --- | --- | --- | --- | --- | --- |
| Anzueto E^21^ | 2019 | Cohort | NP | Multicentric | RA | TOFA | HIGH RISK |
| Archimandriti DT^22^ | 2017 | Cross sectional | NP | Greece | RA | Steroids, csDMARDs, bDMARDs | LOW RISK |
| Argüder E^23^ | 2020 | Cross sectional | 2015 to 2019 | Turkey | AS, RA, PsA, SLE, Familial Mediterranean fever, Behcet, Sjögren | ETA, ADA, GOLI, CZP, TOFA, TOCI, SECUKI, RITUXI, ABATA, canakinumab, anakinra | LOW RISK |
| Arias M^24^ | 2011 | Cross sectional | NP | Spain | IBD | Steroids, TNFi, immunomodulators | LOW RISK |
| Askling J^25^ | 2005 | Cohort | 1999 to 2001 | Sweden | RA | TNFi | HIGH RISK |
| Atteno M^26^ | 2014 | Cohort | 2005 to 2011 | Italy | PSO | TNFi | LOW RISK |
| Aydin V^27^ | 2019 | Cohort | 2013 to 2015 | Turkey | RA, AS, JIA, PsA | ETN, IFX, ADA | HIGH RISK |
| Aydin V^28^ | 2017 | Cohort | 2013 | Turkey | RA, AS, JIA, PsA | ETN, IFX, ADA | HIGH RISK |
| Bachelez H^29^ | 2019 | RCT | NP | France | PSO | Risankizumab | LOW RISK |
| Baddley JW^30^ | 2014 | Cohort | 1998 to 2007 | USA | RA, IBD, PsA, AS, PSO | TNFi | HIGH RISK |
| Baldassano R^31^ | 2013 | Cross sectional | 2011 | USA | IBD | ADA | VERY HIGH RISK |
| Barosa R^32^ | 2016 | Cross sectional | 2001 to 2015 | Portugal | IBD | TNFi | LOW RISK |
| Bartalesi F^33^ | 2009 | Cohort | 2006 to 2007 | Italy | RA, PsA, AS, other IMID | csDMARDs, steroids, TNFi | LOW RISK |
| Barut K^34^ | 2018 | Cohort | NP | Turkey | JIA | Steroids, MTX, TNFi | LOW RISK |
| Basyal RK^35^ | 2014 | Cross sectional | NP | India | RA, SpA | IFX | LOW RISK |
| Bautista Molano W^36^ | 2021 | Cross sectional | NP | Colombia | AR | ETA, IFX, ABATA, CZP, ADA, RITUXI, TOCI, csDMARDs | LOW RISK |
| Benhadou F^37^ | 2021 | Cohort | NP | Belgium | PSO | ADA, ETN, MTX | LOW RISK |
| Bensen W^38^ | 2013 | Cross sectional | 2008 to 2012 | Canada | RA | ABATA | VERY HIGH RISK |
| Bensen W^39^ | 2015 | Cohort | 2010 to 2014 | Canada | RA | GOLI | HIGH RISK |
| Benzaquén N^40^ | 2016 | Cohort | 2016 | Argentina | RA, PsA, JIA, SLE | ETN, ADA, ABATA | HIGH RISK |
| Bermejo F^41^ | 2013 | Cross sectional | NP | Spain | IBD | TNFi, MTX, thiopurines, aminosalicylates | LOW RISK |
| Beshir MR^42^ | 2016 | Cross sectional | 2014 to 2015 | Egypt | Healthy subjects | NP | LOW RISK |
| Bierrenbach AL^43^ | 2003 | Cross sectional | 2003 | Brazil | Healthy subjects | NP | LOW RISK |
| Blauvelt A^44^ | 2019 | RCT | NP | USA | PSO | CZP | LOW RISK |
| Bonfiglioli KR^45^ | 2014 | Cohort |  |  | RA | TNFi | LOW RISK |
| Borekci S^46^ | 2015 | Cohort | 2003 to 2013 | Turkey | AS, AR, Behçet, other IMID | ETN, IFX, or ADA | LOW RISK |

| AUTHORS | YEAR OF PUBLICATION | STUDY DESIGN | TIME OF DATA COLECTION | GEOGRAPHIC LOCATION | IMIDs | DMARDs | RISK OF BIAS |
| --- | --- | --- | --- | --- | --- | --- | --- |
| Bowerman RJ^47^ | 2004 | Cross sectional | NP | Taiwan | Healthy subjects | NP | LOW RISK |
| Bozaykut A^48^ | 2004 | Cohort | NP | Turkey | Healthy subjects | NP | LOW RISK |
| Brassard P^49^ | 2009 | Case Control | 1992 to 2003 | Canada | RA | MTX, LFN, CsA, other csDMARDs | VERY HIGH RISK |
| Brunelli JB^50^ | 2017 | Cohort | 2007 to 2015 | Brazil | JIA | TNFi | LOW RISK |
| Bruzzese V^51^ | 2013 | RCT | NP | Italy | RA, AS, PsA, PSO, IBD | IFX, ADA, CZP | HIGH RISK |
| Bugiani M^52^ | 2003 | Cohort | NP | China | Healthy subjects | NP | LOW RISK |
| Burmester GR^53^ | 2017 | RCT | NP | Multicentric | RA | ADA | LOW RISK |
| Byun JM^54^ | 2015 | Cohort | 2001 to 2013 | Korea | IBD | TNFi | HIGH RISK |
| Cabriada JL^55^ | 2018 | Cross sectional | NP | Spain | IBD | steroids, csDMARDs, TNFi | LOW RISK |
| Cadavid M^56^ | 2016 | Cross sectional | NP | Colombia | IMID | bDMARDs | LOW RISK |
| Cagatay T^57^ | 2018 | Cohort | 2005 to 2015 | Turkey | AS, RA, PsA, IBD, Behçet, JIA, other | TNFi | VERY HIGH RISK |
| Cagatay T^58^ | 2011 | Cohort | NP | Turkey | RA, AS, PsA, other IMID | TNFi | VERY HIGH RISK |
| Callado MRM^59^ | 2011 | Case Control | 2005 to 2008 | Brazil | RA, AS, PsA | IFX | LOW RISK |
| Calzada-Hernández J^60^ | 2015 | Cross sectional | NP | Spain | JIA, PSO, IBD | steroids, DMARDs | LOW RISK |
| Calzada-Hernández J^61^ | 2015 | Cross sectional | 2004 to 2013 | Spain | JIA, Blau syndrome, Tumor necrosis factor associated periodic syndrome, PAPA syndrome, Chronic plantar fascitis, IBD, inflammatory diseases of the eye | ETN, IFX, ADA, MTX | LOW RISK |
| Çamlar SA^62^ | 2011 | Cohort | NP | Turkey | JIA | NP | LOW RISK |
| Campanilho-Marques R^63^ | 2016 | Cross sectional | NP | UK | juvenile DM | TNFi | LOW RISK |
| Carvalho L^64^ | 2017 | Cross sectional | 2000 to 2016 | Portugal | IBD | TNFi | LOW RISK |
| Castellani C^65^ | 2021 | Cohort | NP | Italy | RA | JAKi | LOW RISK |
| Çekiç C^66^ | 2015 | Cohort | 2007 to 2014 | Turkey | IBD | IFX, ADA | HIGH RISK |
| Cerda OL^67^ | 2014 | Cross sectional | NP | Argentina | RA, JIA, PsA | TNFi, TOCI, ABATA | LOW RISK |
| Cerda OL^68^ | 2018 | Cross sectional | NP | Argentina | RA, JIA, PsA, axial SpA | ETA, ADA, IFX, CZP, GOLI, TOCI, ABATA | LOW RISK |

| AUTHORS | YEAR OF PUBLICATION | STUDY DESIGN | TIME OF DATA COLECTION | GEOGRAPHIC LOCATION | IMIDs | DMARDs | RISK OF BIAS |
| --- | --- | --- | --- | --- | --- | --- | --- |
| Chaer FGG^69^ | 2020 | Cohort | 2013 to 2017 | Brazil | RA, AS, JIA, PsA | ADA, IFX, ETN, TOCI | VERY HIGH RISK |
| Chan MJ^70^ | 2018 | Cohort | 2005 to 2013 | China | RA, SpA | TNFi, csDMARD | HIGH RISK |
| Chandra A^71^ | 2019 | Cohort | 2017 to 2019 | India | IBD | ADA | HIGH RISK |
| Chang B^72^ | 2010 | Cross sectional | NP | Korea | AS, RA, other IMID | ADA, ETA, IFX, other TNFi | LOW RISK |
| Chang HC^73^ | 2010 | Cohort | 2006 to 2009 | Taiwan | RA | ETN, ADA | HIGH RISK |
| Cheah C^74^ | 2015 | Cross sectional | NP | Malaysia | IMID | bDMARD | LOW RISK |
| Chen DY^75^ | 2016 | Cohort | NP | Taiwan | RA | ADA, ETA, GOLI, ABATA, TOCI, MTX | LOW RISK |
| Chen HH^76^ | 2016 | Cohort | 2003 to 2012 | Taiwan | RA | TNFi | HIGH RISK |
| Chen YJ^77^ | 2013 | Cohort | 1996 to 2008 | China | PSO, PsA | MTX, CsA, AZA, steroids | HIGH RISK |
| Chiacchio T^78^ | 2018 | Cross sectional | NP | Italy | RA, other IMID | NP | LOW RISK |
| Chiu HY^79^ | 2011 | Cross sectional | 2004 to 2009 | Taiwan | PSO | ETA, ADA | LOW RISK |
| Chiu YM^80^ | 2014 | Cohort | 1999 to 2009 | China | RA | tsDMARD, bDMARD | HIGH RISK |
| Cho H^81^ | 2016 | Cohort | 2013 to 2015 | Korea | SLE, RA | Steroids, HCQ, MTX, AZA, LFN, MMF, tacrolimus, cyclophosphamide | LOW RISK |
| Cho SI^82^ | 2020 | Cohort | 2012 to 2018 | Korea | PSO, PsA | USTEKI | HIGH RISK |
| Chung TT^83^ | 2020 | Cohort | 1994 to 2019 | China | RA | SSA, MTX, LFN, ETN, IFX, ADA, GOLI, CZP, TOCI, ABATA, RITUXI, TOFA | HIGH RISK |
| Ciang NC^84^ | 2020 | Cohort | 1994 to 2019 | China | SpA | SSA, MTX, LFN, ETN, IFX, ADA, GOLI, CZP, SECUKI, USTEKI, steroids | HIGH RISK |
| Cohen S^85^ | 2018 | Cohort | 2012 to 2015 | Multicentric | RA | TOFA | HIGH RISK |
| Conti A^86^ | 2017 | Cohort | 2004 to 2012 | Italy | PSO | IFX, ADA, ETN, efalizumab, USTEKI | LOW RISK |
| Coskunol I^87^ | 2015 | Cross sectional | 2011 to 2014 | Turkey | AS, RA, IBD, PsA, PSO | TNFi | LOW RISK |
| Costantino F^88^ | 2013 | Cohort | 2005 to 2009 | France | RA, SpA | TNFi | LOW RISK |
| Costantino F^89^ | 2010 | Cross sectional | 2004 to 2009 | France | RA, SpA, other IMID | TNFi, csDMARD, steroids | LOW RISK |
| Cuomo G^90^ | 2017 | Cohort | 2005 to 2015 | Italy | RA | TNFi, ABATA, TOCI | LOW RISK |
| Dashdamirova S^91^ | 2019 | Cross sectional | 1998 to 2018 | Turkey | IBD | IFX, ADA, CZP, GOLI, vedolizumab | LOW RISK |
| Davì S^92^ | 2015 | Cohort | NP | Italy | JIA | ETN | HIGH RISK |
| De Castro ML^93^ | 2014 | Cross sectional | 2000 to 2012 | Spain | IBD | TNFi | LOW RISK |

| AUTHORS | YEAR OF PUBLICATION | STUDY DESIGN | TIME OF DATA COLECTION | GEOGRAPHIC LOCATION | IMIDs | DMARDs | RISK OF BIAS |
| --- | --- | --- | --- | --- | --- | --- | --- |
| de Francisco R^94^ | 2011 | Cross sectional | NP | Spain | IBD | AZA, MTX, TNFi, steroids | LOW RISK |
| de Francisco R^95^ | 2019 | Cross sectional | 2009 to 2018 | Spain | IBD | bDMARDs | LOW RISK |
| De Leon DP^96^ | 2010 | Cohort | NP | Peru | RA | Np | LOW RISK |
| de Medeiros VLS^97^ | 2020 | Cohort | 2015 to 2018 | Brazil | PSO | MTX | LOW RISK |
| Del Tedesco E^98^ | 2010 | Cross sectional | NP | Multicentric | IBD | TNFi | LOW RISK |
| Dixon WG^99^ | 2010 | Cohort | 2001 to 2006 | UK | RA | ETN, IFX, ADA | HIGH RISK |
| Dogan C^100^ | 2011 | Cross sectional | 2007 to 2010 | NP | RA, AS, JIA, PsA | TNFi | VERY HIGH RISK |
| Eddarami J^101^ | 2018 | Cross sectional | NP | Morocco | SpA | TNFi, MTX, SSA, steroids | LOW RISK |
| Edet A^102^ | 2018 | Cohort | 2007 to 2010 | UK | Healthy subjects | NP | LOW RISK |
| Ehrenstein B^103^ | 2009 | Cross sectional | 2008 | Germany | RA, SpA, PsA, other IMID | steroids, bDMARDs | LOW RISK |
| Elbek O^104^ | 2009 | Cohort | 2005 to 2007 | Turkey | IMID | TNFi | LOW RISK |
| Escalante P^105^ | 2014 | Cross sectional | 2007 | USA | RA, AS, SLE, other IMID | TNFi, csDMARD, steroids | LOW RISK |
| Fajardo Hermosillo LD^106^ | 2016 | Cohort | 2011 to 2016 | Mexico | AS, JIA, PsA | TNFi, steroids | LOW RISK |
| Favalli EG^107^ | 2009 | Cohort | NP | Italy | RA | IFX, ETN, ADA | HIGH RISK |
| Ferrante M^108^ | 2018 | Cross sectional | NP | Belgium | IBD | Risankizumab | VERY HIGH RISK |
| Fildan AP^109^ | 2015 | Cross sectional | NP | Romania | AS, PsA, RA | TNFi | LOW RISK |
| Foeldvari I^110^ | 2017 | Cross sectional | NP | Italy | JIA, PsA | ETN | VERY HIGH RISK |
| Fortes FML^111^ | 2020 | Cohort | 2017 to 2018 | Brazil | IBD | TNFi, AZA, MTX | HIGH RISK |
| Foster RA^112^ | 2011 | Cross sectional | NP | UK | IBD, PSO, inflammatory arthropathies, other IMID | TNFi, immunomodulators | LOW RISK |
| Frolova X^113^ | 2020 | Cross sectional | 2009 to 2018 | Russia | IBD | TNFi | LOW RISK |
| Fuchs I^114^ | 2009 | Cross sectional | NP | Israel | RA, AS, PsA | TNFi | LOW RISK |
| Fuentes Coronel AM^115^ | 2017 | Cohort | 2015 to 2017 | Spain | IBD | NP | LOW RISK |
| Fujikawa A^116^ | 2014 | Cross sectional | 2009 to 2010 | Japan | Healthy subjects | NP | LOW RISK |
| Gabriele F^117^ | 2017 | Cross sectional | 2011 to 2012 | Greece | JIA, SLE | csDMARDs, bDMARDs, steroids | LOW RISK |
| Gao Y^118^ | 2011 | Case Control | NP | China | IMIDs | Immunosuppressive therapy | LOW RISK |
| García-Gasalla M^119^ | 2013 | Cohort | 2008 to 2010 | Spain | IBD, RA, PSO, PsA, AS, other IMID | steroids, csDMARDs, TNFi | LOW RISK |
| García-Sancho F^120^ | 2006 | Cross sectional | 2000 to 2001 | Mexico | Healthy subjects | NP | LOW RISK |

| AUTHORS | YEAR OF PUBLICATION | STUDY DESIGN | TIME OF DATA COLECTION | GEOGRAPHIC LOCATION | IMIDs | DMARDs | RISK OF BIAS |
| --- | --- | --- | --- | --- | --- | --- | --- |
| Garcovich S^121^ | 2012 | Cohort | 2008 to 2010 | Italy | PSO | TNFi | LOW RISK |
| Garziera G^122^ | 2017 | Cohort | 2007 to 2015 | Brazil | RA, AS, PsA, JIA, SpA, Behçet disease, GPA | IFX, ADA, ETN, GOLI | VERY HIGH RISK |
| Gazel U^123^ | 2019 | Cohort | 2005 to 2018 | Turkey | Behçet | ETN, ADA, IFX, CZP | HIGH RISK |
| Gheorghe C^124^ | 2019 | Cross sectional | 2017 to 2018 | Romania | IBD | TNFi | LOW RISK |
| Gİrİt S^125^ | 2019 | Cohort | 2015 to 2017 | Turkey | JIA, SS, SpA, SLE | MTX, AZA, SSA, TNFi, tacrolimus, colchicine | LOW RISK |
| Gîrleanu I^126^ | 2013 | Cross sectional | NP | Romania | IBD | TNFi | LOW RISK |
| Gisondi P^127^ | 2014 | Cohort | 2013 | Italy | PSO | ETN, ADA, IFX or USTEKI | LOW RISK |
| Gogna A^128^ | 2018 | Cross sectional | NP | India | IMID | IFX, ADA, GOLI, ETN, RITUXI | LOW RISK |
| Gomes CMF^129^ | 2015 | Cohort | NP | Brazil | IMID | TNFi | LOW RISK |
| Gomes CMF^130^ | 2021 | Cohort | NP | Brazil | RA, As, PsA | Steroids, MTX, LFN, SSA, CsA, ADA, ETN, IFX | LOW RISK |
| Gómez-Reino JJ^131^ | 2003 | Cohort | 1999 to 2002 | Spain | RA | IFX, ETN | HIGH RISK |
| González-Díaz V^132^ | 2013 | Cross sectional | NP | Mexico | SLE | Immunosuppressive therapy | LOW RISK |
| Greveson K^133^ | 2012 | Cohort | 2010 to 2011 | UK | IBD | TNFi | LOW RISK |
| Greveson K^134^ | 2010 | Cross sectional | 2008 | UK | IBD | TNFi | LOW RISK |
| Gudjónsdóttir MJ^135^ | 2016 | Cohort | 2007 to 2016 | Sweden | Healthy subjects | NP | LOW RISK |
| Guidi L^136^ | 2010 | Cross sectional | 2008 to 2009 | Italy | IBD | TNFi | LOW RISK |
| Hacioglu A^137^ | 2022 | Cohort | 2010 to 2012 | Turkey | AS, RA, Behçet, PsA, IBD | TNFi | LOW RISK |
| Hakimian S^138^ | 2018 | Cross sectional | 2008 to 2016 | USA | IBD, RA | TNFi | LOW RISK |
| Hanta I^139^ | 2008 | Cohort | 2005 to 2008 | Turkey | IMID | TNFi | LOW RISK |
| Hanta I^140^ | 2012 | Cross sectional | NP | Turkey | RA, AS, PsA | csDMARD | LOW RISK |
| Hanta I^141^ | 2011 | Cross sectional | NP | Turkey | AS | TNFi | LOW RISK |
| Hatzara C^142^ | 2015 | Cross sectional | NP | Greece | RA, SpA, other IMID | ADA, ETA, IFX, GOLI, CZP | LOW RISK |
| Havlucu Y^143^ | 2015 | Cross sectional | NP | NP | IMID | TNFi | VERY HIGH RISK |
| He D^144^ | 2013 | Cohort | 2008 to 2009 | China | IMID | bDMARD | LOW RISK |
| Hejazi ME^145^ | 2020 | Cohort | 2017 to 2019 | Iran | AS, RA | IFX, HCQ, MTX, steroids, other bDMARD | LOW RISK |

| AUTHORS | YEAR OF PUBLICATION | STUDY DESIGN | TIME OF DATA COLECTION | GEOGRAPHIC LOCATION | IMIDs | DMARDs | RISK OF BIAS |
| --- | --- | --- | --- | --- | --- | --- | --- |
| Henriques A^146^ | 2019 | Cross sectional | NP | Portugal | IBD | TNFi | LOW RISK |
| Hernández MV^147^ | 2012 | Cohort | 2000 to 2011 | Spain | RA, AS, PsA, JIA, SpA, Behçet | IFX, ETN, ADA | HIGH RISK |
| Hizel K^148^ | 2004 | Cross sectional | 2000 | Turkey | Healthy subjects | NP | LOW RISK |
| Hofland RW^149^ | 2013 | Cross sectional | NP | Netherlands | IBD, AS | TNFi | VERY HIGH RISK |
| Hong S^150^ | 2014 | Cohort | NP | Korea | AS, RA, PsA, Behçet, other | ETN, ADA, IFX | HIGH RISK |
| Hong SN^151^ | 2017 | Cohort | 2011 to 2013 | Korea | IBD | steroids, immunomodulator, TNFi | HIGH RISK |
| Horowitz HW^152^ | 1995 | Cohort | 1991 to 1993 | USA | Healthy subjects | NP | LOW RISK |
| Hou JK^153^ | 2017 | Cohort | 2003 to 2011 | USA | IBD | TNFi | LOW RISK |
| Hradsky O^154^ | 2014 | Cross sectional | NP | Czech Republic | IBD | Steroids, immunomodulators | LOW RISK |
| Hsia EC^155^ | 2012 | Cohort | NP | Multicentric | RA, PSA, AS | GOLI | LOW RISK |
| Hsia EC^156^ | 2013 | RCT | NP | Multicentric | RA | GOLI | LOW RISK |
| Hsiao CY^157^ | 2017 | Cohort | 2010 to 2016 | Taiwan | PSO | USTEKI | LOW RISK |
| Hsieh TY^158^ | 2018 | Cohort | 2013 to 2018 | Taiwan | PSO | USTEKI | LOW RISK |
| Huang SF^159^ | 2018 | Cohort | 2012 to 2014 | Taiwan | IMID | bDMARD | LOW RISK |
| Husni E^160^ | 2018 | RCT | NP | USA | PsA | GOLI | HIGH RISK |
| Iba A^161^ | 2020 | Cross sectional | 2012 to 2019 | Japan | RA, IBD, PSO, other IMID | IFX, ETN, ADA, GOLI, CZP, ABATA, TOCI, USTEKI, SECUKI | LOW RISK |
| Igari H^162^ | 2017 | Cross sectional | NP | Japan | RA | NP | LOW RISK |
| Igari H^163^ | 2015 | Cross sectional | NP | Japan | RA | Steroids, MTX, bDAMRDs | LOW RISK |
| Ines M^164^ | 2018 | Cross sectional | 2014 to 2016 | Tunisia | Healthy subjects | NP | LOW RISK |
| Jacobs S^165^ | 2011 | Cross sectional | NP | Canada | Healthy subjects | NP | LOW RISK |
| Jauregui-Amezaga A^166^ | 2013 | Cohort | 2000 to 2011 | Spain | IBD | IFX, ADA | HIGH RISK |
| Jeong DH^167^ | 2018 | Cohort | 2011 to 2014 | Korea | RA, AS | TNFi, MTX, steroids, other csDMARD | LOW RISK |
| Jo KW^168^ | 2013 | Cross sectional | 2004 to 2012 | Korea | AS, RA, IBD, PSO | Steroids, DMARDs | LOW RISK |
| Joza K^169^ | 2020 | Cross sectional | 2016 to 2017 | Buenos Aires | Healthy subjects | NP | LOW RISK |
| Julian AN^170^ | 2014 | Cross sectional | NP | Spain | IMID | TNFi | LOW RISK |

| AUTHORS | YEAR OF PUBLICATION | STUDY DESIGN | TIME OF DATA COLECTION | GEOGRAPHIC LOCATION | IMIDs | DMARDs | RISK OF BIAS |
| --- | --- | --- | --- | --- | --- | --- | --- |
| Jung HJ^171^ | 2014 | Cross sectional | 2009 to 2012 | Korea | RA, AS, SLE, Behçet, adult-onset Still’s disease | Steroids, MTX, SSA, HCQ, LFN, CsA, TNFi, tacrolimus | LOW RISK |
| Jung SM^172^ | 2015 | Cohort | 2005 to 2009 | Korea | RA, AS, PsA, IBD | ETN, IFX, ADA | HIGH RISK |
| Jung YJ^173^ | 2014 | Cohort | 2008 to 2012 | Korea | IMID | TNFi | LOW RISK |
| Jung YJ^174^ | 2015 | Cross sectional | 2009 to 2012 | Korea | RA, AS, other IMID | ETA, ADA, IFX, MTX, tacrolimus, steroids | LOW RISK |
| Kamat N^175^ | 2018 | Cohort | 2015 to 2018 | NP | IBD | ADA | HIGH RISK |
| Kaneko S^176^ | 2020 | Cross sectional | 2010 to 2017 | Japan | PsA | DMARDs | VERY HIGH RISK |
| Kang J^177^ | 2018 | Cohort | 2011 to 2017 | Korea | IBD | IFX, ADA, GOLI | HIGH RISK |
| Kaptan Y^178^ | 2021 | Cohort | 2010 to 2016 | Turkey | AS, PsA, AR, other IMID | TNFi | LOW RISK |
| Karataş Toğral A^179^ | 2016 | Cross sectional | 2011 | Turkey | PSO | NP | LOW RISK |
| Kaur M^180^ | 2018 | Case Control | 2011 to 2013 | USA | IBD | Steroids, other DMARDs | LOW RISK |
| Kay J^181^ | 2009 | RCT | 2008 | NP | RA, PsA, AS | GOLI | HIGH RISK |
| Ke WM^182^ | 2013 | Cohort | 2006 to 2008 | China | RA | TNFi | LOW RISK |
| Khraishi M^183^ | 2012 | Cross sectional | NP | Germany and Canada | RA | ABATA | VERY HIGH RISK |
| Kilinc AA^184^ | 2020 | Cohort | NP | Turkey | IMID | TNFi | LOW RISK |
| Kim EM^185^ | 2011 | Cohort | 2002 to 2009 | Korea | AS | ADA, IFX, ETN | HIGH RISK |
| Kim EM^186^ | 2010 | Cohort | 2002 to 2009 | Korea | AS | ADA, IFX, ETN | HIGH RISK |
| Kim ES^187^ | 2015 | Cohort | 2003 to 2014 | Korea | IBD | IFX, ADA | HIGH RISK |
| Kim HC^188^ | 2014 | Cohort | 2010 to 2013 | Korea | IMIDs | TNFi | LOW RISK |
| Kim HW^189^ | 2014 | Cohort | 2001 to 2011 | Korea | AS, RA | ETN, ADA, IFX, GOLI, CZP | HIGH RISK |
| Kim HW^190^ | 2020 | Cohort | NP | Korea | RA, AS | ETN, TOCI, ABATA, other TNFi | LOW RISK |
| Kim J^191^ | 2020 | Case Control | 2001 to 2003 | Korea | IBD | IFX, ADA | VERY HIGH RISK |
| Kim JH^192^ | 2015 | Cohort | 2007 to 2012 | Korea | IMIDs | TNFi | LOW RISK |
| Kim JH^193^ | 2013 | Cross sectional | 2008 to 2010 | Korea | RA, AS, JIA | Steroids, MTX, TNFi | LOW RISK |
| Kim KH^194^ | 2011 | Cohort | 2006 to 2010 | Korea | AS, SLE, RA, JIA, Behçet, adult-onset Still’s disease, antiphospholipid syndrome | TNFi, MTX, steroids, CsA, other DMARDs | LOW RISK |
| Kimball AB^195^ | 2014 | Cohort | NP | USA | PSO | ETN | LOW RISK |
| Kimball AB^196^ | 2012 | RCT | NP | Multicentric | PSO | USTEKI | LOW RISK |
| Kiwanuka JP^197^ | 2005 | Cross sectional | NP | Uganda | Healthy subjects | NP | LOW RISK |

| AUTHORS | YEAR OF PUBLICATION | STUDY DESIGN | TIME OF DATA COLECTION | GEOGRAPHIC LOCATION | IMIDs | DMARDs | RISK OF BIAS |
| --- | --- | --- | --- | --- | --- | --- | --- |
| Kleinert S^198^ | 2012 | Cohort | NP | Germany | RA, AS, PsA, SpA, other IMID | NP | LOW RISK |
| Koike T^199^ | 2010 | Cohort | NP | Japan | RA | TOCI | HIGH RISK |
| Koo BS^200^ | 2021 | Cohort | 2012 to 2017 | Korea | AS | ETN, ADA, GOLI, IFX | HIGH RISK |
| Koo BS^201^ | 2013 | Cross sectional | 2003 to 2010 | Korea | AS, RA, PsA, SpA, Behçet | TNFi | HIGH RISK |
| Kruczak K^202^ | 2010 | Case Control | 2009 | Poland | RA, PSO | Steroids | LOW RISK |
| Kumar P^203^ | 2022 | Cohort | 2005 to 2019 | India | IBD | TNFi | LOW RISK |
| Kurt OK^204^ | 2013 | Cross sectional | 2006 to 2012 | Turkey | AS, RA, JIA, PsA | ETA, IFX, ADA | LOW RISK |
| Kurtz T^205^ | 2019 | Cohort | NP | BRAZIL | Healthy subjects | NP | LOW RISK |
| Kwon M^206^ | 2014 | Cohort | 2005 to 2012 | Korea | IMID | ETN, IFX, or ADA | LOW RISK |
| Lai H-C^207^ | 2019 | Cross sectional | 2000 to 2018 | Taiwan | IBD | Steroids, salicylates, ADA, IFX, vedolizumab, GOLI | LOW RISK |
| Laurindo I^208^ | 2012 | Cohort | 2007 to 2011 | Brazil | RA | TNFi | LOW RISK |
| Lawrance IC^209^ | 2009 | Cohort | NP | Australia and New Zealand | IBD | TNFi | HIGH RISK |
| Lee CK^210^ | 2018 | Cohort | 2012 to 2017 | China | IMID | TNFi, USTEKI, TOCI | LOW RISK |
| Lee EH^211^ | 2017 | Cohort | 2005 to 2016 | Korea | RA, AS, IBD | IFX, ADA, CZP, GOLI, ETA, USTEKI, TOCI | LOW RISK |
| Lee H^212^ | 2015 | Cohort | 2007 to 2013 | Korea | RA, AS | Steroids, MTX, LFN, TNFi | LOW RISK |
| Lee HW^213^ | 2017 | Cohort | 2011 to 2014 | Korea | Healthy subjects | NP | LOW RISK |
| Lee J^214^ | 2017 | Cohort | 2011 to 2013 | Korea | IMID | TNFi | LOW RISK |
| Lee J^215^ | 2019 | Cross sectional | 2008 to 2017 | Korea | RA | TNFi | LOW RISK |
| Lee JY^216^ | 2021 | Cohort | 2001 to 2018 | Korea | IBD | TNFi | LOW RISK |
| Lee SJ^217^ | 2019 | Cohort | NP | Portugal and Korea | IBD | IFX | HIGH RISK |
| Lee SW^218^ | 2014 | Cohort | 2005 to 2011 | Korea | Healthy subjects | NP | LOW RISK |
| Li H^219^ | 2017 | Cross sectional | 2013 | China | Healthy subjects | NP | LOW RISK |
| Liao TL^220^ | 2016 | Cohort | 2001 to 2011 | Italy | RA | cDMARDs, ETN, ADA, RITUXI | HIGH RISK |
| Lim CH^221^ | 2017 | Cohort | 2000 to 2015 | China | RA | ETN, ADA, ABATA, GOLI, TOCI, TOFA | HIGH RISK |
| Lim CH^222^ | 2016 | Cohort | 2003 to 2013 | China | RA | ETN | HIGH RISK |
| Lima C^223^ | 2019 | Cohort | 2010 to 2017 | Brazil | IBD | TNFi (IFX, ADA), AZA, steroids | HIGH RISK |
| Lima EVA^224^ | 2010 | Cohort | 2009 | Brazil | PSO | NP | LOW RISK |
| Logarajah V^225^ | 2019 | Cross sectional | 2008 to 2019 | Singapore | IBD | TNFi | LOW RISK |

| AUTHORS | YEAR OF PUBLICATION | STUDY DESIGN | TIME OF DATA COLECTION | GEOGRAPHIC LOCATION | IMIDs | DMARDs | RISK OF BIAS |
| --- | --- | --- | --- | --- | --- | --- | --- |
| Long W^226^ | 2020 | Cohort | 2012 to 2016 | China | SLE, other | steroids, AZA, CsA, Cyclophosphamide, Tacrolimus, HCQ, MMF, MTX, Thalidomide | HIGH RISK |
| Lopes DMA^227^ | 2011 | Cohort | 2008 to 2009 | Brazil | IMID | MTX, immunomodulators, steroids | LOW RISK |
| Lopes DMA^228^ | 2019 | Cohort | 2001 to 2015 | Brazil | IMIDs | IFX, ETN, ADA, GOLI, RITUXI, TOCI, ABATA, USTEKI, bevacizumab | LOW RISK |
| Lorenzetti R^229^ | 2013 | RCT | NP | Multicentric | IBD, PSO, other | IFX, ADA, CZP | L71037328 |
| Losi M^230^ | 2010 | Cross sectional | NP | Italy | RA, PsA, AS, undifferentiated spondyloarthropathy, other IMID | Steroids, DMARDs | LOW RISK |
| Lupaşcu A^231^ | 2018 | Cohort | 2015 to 2018 | Romania | IBD | ADA, IFX | HIGH RISK |
| Machado J^232^ | 2011 | Cohort | NP | Portugal | IBD | TNFi | LOW RISK |
| Mahmoud I^233^ | 2013 | Cohort | 2007 to 2012 | Tunisia | RA, SpA, Behçet, adult onset Still disease | ETN, IFX, ADA, RITUXI | LOW RISK |
| Makay B^234^ | 2019 | Cross sectional | 2012 to 2019 | Turkey | Systemic JIA, familial mediterranean fever, tumor necrosis factor receptor-associated periodic syndrome, hyper Ig D syndrome | Canakinumab | LOW RISK |
| Malaviya AN^235^ | 2010 | Cross sectional | 2009 to 2010 | India | AS, SpA, PsA, RA | IFX | LOW RISK |
| Malaviya AN^236^ | 2018 | Cohort | 2013 to 2017 | India | RA, SpA, PsA, other IMID | IFX, RITUXI, ETN, ADA, TOCI, GOLI, ABATA, SECUKI | LOW RISK |
| Malinová J^237^ | 2021 | Cohort | 2011 to 2019 | Korea | RA | bDMARD, JAKi | LOW RISK |
| Manadan AM^238^ | 2007 | Cohort | 2001 to 2008 | USA | IMID | ETN | LOW RISK |
| Mancuso JD^239^ | 2017 | Cohort | 1948 to 1998 | USA | Healthy subjects | NP | LOW RISK |
| Mankia S^240^ | 2011 | Cross sectional | 2006 to 2010 | UK | RA | IFX, ETN, ADA | VERY HIGH RISK |
| Mañosa M^241^ | 2013 | Cohort | NP | Spain | IBD | TNFi | HIGH RISK |
| Marano C^242^ | 2016 | Cross sectional | NP | Multicentric | IBD | Steroids, immunomodulators | LOW RISK |
| Mariano V^243^ | 2019 | Cross sectional | 2017 to 2018 | Brazil | IBD | AZA, TNFi | LOW RISK |
| Mariette X^244^ | 2010 | Cross sectional | NP | France | RA, SpA, IBD | Steroids, TNFi | LOW RISK |
| Mariette X^245^ | 2015 | RCT | 2007 to 2013 | France | RA | CZP | LOW RISK |
| Martin J^246^ | 2010 | Cross sectional | NP | Ireland | RA, other IMID | Steroids, MTX | LOW RISK |

| AUTHORS | YEAR OF PUBLICATION | STUDY DESIGN | TIME OF DATA COLECTION | GEOGRAPHIC LOCATION | IMIDs | DMARDs | RISK OF BIAS |
| --- | --- | --- | --- | --- | --- | --- | --- |
| Mateo L^247^ | 2009 | Cross sectional | NP | Spain | RA, AS, PsA, Spa, SLE, SAPHO, undifferentiated spondyloarthropaty | Steroids, DMARDs, TNFi | LOW RISK |
| [Matsumura R](https://pubmed.ncbi.nlm.nih.gov/?term=Matsumura+R&cauthor_id=27776599)^248^ | 2016 | Cross sectional | NP | Japan | RA | NP | LOW RISK |
| McInnes IB^249^ | 2019 | RCT | NP | Multicentric | PSO, PsA | SECUKI | LOW RISK |
| Mckay A^250^ | 1999 | Cross sectional | 1993 to 1997 | Canada | Healthy subjects | NP | LOW RISK |
| Mease PJ^251^ | 2017 | RCT | NP | Multicentric | PSO, PsA | SECUKI | LOW RISK |
| Megna M^252^ | 2021 | Cohort | 2010 to 2019 | Italy | PSO, PsA | CsA, MTX, ADA, ETN, IFX, CZP, GOLI, USTEKI, SECUKI, Ixekizumab | LOW RISK |
| Mehta B^253^ | 2015 | Cross sectional | 2007 to 2011 | USA | RA | Steroids, csDMARDs, bDMARDs | LOW RISK |
| Mehta B^254^ | 2011 | Cross sectional | NP | USA | RA | NP | LOW RISK |
| Melath S^255^ | 2014 | Cross sectional | NP | UK | IMID | immunomodulatory drugs | LOW RISK |
| Ministro P^256^ | 2011 | Cross sectional | NP | Portugal | IBD | TNFi | LOW RISK |
| Miossi R^257^ | 2013 | Cross sectional | 2004 to 2013 | Brazil | AS | TNFi | LOW RISK |
| Mittal C^258^ | 2013 | Cross sectional | 2005 to 2013 | USA | IBD | TNFi | LOW RISK |
| Mohan AK^259^ | 2004 | Cohort | 1998 to 2002 | USA | RA | ETN | HIGH RISK |
| Molnár T^260^ | 2013 | Cohort | NP | Hungary | IBD | IFX | HIGH RISK |
| Mulero P^261^ | 2012 | Cross sectional | 2009 to 2011 | Spain | Multiple Sclerosis | Natalizumab | LOW RISK |
| Muñoz L^262^ | 2015 | Cohort | 2003 to 2013 | Spain | IMID | TNFi | LOW RISK |
| Mustapha MG^263^ | 2009 | Cross sectional | NP | Nigeria | Healthy subjects | NP | LOW RISK |
| Mutlu P^264^ | 2014 | Cross sectional | 2005 to 2010 | Turkey | IMID | TNFi | LOW RISK |
| Nasr I^265^ | 2014 | Cross sectional | 2010 to 2013 | UK | IBD | Steroids, immunomodulators | LOW RISK |
| Nisar MK^266^ | 2015 | Cohort | 1998 to 2014 | UK | IMID | TNFi, CZP, RITUXI, TOCI, ABATA | LOW RISK |
| Nobre CA^267^ | 2012 | Cohort | 2005 to 2008 | Brazil | RA, AS, PsA | IFX | HIGH RISK |
| Nusslein H^268^ | 2013 | Cross sectional | 2008 to 2012 | Canada | RA | ABATA | LOW RISK |
| Ocal S^269^ | 2012 | Cross sectional | NP | Turkey | AS, RA, other IMID | TNFi | LOW RISK |
| Opris D^270^ | 2014 | Cross sectional | 2009 to 2013 | Romania | IMID | IFX, ETN, ADA, GOLI, CZP, TOCI, RITUXI | LOW RISK |
| Ortakâylâ M^271^ | 2012 | Cross sectional | NP | Turkey | RA, AS | DMARDs | LOW RISK |
| Owen L^272^ | 2016 | Cohort | 2007 to 2015 | UK | IBD | TNFi | HIGH RISK |

| AUTHORS | YEAR OF PUBLICATION | STUDY DESIGN | TIME OF DATA COLECTION | GEOGRAPHIC LOCATION | IMIDs | DMARDs | RISK OF BIAS |
| --- | --- | --- | --- | --- | --- | --- | --- |
| Özlü C^273^ | 2021 | Cohort | 2014 to 2019 | Turkey | JIA, familial mediterranean fever, hyper Ig D syndrome, cryopyrin associated periodic syndrome, uveitis, Farber disease | ADA, ETN, TOCI, canakinumab | LOW RISK |
| Paluch-Oleś J^274^ | 2013 | Cohort | NP | Poland | RA, AS | TNFi | LOW RISK |
| Papay P^275^ | 2012 | Cohort | 2006 to 2010 | Austria | IBD | TNFi | LOW RISK |
| Papay P^276^ | 2011 | Cohort | NP | Austria | IBD | Immunosuppressive medications | LOW RISK |
| Papay P^277^ | 2009 | Cohort | NP | Austria | IBD | Immunosuppressive medications | LOW RISK |
| Papp KA^278^ | 2019 | Cross sectional | NP | Canada | PsA | Risankizumab | VERY HIGH RISK |
| Park JH^279^ | 2009 | Cohort | 2007 to 2008 | Korea | RA, AS, JIA, other IMID | ETN, IFX, csDMARD, steroids | LOW RISK |
| Park JW^280^ | 2020 | Cohort | 2004 to 2017 | Korea | SLE, SS, juvenile DM, RA, MPA, EGPA, polymyositis, polyarteritis nodosa, adult-onset Still’s disease, Behçet, Sjogren, other IMID | Steroids, cyclophosphamide, MMF, CsA, MTX | LOW RISK |
| Patricia M^281^ | 2015 | Cohort | NP | Brazil | JIA | MTX, LFN, CsA, SSA, HCQ | LOW RISK |
| Pattanaik D^282^ | 2019 | Cohort | 2003 to 2014 | USA | IMID | bDMARDs | LOW RISK |
| Piñeiro R^283^ | 2012 | Cross sectional | 2003 to 2008 | Spain | Healthy subjects | NP | LOW RISK |
| Piovezani Ramos G^284^ | 2017 | Cross sectional | 1996 to 2016 | USA | IBD | IFX, ADA, vedolizumab,CZP | LOW RISK |
| Ponce de León D^285^ | 2005 | Case Control | NP | Peru | RA | cs DMARD, MTX | LOW RISK |
| Prignano F^286^ | 2014 | Cross sectional | 2008 to 2009 | Italy | PSO, PsA, hidradenitis suppurativa, Behçet, pyoderma gangrenosum | CsA, MTX, retinoids, steroids, bDMARD | LOW RISK |
| Puig L^287^ | 2020 | RCT | NP | Multicentric | PSO | Guselkumab, ADA | LOW RISK |
| Puri AS^288^ | 2017 | Cohort | 2010 to 2014 | India | IBD | IFX | HIGH RISK |
| Ramos GP^289^ | 2018 | Cohort | 2002 to 2016 | USA | IBD | IFX, ADA, vedolizumab, and CZP pegol | LOW RISK |
| Ramos L^290^ | 2014 | Cohort | 2012 to 2013 |  | IBD | IFX,ADA | HIGH RISK |
| Ramos S^291^ | 2015 | Cohort | 2008 to 2012 | Portugal | IMID | bDMARD | LOW RISK |
| Raza SH^292^ | 2016 | Cohort | 2004 to 2014 | USA | IMID | TNFi | LOW RISK |
| Ritchlin C^293^ | 2020 | RCT | NP | USA | PsA | Guselkumab | LOW RISK |
| Romiti R^294^ | 2019 | RCT | NP | Multicentric | PSO | Ixekizumab | LOW RISK |
| Rotar Z^295^ | 2020 | Cohort | 2007 to 2018 | Slovenia | RA, AS, PsA | ADA, CZP, ETN, GOLI, IFX | HIGH RISK |

| AUTHORS | YEAR OF PUBLICATION | STUDY DESIGN | TIME OF DATA COLECTION | GEOGRAPHIC LOCATION | IMIDs | DMARDs | RISK OF BIAS |
| --- | --- | --- | --- | --- | --- | --- | --- |
| Rotar Z^296^ | 2016 | Cohort | NP | Slovenia | RA | ADA, CZP, ETN, GOLI, IFX | HIGH RISK |
| Rousset S^297^ | 2020 | Cohort | 2008 to 2015 | France | Systemic vasculitis, RA, SLE, polymyalgia rheumatica, Sjogren, PsA, SpA, relapsing polychondritis, auto-inflammatory syndromes, retroperitoneal fibrosis | Steroids, immunosuppressant treatment | LOW RISK |
| Ruksenaite J^298^ | 2015 | Cross sectional | NP | UK | RA, other IMID | ADA, other TNFi | LOW RISK |
| Sahin S^299^ | 2017 | Cohort | 2003 to 2016 | Turkey | JIA, AID, uveitis, MCTD, vasculitis, IBD, idiopathic recurrent pericarditis | ETN, ADA, anakinra, IFX, RITUXI, canakinumab, TOCI, BELI | HIGH RISK |
| Sakiyama M^300^ | 2021 | Cohort | NP | Japan | Healthy subjects | NP | LOW RISK |
| Salinas MJH^301^ | 2016 | Cohort | 2015 | Argentina | RA, PsA, SLE, others | TNFi, ABATA, RITUXI, TOCI, TOFA, BELI, USTEKI, Anakinra | HIGH RISK |
| Sanchez Riera L^302^ | 2014 | Cohort | 2003 to 2011 | The United Arab Emirates | RA | bDMARDs | LOW RISK |
| Sánchez-Moya AI^303^ | 2011 | Cohort | 2004 to 2009 | Spain | PSO | TNFi | HIGH RISK |
| Saoula H^304^ | 2015 | Cross sectional | 2010 to 2014 | Algeria | IBD | TNFi | LOW RISK |
| Sargın G^305^ | 2018 | Cross sectional | NP | Turkey | RA, AS | Steroids | LOW RISK |
| Sartori NS^306^ | 2019 | Cohort | 2006 to 2016 | Brazil | RA, AS, PsA, JIA, SpA | ADA, ETN, IFX, GOLI, CZP | HIGH RISK |
| Sauzullo I^307^ | 2010 | Cohort | NP | Italy | RA, PsA, PSO, AS, IBD, Behçet | DMARDs | LOW RISK |
| Sayyahfar S^308^ | 2014 | Cross sectional | 2007 to 2008 | Iran | Healthy subjects | NP | LOW RISK |
| Schneeberger EE^309^ | 2018 | Cross sectional | NP | Colombia | RA | TOFA | LOW RISK |
| Schwartzman S^310^ | 2020 | Cross sectional | NP | USA | SpA | Ixekizumab | VERY HIGH RISK |
| Seong SS^311^ | 2007 | Cohort | 2001 to 2005 | Korea | RA | IFX, ETN | HIGH RISK |
| Shah I^312^ | 2020 | Cross sectional | NP | India | Healthy subjects | NP | LOW RISK |
| Shaikh S^313^ | 2013 | Cross sectional | NP | Canada | RA | CZP | VERY HIGH RISK |
| Shen Y^314^ | 2019 | Cohort | 2012 to 2017 | China | Behçet | IFX, ETN, ADA | HIGH RISK |
| Shimabuco AY^315^ | 2020 | Cohort | 2004 to 2018 | Brazil | AS, PsA | IFX, ADA, ETN, GOLI, CZP | HIGH RISK |
| Shin A^316^ | 2021 | Cohort | 2002 to 2016 | Korea | RA | TNFi, ABATA, TOCI | LOW RISK |
| Shobha V^317^ | 2019 | Cross sectional | NP | India | RA, SLE, AS, PsA, SpA, other | ADA, RITUXI, ETN, GOLI, IFX, TOCI, ABATA | VERY HIGH RISK |

| AUTHORS | YEAR OF PUBLICATION | STUDY DESIGN | TIME OF DATA COLECTION | GEOGRAPHIC LOCATION | IMIDs | DMARDs | RISK OF BIAS |
| --- | --- | --- | --- | --- | --- | --- | --- |
| Shobha V^318^ | 2016 | Cross sectional | NP | India | IMID | TNFi, RITUXI, TOCI, ABATA | LOW RISK |
| Shu D^319^ | 2020 | Cohort | 2019 to 2020 | China | PSO | SECUKI | LOW RISK |
| Sichletidis L^320^ | 2006 | Cohort | 2000 to 2004 | Greece | IMID | TNFi | LOW RISK |
| Siffledeen JS^321^ | 2012 | Case Control | 1963 to 2008 | NP | IBD | bDMARD | HIGH RISK |
| Simon TA^322^ | 2019 | Cohort | 2006 to 2014 | USA | RA | ABATA, Other DMARDs | HIGH RISK |
| Simon TA^323^ | 2021 | RCT | NP | Multicentric | RA | ABATA | HIGH RISK |
| Sleiman R^324^ | 2007 | Cross sectional | 2004 | Lebanon | Healthy subjects | NP | LOW RISK |
| Slogotskaya L^325^ | 2018 | Cross sectional | 2013 to 2016 | Russia | Healthy subjects | NP | LOW RISK |
| Slouma M^326^ | 2017 | Cohort | 2007 to 2014 | Tunisia | IMID | TNFi, RITUXI | LOW RISK |
| So H^327^ | 2017 | Cross sectional | 2013 to 2014 | China | AR, PsA, SpA | Steroids, MTX, SSA, LFN, HCQ, CsA | LOW RISK |
| Soare A^328^ | 2018 | Cohort | 2002 to 2014 | Romania | IMID | TNFi | LOW RISK |
| Socaci A^329^ | 2011 | Case Control | 2009 to 2010 | Romania | RA | NP | LOW RISK |
| Solovan C^330^ | 2011 | Cohort | NP | Romania | PSO | TNFi | LOW RISK |
| Son CN^331^ | 2014 | Cohort | 2008 to 2012 | Korea | AS | ETN, ADA, IFX | LOW RISK |
| Song YJ^332^ | 2021 | Cohort | 2011 to 2019 | Korea | RA | bDMARD, JAKi | LOW RISK |
| Soriano ER^333^ | 2020 | Cohort | NP | Argentina | PSO, PsA, AS | MTX, CsA, TNFi, SECUKI | LOW RISK |
| Sousa M^334^ | 2019 | Cohort | 2013 to 2017 | Portugal | IBD | TNFi | LOW RISK |
| Stevens JP | 2019 | Cross sectional | NP | USA | IBD | NP | LOW RISK |
| Stone CD^335^ | 2012 | Cohort | 2003 to 2006 | USA | IBD, RA | TNFi | HIGH RISK |
| Subramani R^336^ | 2015 | RCT | NP | India | Healthy subjects | NP | LOW RISK |
| Sundbaum JK^337^ | 2021 | Case Control | NP | Sweden | RA | DMARDs, steroids | VERY HIGH RISK |
| Sürücüoğlu S^338^ | 2020 | Case Control | NP | Turkey | PSO | csDMARDs, bDMARDs | LOW RISK |
| Sztajnbok F^339^ | 2013 | Cross sectional | NP | Brazil | JIA | MTX | LOW RISK |
| Takeda N^340^ | 2010 | Cross sectional | NP | Japan | SLE | NP | LOW RISK |
| Tam LS^341^ | 2010 | Cohort | 2004 to 2008 | China | RA | IFX, ETN, ADA | HIGH RISK |
| Tamborenea MN^342^ | 2010 | Cross sectional | NP | Argentina | RA | Steroids, MTX, LFN | LOW RISK |
| Tamura N^343^ | 2018 | Cohort | NP | Japan | RA | TOFA | HIGH RISK |
| Tan YP^344^ | 2016 | Cross sectional | 2015 | Malaysia | RA, SLE, PSO, other IMID | bDMARD | LOW RISK |

| AUTHORS | YEAR OF PUBLICATION | STUDY DESIGN | TIME OF DATA COLECTION | GEOGRAPHIC LOCATION | IMIDs | DMARDs | RISK OF BIAS |
| --- | --- | --- | --- | --- | --- | --- | --- |
| Tang I^345^ | 2020 | Cohort | 2007 to 2018 | China | RA, SpA, PsA, DM, Behçet, Still’s disease | Steroids, MTX, SSA, HCQ, LFN, AZA, CsA, IFX, GOLI, ADA, ETN, CZP, TOCI, TOFA, ABATA, RITUXI, SECUKI, USTEKI | LOW RISK |
| Taxonera C^346^ | 2018 | Cohort | NP | Multicentric | IBD | TNFi | LOW RISK |
| Taylor P^347^ | 2014 | RCT | NP | UK | RA | BARI | LOW RISK |
| Thaci D^348^ | 2014 | Cohort | 2008 to 2012 | Germany | PSO | ADA | HIGH RISK |
| Thi AA^349^ | 2018 | Cohort | 2007 to 2015 | UK | IBD | bDMARD | LOW RISK |
| Thomas K^350^ | 2020 | Cohort | NP | Greece | RA, SpA, other IMID | TNFi, non-TNFi | LOW RISK |
| Thomas K^351^ | 2017 | Cohort | NP | Greece | RA, PsA, AS, other IMID | bDMARD, csDMARDs | LOW RISK |
| Tomio J^352^ | 2017 | Cohort | 2012 | Japan | RA, IBD, PSO, other IMID | IFX, ETN, ADA, GOLI, CZP, MTX | LOW RISK |
| Tsai TF^353^ | 2012 | RCT | NP | Multicentric | PSO | USTEKI | LOW RISK |
| Tubach F^354^ | 2009 | Cohort | 2004 to 2006 | France | IMID | ETN, IFX, ADA | VERY HIGH RISK |
| Turner D^355^ | 2018 | Cohort | 2014 to 2017 | Israel | IBD | ADA | HIGH RISK |
| Ukatu N^356^ | 2019 | Cross sectional | 2010 TO 2018 | USA | PSO | SECUKI, ETN, ADA, USTEKI, ixekizumab | LOW RISK |
| Uslu S^357^ | 2019 | Cohort | 2011 to 2018 | Turkey | AS | TNFi | HIGH RISK |
| Vadan R^358^ | 2016 | Cohort | NP | Romania | IBD | TNFi | LOW RISK |
| Vaidya B^359^ | 2019 | Cross sectional | NP | Nepal | SpA | ADA | LOW RISK |
| Vajravelu RK^360^ | 2017 | Cohort | 2009 to 2014 | USA | IBD | Steroids, csDMARD, TNFi | LOW RISK |
| Vajravelu RK^361^ | 2015 | Cohort | 2009 to 2014 | USA | IBD | Steroids, other DMARDs | LOW RISK |
| Valido A^362^ | 2020 | Cross sectional | 2006 to 2019 | Portugal | IMID | bDMARD | VERY HIGH RISK |
| Van Vollenhoven R^363^ | 2020 | Cross sectional | NP | Netherlands | SLE | USTEKI | VERY HIGH RISK |
| Vassilopoulos D^364^ | 2011 | Cohort | 2088 to 2010 | Greece | RA, PsA, AS, SpA, other IMID | Steroids, DMARDs | LOW RISK |
| Vassilopoulos D^365^ | 2008 | Cross sectional | 2006 | Greece | RA, PsA, SLE, DM, AS, SS, vasculitis, polymyositis, sarcoidosis, polymyalgia rheumatica | Steroids, MTX, TNFi | LOW RISK |
| Velasco-Arnaiz E^366^ | 2018 | Cohort | 2005 to 2012 | Spain | Healthy subjects | NP | LOW RISK |
| Vélez P^367^ | 2018 | RCT | NP | Colombia | RA | TOFA | LOW RISK |
| Vencovsky J^368^ | 2010 | Cross sectional | NP | Czech Republic | RA, AS, JIA, PsA | TNFi | LOW RISK |
| Vortia E^369^ | 2018 | Cross sectional | NP | USA | IBD | IFX | LOW RISK |

| AUTHORS | YEAR OF PUBLICATION | STUDY DESIGN | TIME OF DATA COLECTION | GEOGRAPHIC LOCATION | IMIDs | DMARDs | RISK OF BIAS |
| --- | --- | --- | --- | --- | --- | --- | --- |
| Wang PH^370^ | 2020 | Cross sectional | 2013 to 2014 | Taiwan | RA, AS, PsA | MTX, LFN, SSA, HCQ, steroids, TNFi, non- TNFi | LOW RISK |
| Wang X^371^ | 2019 | Cohort | 2006 to 2015 | China | RA, AS, PsA, IBD, PSO, Felty’s syndrome, other | IFX, ADA, CZP, ETN, GOLI, ABATA, RITUXI, TOCI, TOFA, USTEKI | HIGH RISK |
| Winthrop KL^372^ | 2020 | RCT | NP | USA | RA | BARI | LOW RISK |
| Winthrop KL^373^ | 2016 | RCT | NP | Multicentric | RA | TOFA | LOW RISK |
| Winthrop KL^374^ | 2020 | RCT | NP | USA | RA | Upadacitinib | LOW RISK |
| Wolf WA^375^ | 2015 | Cohort | 2010 to 2014 | USA | IMID | Immunosuppressive medications | LOW RISK |
| Wong A^376^ | 2015 | Cohort | 2012 to 2014 | Australia | Healthy subjects | NP | LOW RISK |
| Wong SH^377^ | 2013 | Cohort | NP | Taiwan | IBD | Steroids, AZA, MTX, mercaptopurine | LOW RISK |
| Wu CY^378^ | 2019 | Cohort | 2016 to 2019 | Taiwan | PSO | SECUKI, ixekizumab | LOW RISK |
| Wu R^379^ | 2019 | Cross sectional | NP | China | IMID | Steroids, DMARDs | LOW RISK |
| Wu R^380^ | 2017 | Cross sectional | 2012 |  | SLE, vasculitis, other IMID | Steroids, DMARDs | LOW RISK |
| Xiao X^381^ | 2021 | Case Control | NP | China | SLE | Cyclophosphamide, CsA, HCQ, MMF, LFN | LOW RISK |
| Yamamoto S^382^ | 2017 | Cohort | NP | Japan | RA | TNFi | LOW RISK |
| Yamamoto S^383^ | 2011 | Cohort | NP | Japan | RA | IFX, ETN | LOW RISK |
| Yang QY^384^ | 2021 | Cohort | 2015 to 2020 | China | IBD | IFX | HIGH RISK |
| Ye L^385^ | 2017 | Cohort | 2009 to 2016 | China | IBD | IFX | LOW RISK |
| Yeo Y^386^ | 2014 | Cross sectional | 2005 to 2013 | Korea | AS, RA, IBD, other IMID | TNFi | LOW RISK |
| Yilmaz N^387^ | 2009 | Case Control | NP | Turkey | SLE | Immunosuppressive medications | LOW RISK |
| Yonekura CL^388^ | 2017 | Cohort | 2009 to 2013 | Brazil | RA | TNFi | HIGH RISK |
| Yoo HG^389^ | 2013 | Cohort | 2005 to 2011 | Korea | RA | LFN | HIGH RISK |
| Yoo IK^390^ | 2014 | Cross sectional | 2004 to 2013 | Korea | IBD, RA, AS, other | TNFi | VERY HIGH RISK |
| Yoo JW^391^ | 2014 | Cohort | 2005 to 2011 | Korea | IBD, RA, AS | IFX, ADA | HIGH RISK |
| Zanova E^392^ | 2015 | Cross sectional | 2011 to 2014 | Slovakia | RA, AS, PsA | ADA, GOLI, ETN, TOCI | LOW RISK |
| Zerôncio M^393^ | 2017 | Cross sectional | 2014 to 2016 | UK | IBD | Vedolizumab | VERY HIGH RISK |
| Zingaropoli MA^394^ | 2022 | Cohort | 2018 to 2021 | Italy | Multiple Sclerosis | DMARDs | LOW RISK |

1.	Aachari I, Tahiri L, Elolemy G, Taik FZ, Afilal S, Fellous S, et al. Active tuberculosis infection in moroccan patients with rheumatic diseases under biologic therapy: A multicenter national study. Int J Mycobacteriology [Internet]. 2022;11(2):175–82. Available from: https://www.embase.com/search/results?subaction=viewrecord&id=L638430008&from=export

2.	Abdala B, Ringer A, Ruffino J, Martinez M, Argento M, Chulibert S, et al. Skin test conversion of purified protein derivative (PPD) during treatment with anti-TNF α. Int J Rheum Dis [Internet]. 2021;24(SUPPL 2):211. Available from: https://www.embase.com/search/results?subaction=viewrecord&id=L636132121&from=export

3.	Aberra FN, Stettler N, Brensinger C, Lichtenstein GR, Lewis JD. Risk for active tuberculosis in inflammatory bowel disease patients. Clin Gastroenterol Hepatol. 2007;5(9):1070–5.

4.	Abreu C, Magro F, Santos-Antunes J, Pilão A, Rodrigues-Pinto E, Bernardes J, et al. Tuberculosis in anti-TNF-α treated patients remains a problem in countries with an intermediate incidence: Analysis of 25 patients matched with a control population. J Crohn’s Colitis [Internet]. 2013;7(10):e486–92. Available from: https://www.embase.com/search/results?subaction=viewrecord&id=L52532721&from=export

5.	Abreu C, Almeida F, Ferraz R, Dias CC, Sarmento A, Magro F. The tuberculin skin test still matters for the screening of latent Tuberculosis infections among Inflammatory Bowel Disease patients. Dig Liver Dis. 2016;48(12).

6.	Abreu C, Afonso J, Dias CC, Ruas R, Sarmento A, Magroc F. Serial tuberculosis screening in inflammatory bowel disease patients receiving anti-TNFα therapy. J Crohn’s Colitis [Internet]. 2017;11(10):1223–9. Available from: https://www.embase.com/search/results?subaction=viewrecord&id=L618693013&from=export

7.	Acar M, Sütçü M, Aktürk H, Hançerli-Torun S, Erol OB, Salman N, et al. Tuberculosis screening in pediatric patients receiving TNF-alpha inhibitor therapy. Turk J Pediatr [Internet]. 2017;59(5):503–10. Available from: https://www.embase.com/search/results?subaction=viewrecord&id=L621978000&from=export

8.	AGARWAL A, KEDIA S, JAIN S, GUPTA V, BOPANNA S, YADAV D-P, et al. High risk of tuberculosis during infliximab therapy despite tuberculosis screening in inflammatory bowel disease patients in India. Intest Res [Internet]. 2018;588–98. Available from: http://dx.doi.org/10.5217/ir.2018.00023

9.	Aggarwal R, Manadan AM, Poliyedath A, Sequeira W, Block JA. Safety of etanercept in patients at high risk for mycobacterial tuberculosis infections. J Rheumatol [Internet]. 2009;36(5):914–7. Available from: https://www.embase.com/search/results?subaction=viewrecord&id=L354862485&from=export

10.	Ahn GY, Koo BS, Son D, Kim YJ, Kang J, Lee TH, et al. SAT0575 POSITIVE QuantiFERON-TB GOLD TEST AND SEROCONVERSION OF QunatiFERON-TB GOLD TEST IS ASSOCIATED WITH INCREASED RISK OF THE DEVELOPMENT OF ACTIVE TUBERCULOSIS IN PATIENTS WITH ANKYLOSING SPONDYLITIS: RESULTS FROM A REAL-WORLD DATA OVER 20 YEARS. Ann Rheum Dis. 2020;79(Suppl 1).

11.	Akici A, Aydin V, Kadi E, Isli F, Gursoz H. Increased Risk of Tuberculosis in Patients With Rheumatologic Diseases Managed With Anti-Tnf-Α Agents: A Nationwide Retrospective Pharmacoepidemiological Cohort Study in Turkey. Clin Ther [Internet]. 2017;39(8):e57-. Available from: https://www.embase.com/search/results?subaction=viewrecord&id=L618190443&from=export

12.	Akyuz F, Cavus B, Iliaz R, Soyer OM, Ormeci A, Evirgen S, et al. Inflammatory bowel disease and mycobacteria: How much can we trust isoniazid prophylaxis during antitumor necrosis factor therapy? Eur J Gastroenterol Hepatol [Internet]. 2019;31(7):777–80. Available from: https://www.embase.com/search/results?subaction=viewrecord&id=L628078592&from=export

13.	Hajoj S Al, Varghese B, Datijan A, Shoukri M, Alzahrani A, Alkhenizan A, et al. Interferon Gamma Release Assay versus Tuberculin Skin Testing among Healthcare Workers of Highly Diverse Origin in a Moderate Tuberculosis Burden Country. PLoS One. 2016;11(5).

14.	Al-Sohaim A, Bawazir AS, Al-Turki T, Alsafi EO, Al-Roqy A, Layqah L, et al. The risk of tuberculosis infection in 410 Saudi patients receiving adalimumab therapy. Ann Saudi Med [Internet]. 2021;41(5):285–92. Available from: https://www.embase.com/search/results?subaction=viewrecord&id=L2015096423&from=export

15.	Al-Taweel T, Pai M, Strohl M, Bessissow T, Bitton A, Seidman EG, et al. A pilot study of optimal screening for latent tuberculosis in patients with inflammatory. Gastroenterology [Internet]. 2014;146(5):S-582. Available from: https://www.embase.com/search/results?subaction=viewrecord&id=L71457786&from=export

16.	Alasan F, Balbay EG, Cangur S, Balbay O, Aydin LY, Annakkaya AN. Should isoniazid prophylaxis be prescribed to the patients under tumor necrosis factor-alpha antagonists independent of tuberculin skin test? Eur Respir J [Internet]. 2015;46((Alasan F.; Balbay E.G.; Cangur S.; Balbay O.; Aydin L.Y.; Annakkaya A.N.)). Available from: https://www.embase.com/search/results?subaction=viewrecord&id=L72108621&from=export

17.	Algaba A, Guerra I, Guardiola A, Bonillo D, Granja A, Bernal P, et al. Serious pulmonary infections in patients with inflammatory bowel disease on treatment with anti-TNF. J Crohn’s Colitis [Internet]. 2018;12((Algaba A.; Guerra I.; Guardiola A.; Bonillo D.; Granja A.; Bernal P.; Aller M.D.M.; Bermejo F.) Hospital Universitario de Fuenlabrada, Gastroenterology, Fuenlabrada, Spain(Hernández B.) Hospital Universitario de Fuenlabrada, Pharmacy, Fuenlabrada, Spain():S521–2. Available from: https://www.embase.com/search/results?subaction=viewrecord&id=L621354358&from=export

18.	Alkadi A, Alduaiji N, Alrehaily A. Risk of tuberculosis reactivation with rituximab therapy. Int J Heal Sci. 2017;11(2):41–4.

19.	Amorim RF, Esberard BC, Chinem ES, Correa RS, Rodrigues L, Ribeiro-Alves M, et al. Superiority of Interferon-Gamma Release Assay over Tuberculin Skin Test for Screening Latent Tuberculosis Prior to and During Biological Therapy in Inflammatory Bowel Disease in Rio De Janeiro, Brazil. Gastroenterology [Internet]. 2018;154(6):S-359. Available from: https://www.embase.com/search/results?subaction=viewrecord&id=L2002432602&from=export

20.	Andrisani G, Papa A, Armuzzi A, Ardito F, Marzo M, Felice C, et al. Tubercolosis screening in Italian patients affected by inflammatory bowel disease: Comparison of quantiferon-tb gold versus tuberculin skin test. Dig Liver Dis [Internet]. 2010;42:S181–2. Available from: https://www.embase.com/search/results?subaction=viewrecord&id=L70146187&from=export

21.	Anzueto E, Rivera SM, Asnal C, Cruz LJ, Caicedo T, Felipe O, et al. Tofacitinib in rheumatoid arthritis: An updated analysis of real-world data in latin america. J Clin Rheumatol [Internet]. 2019;25(3):S50-. Available from: https://www.embase.com/search/results?subaction=viewrecord&id=L627248055&from=export

22.	Archimandriti D, Iliou C, Tzallas A, Pelechas E, Drosos A, Voulgari P. AB0411 Comparison of tuberculin skin test and interferon-gamma release assay screening in patients with rheumatoid arthritis starting anti-tumor necrosis factor therapy. In 2017.

23.	Argüder E, Yanık Üstüner G, Ekici R, Kılıç H, Erten Ş, Karalezli A. Tuberculosis risk in patients with rheumatologic disease treated with biologic drugs. Tuberk Toraks. 2020;68(3).

24.	Arias M, de Francisco R, Riestra S, Pando A, Palacios JJ, Escalante P, et al. Performance of Two Interferon-Gamma Release Assays (T-SPOT.TB and QuantiFERON-TB Gold in Tube) Increase Diagnostic Yield of Tuberculin Skin Testing for Detection of Latent Tuberculosis in Patients With Inflammatory Bowel Disease. Gastroenterology. 2011;140(5).

25.	Askling J, Fored CM, Brandt L, Baecklund E, Bertilsson L, Cöster L, et al. Risk and case characteristics of tuberculosis in rheumatoid arthritis associated with tumor necrosis factor antagonists in Sweden. Arthritis Rheum [Internet]. 2005;52(7):1986–92. Available from: https://www.embase.com/search/results?subaction=viewrecord&id=L40994319&from=export

26.	Atteno M, Costa L, Matarese A, Caso F, Del Puente A, Cantarini L, et al. The use of TNF-α blockers in psoriatic arthritis patients with latent tuberculosis infection. Clin Rheumatol [Internet]. 2014;33(4):543–7. Available from: https://www.embase.com/search/results?subaction=viewrecord&id=L53015599&from=export

27.	Aydin V, Akici A, Isli F, Aksoy M, Aydin M, Gursoz H. Relative risk of tuberculosis in patients with rheumatic diseases managed with anti-tumour necrosis factor-alpha therapy: A nationwide cohort study. J Clin Pharm Ther. 2019;44(4).

28.	Aydin V, Akici A, Isli F, Aksoy M, Gursoz H. Ongoing elevated risk of developing tuberculosis in rheumatological conditions where Anti-TNF-alpha agents were used: Results of 2-year retrospective cohort study. Drug Saf [Internet]. 2017;40(10):1028. Available from: https://www.embase.com/search/results?subaction=viewrecord&id=L618778603&from=export

29.	Bachelez H, Gordon K, Blauvelt A, Wu J, Sinvhal R, Photowala H, et al. Short-and long-term safety of risankizumab in patients with moderate-to-severe psoriasis: An integrated analysis. J Eur Acad Dermatology Venereol [Internet]. 2019;33((Bachelez H.) Saint-Louis Hospital, Sorbonne Paris Cite University Paris Diderot, Paris, France(Gordon K.) Medical College of Wisconsin, Milwaukee, United States(Blauvelt A.) Oregon Medical Research Center, Portland, United States(Wu J.) Dermatology Resea):37–8. Available from: https://www.embase.com/search/results?subaction=viewrecord&id=L627804890&from=export

30.	Baddley JW, Winthrop KL, Chen L, Liu L, Grijalva CG, Delzell E, et al. Non-viral opportunistic infections in new users of tumour necrosis factor inhibitor therapy: results of the SAfety Assessment of Biologic ThERapy (SABER) study. Ann Rheum Dis. 2014;73(11):1942–8.

31.	Baldassano R, Ruemmele F, Rosh J, Faubion W, Kierkus J, Hyams J, et al. Long-term safety of adalimumab in pediatric patients with crohn’s disease. Inflamm Bowel Dis [Internet]. 2013;19:S97. Available from: https://www.embase.com/search/results?subaction=viewrecord&id=L71355983&from=export

32.	Barosa R, Patita M, Roque Ramos L, Nunes G, Figueiredo P, Carmo Vieira A, et al. Tuberculosis infection in an inflammatory bowel disease cohort of patients receiving anti-TNF therapy in a moderate to high tuberculosis incidence area in Europe. United Eur Gastroenterol J [Internet]. 2016;4(5):A644-. Available from: https://www.embase.com/search/results?subaction=viewrecord&id=L619936141&from=export

33.	Bartalesi F, Vicidomini S, Goletti D, Fiorelli C, Fiori G, Melchiorre D, et al. QuantiFERON-TB Gold and the TST are both useful for latent tuberculosis infection screening in autoimmune diseases. Eur Respir J. 2009;33(3).

34.	Barut K, Şahin S, Adrovic A, Köşker M, Kiliç Ö, Camcioğlu Y, et al. Tuberculin skin test response in patients with juvenile idiopathic arthritis on anti-tnf therapy. Turkish J Med Sci. 2018;48(6).

35.	Basyal RK, Jagdish RK, Alawa HL, Kanjilal M, Kumar U. Ten years’ experience with infliximab at a tertiary care center in north India. Indian J Rheumatol [Internet]. 2014;9(5):S2-. Available from: https://www.embase.com/search/results?subaction=viewrecord&id=L619194968&from=export

36.	Bautista-Molano W, González L, Fernández-Ávila D, Cardozo R, Ruiz Ó. Frecuencia de positividad de la prueba de intradermorreacción a tuberculina en una cohorte de pacientes con artritis reumatoide. Biomédica. 2021;41(3).

37.	Benhadou F, Dirix V, Domont F, Willaert F, Van Praet A, Locht C, et al. Tuberculosis Risk Stratification of Psoriatic Patients Before Anti-TNF-α Treatment. Front Immunol. 2021;12.

38.	Bensen W, Nüßlein H, Alten R, Galeazzi M, Lorenz H-M, Boumpas D, et al. Real-world efficacy and safety of abatacept treatment for rheumatoid arthritis: 12-Month interim analysis of the action study. J Rheumatol [Internet]. 2013;40(6):985. Available from: https://www.embase.com/search/results?subaction=viewrecord&id=L71621609&from=export

39.	Bensen W, Kelsall J, Choquette D, Avina-Zubieta A, Chow A, Sholter D, et al. What is the real-world effectiveness and safety of golimumab in the treatment of rheumatoid arthritis over a 12 month period? J Rheumatol [Internet]. 2015;42(7):1297. Available from: https://www.embase.com/search/results?subaction=viewrecord&id=L72204972&from=export

40.	Benzaquén N, Haye Salinas M, Pirola J, Retamozo S, Caeiro F, Alvarellos A, et al. Tuberculosis in a registry of rheumatic patients treated with biological drugs. Ann Rheum Dis [Internet]. 2016;75((Benzaquén N.; Haye Salinas M.; Pirola J.; Retamozo S.; Caeiro F.; Alvarellos A.; De La Vega M.; Casado G.; Gomez G.; Citera G.; Gallardo M.; Quinteros A.; Exeni I.; Medina M.; Astesana P.; Sanchez Andia C.; Sarano J.; Granel A.; Peluzzon A.; Cappucciona):847. Available from: https://www.embase.com/search/results?subaction=viewrecord&id=L612778851&from=export

41.	Bermejo F, Algaba A, Chaparro M, Taxonera C, Garrido E, García-Arata I, et al. How frequently do tuberculosis screening tests convert in inflammatory bowel disease patients on anti-tumour necrosis factor-alpha? A pilot study. Dig Liver Dis [Internet]. 2013;45(9):733–7. Available from: https://www.embase.com/search/results?subaction=viewrecord&id=L52535954&from=export

42.	Beshir MR, Zidan AE, El-Saadny HF, Ramadan RA, Karam NA, Amin EK, et al. Evaluation of the Immune Response to Interferon Gamma Release Assay and Tuberculin Skin Test among BCG Vaccinated Children in East of Egypt. Med (United States). 2016;95(17).

43.	Bierrenbach AL, Cunha SS, Barreto ML, Pereira SM, Dourado I, Ichihara MY, et al. Tuberculin reactivity in a population of schoolchildren with high BCG vaccination coverage. Rev Panam Salud Publica/Pan Am J Public Heal. 2003;13(5).

44.	Blauvelt A, Strober B, Langley R, Kavanagh S, Arendt C, Boehnlein M, et al. Safety of certolizumab pegol in plaque psoriasis: Pooled 96-week data from three phase 3, multicenter, randomized, placebo-controlled studies (CIMPASI-1, CIMPASI-2 and CIMPACT). J Eur Acad Dermatology Venereol [Internet]. 2019;33((Blauvelt A.) Oregon Medical Research Center, Portland, United States(Strober B.) University of Connecticut Health Center, Farmington, United States(Strober B.) Probity Medical Research, Waterloo, United States(Langley R.) Dalhousie University, NS, Canada):21–2. Available from: https://www.embase.com/search/results?subaction=viewrecord&id=L627804969&from=export

45.	Bonfiglioli KR, Ribeiro ACM, Moraes JCB, Saad CGS, Souza FHC, Calich AL, et al. LTBI screening in rheumatoid arthritis patients prior to anti-TNF treatment in an endemic area (International Journal of Tuberculosis and Lung Disease (2014) 18, 8 (905-911)). Vol. 18, International Journal of Tuberculosis and Lung Disease. 2014.

46.	Borekci S, Atahan E, Demir Yilmaz D, Mazlcan N, Duman B, Ozguler Y, et al. Factors affecting the tuberculosis risk in patients receiving anti-tumor necrosis factor-α treatment. Respiration [Internet]. 2015;90(3):191–8. Available from: https://www.embase.com/search/results?subaction=viewrecord&id=L605175499&from=export

47.	Bowerman RJ. Tuberculin skin testing in BCG-vaccinated populations of adults and children at high risk for tuberculosis in Taiwan. Int J Tuberc Lung Dis. 2004;8(10).

48.	Bozaykut A, Ozahi Ipek I, Ozkars MY, Pulat Seren L, Atay E, Atay Z. Effect of BCG vaccine on tuberculin skin tests in 1-6-year-old children. Acta Paediatr Int J Paediatr. 2002;91(2).

49.	Brassard P, Lowe AM, Bernatsky S, Kezouh A, Suissa S. Rheumatoid arthritis, its treatments, and the risk of tuberculosis in Quebec, Canada. Arthritis Rheum. 2009;61(3):300–4.

50.	Brunelli JB, Bonfiglioli KR, Silva CA, Kozu KT, Goldenstein‐Schainberg C, Bonfa E, et al. Latent tuberculosis infection screening in juvenile idiopathic arthritis patients preceding anti‐TNF therapy in a tuberculosis high‐risk country. Rev Bras Reumatol [Internet]. 2017;57(5):392–6. Available from: https://www.embase.com/search/results?subaction=viewrecord&id=L618953002&from=export

51.	Bruzzese V, Lorenzetti R, Zullo A, Hassan C, Campo SM. Anti-TNF therapy and tuberculosis risk in rheumatic diseases, psoriasis, and IBD: A pooled-data analysis of randomized controlled trials. Ann Rheum Dis [Internet]. 2013;72((Bruzzese V.; Lorenzetti R.; Zullo A.; Hassan C.; Campo S.M.) Internal Medicine, Rheumatology and Gastroenterology, Nuovo Regina Margherita Hospital, Rome, Italy). Available from: https://www.embase.com/search/results?subaction=viewrecord&id=L71332377&from=export

52.	Bugiani M, Borraccino A, Migliore E, Carosso A, Piccioni P, Cavallero M, et al. Tuberculin reactivity in adult BCG-vaccinated subjects: A cross-sectional study. Int J Tuberc Lung Dis. 2003;7(4).

53.	Burmester GR, Landewé R, Genovese MC, Friedman AW, Pfeifer ND, Varothai NA, et al. Adalimumab long-term safety: Infections, vaccination response and pregnancy outcomes in patients with rheumatoid arthritis. Ann Rheum Dis [Internet]. 2017;76(2):414–7. Available from: https://www.embase.com/search/results?subaction=viewrecord&id=L611182284&from=export

54.	Byun JM, Lee CK, Rhee SY, Kim H-J, Im JP, Park DI, et al. Risks for opportunistic tuberculosis infection in a cohort of 873 patients with inflammatory bowel disease receiving a tumor necrosis factor-α inhibitor. Scand J Gastroenterol [Internet]. 2014;50(3):312–20. Available from: https://www.embase.com/search/results?subaction=viewrecord&id=L602249974&from=export

55.	Cabriada JL, Ruiz-Zorrilla R, Barrio J, Atienza R, Huerta A, Rodriguez-Lago I, et al. Screening for latent tuberculosis infection in patients with inflammatory bowel disease: Can interferon-gamma release assays replace the tuberculin skin test? Turkish J Gastroenterol. 2019 Aug 7;29(3):292–8.

56.	Cadavid M, Acosta LT, Jiménez SB, Londoño AM. Agreement between QuantiFERON-TB Gold test and tuberculin skin test results for the diagnosis of latent tuberculosis infection in patients eligible for biologic therapy management. Dermatologia Rev Mex [Internet]. 2016;60(4):283–94. Available from: https://www.embase.com/search/results?subaction=viewrecord&id=L611986613&from=export

57.	Cagatay T, Bingol Z, Kıyan E, Yegin Z, Okumus G, Arseven O, et al. Follow-up of 1887 patients receiving tumor necrosis-alpha antagonists: Tuberculin skin test conversion and tuberculosis risk. Clin Respir J [Internet]. 2018;12(4):1668–75. Available from: https://www.embase.com/search/results?subaction=viewrecord&id=L619206592&from=export

58.	Cagatay T, Kilicaslan Z, Cagatay P, Mertsoylu M, Gulbaran Z, Yildiz R, et al. TNF-alpha antagonist therapy modify the tuberculin skin test response. Rheumatol Int [Internet]. 2011;31(9):1147–51. Available from: https://www.embase.com/search/results?subaction=viewrecord&id=L50852165&from=export

59.	Callado MRM, Lima JRC, Nobre CA, Vieira WP. Low prevalence of reactive PPD prior to infliximab use: Comparative study on a population sample of Hospital Geral de Fortaleza. Rev Bras Reumatol [Internet]. 2011;51(1):40–52. Available from: https://www.embase.com/search/results?subaction=viewrecord&id=L361489498&from=export

60.	Calzada-Hernández J, Anton J, Núñez E, Mellado MJ, Martín FJ, Fernández L, et al. Diagnosis of Tuberculosis Infection in Pediatric Patients Treated with Tumor Necrosis Factor Alpha Inhibitors; A Multicenter Spanish Study Comparing IGRA and Tuberculin Skin Tests. Ann Rheum Dis. 2015 Jun 9;74(Suppl 2):386.1-386.

61.	Calzada-Hernández J, Anton-López J, Bou-Torrent R, Iglesias-Jiménez E, Ricart-Campos S, Martín de Carpi J, et al. Tuberculosis in pediatric patients treated with anti-TNFα drugs: a cohort study. Pediatr Rheumatol. 2015 Dec 3;13(1):54.

62.	Çamlar SA, Makay B, Appak O, Çaʇan Y, Esen N, Günay T, et al. Performance of tuberculin skin test and interferon gamma assay for the diagnosis of latent tuberculosis infection in juvenile idiopathic arthritis. Clin Exp Rheumatol [Internet]. 2011;29(2):386. Available from: https://www.embase.com/search/results?subaction=viewrecord&id=L70503760&from=export

63.	Campanilho-Marques R, Deakin C, Simou S, Wedderburn LR, Pilkington C. Efficacy and safety of tumour necrosis factor antagonists in a large cohort of juvenile dermatomyositis patients. Arthritis Rheumatol [Internet]. 2016;68((Campanilho-Marques R.) Infection, Inflammation and Rheumatology Section, UCL, Institute of Child Health, London, Portugal(Campanilho-Marques R.; Wedderburn L.R.) Rheumatology, Great Ormond Street Hospital for Children NHS Trust, London, United Kingdom(Ca):1709. Available from: https://www.embase.com/search/results?subaction=viewrecord&id=L613889542&from=export

64.	Carvalho L, Túlio MA, Carmo J, Rodrigues J, Bana T, Costa E, et al. Tuberculosis in inflammatory bowel disease under tumour necrosis factor alpha antagonist-the risk beyond screening. United Eur Gastroenterol J [Internet]. 2017;5(5):A726–7. Available from: https://www.embase.com/search/results?subaction=viewrecord&id=L619891037&from=export

65.	Castellani C, Molteni E, Altobelli A, Garufi C, Mancuso S, Spinelli FR, et al. Are interferon-gamma release assays reliable to detect tuberculosis infection in patients with rheumatoid arthritis treated with janus kinase inhibitors. Ann Rheum Dis [Internet]. 2021;80(SUPPL 1):1160–1. Available from: https://www.embase.com/search/results?subaction=viewrecord&id=L635709994&from=export

66.	Çekiç C, Aslan F, Vatansever S, Topal F, Yüksel ES, Alper E, et al. Latent tuberculosis screening tests and active tuberculosis infection rates in Turkish inflammatory bowel disease patients under anti-tumor necrosis factor therapy. Ann Gastroenterol [Internet]. 2015;28(2):241–6. Available from: https://www.embase.com/search/results?subaction=viewrecord&id=L603565014&from=export

67.	Cerda OL, De Los Angeles Correa M, Granel A, Marcos AI, Giraldo CL, Rillo OL, et al. Tuberculin conversion in patients with autoimmune arthropathies receiving biologic therapy. Arthritis Rheumatol [Internet]. 2014;66((Cerda O.L.; Citera G.) Instituto de Rehabilitación Psicofísica, Buenos Aires, Argentina(De Los Angeles Correa M.) Instituto de Rehabilitación Psicofísica de Buenos Aires, Buenos Aires, Argentina(Granel A.; Marcos A.I.) Hospital San Roque de Gonnet, La Pl):S897–8. Available from: https://www.embase.com/search/results?subaction=viewrecord&id=L71738033&from=export

68.	Cerda OL, M de LAC, Granel A, Marcos AI, Giraldo C, Rillo O, et al. Tuberculin test conversion in patients with chronic inflammatory arthritis receiving biological therapy. Eur J Rheumatol. 2019;6(1):19–22.

69.	Chaer FGG, Valim JM de L, Reis RC, Klautau GB, Souza BDB de. Use of biologic agents and risk of tuberculosis in Brazil, a tuberculosis high-burden country. Drugs Context. 2020 Jul 28;9:1–7.

70.	Chan M-J, Wen Y-H, Huang Y-B, Chuang H-Y, Tain Y-L, Lily Wang Y-C, et al. Risk of tuberculosis comparison in new users of antitumour necrosis factor-α and with existing disease-modifying antirheumatic drug therapy. J Clin Pharm Ther [Internet]. 2018;43(2):256–64. Available from: https://www.embase.com/search/results?subaction=viewrecord&id=L619216355&from=export

71.	Chandra A, Kanth R, Thareja S. Efficacy and safety of adalimumab Biosimilar (Exemptia) in moderate-to-severe steroid- refractory ulcerative colitis patients: Real-life outcomes in resource-constrained setting at 24-weeks follow-up. Biol Targets Ther [Internet]. 2019;13:191–200. Available from: https://www.embase.com/search/results?subaction=viewrecord&id=L2003257423&from=export

72.	Chang B, Park H, Jeon K, Ahn J, Cha H, Koh E, et al. Interferon-gamma assay in the diagnosis of latent tuberculosis infection in arthritis patients treated with tumor necrosis factor antagonists in Korea. Am J Respir Crit Care Med [Internet]. 2010;181(1). Available from: https://www.embase.com/search/results?subaction=viewrecord&id=L70842222&from=export

73.	Chang H-C, Chen L-C, Wu J-S, Tseng H-L. The utilization of biological agents in rheumatoid arthritis patients in Southern Taiwan. Pharmacoepidemiol Drug Saf [Internet]. 2010;19((Chang H.-C.; Chen L.-C.) Graduate Institute of Clinical Pharmacy, Kaohsiung Medical University, Kaohsiung, Taiwan(Chen L.-C.) Division for Social Research in Medicines and Health, School of Pharmacy, University of Nottingham, Nottingham, United Kingdom(W):S314–5. Available from: https://www.embase.com/search/results?subaction=viewrecord&id=L70450285&from=export

74.	Cheah C, Abdul Rahim R, Chembalingam CG, Ramanujam S, Mohd Noor N, D’Souza BA, et al. Mantoux test conversion rate amongst patients with rheumatic diseases receiving biologic-DMARDs: Single center experience. Int J Rheum Dis [Internet]. 2015;18:76. Available from: https://www.embase.com/search/results?subaction=viewrecord&id=L72004832&from=export

75.	Chen D-Y, Li J-P, Chen Y-M, Liao T-L, Chen H-H, Hsieh C-W, et al. Elevated Neopterin Levels Are Associated with Increased Tuberculosis Risk in Rheumatoid Arthritis Patients with QuantiFERON Conversion during Biologic Therapy. PLoS One. 2016 Nov 18;11(11):e0166301.

76.	Chen H-H, Chen Y-M, Lai K-L, Lin C-H, Chen D-Y, Lim C-H, et al. One-year tuberculosis risk in rheumatoid arthritis patients initiating tumor necrosis factor-α inhibitor from 2003 to 2011 in Taiwan: A nationwide population-based cohort study. Ann Rheum Dis [Internet]. 2016;75((Chen H.-H.; Chen Y.-M.; Lin C.-H.) Department of Medical Research, Taichung, Taiwan(Lai K.-L.) Division of Allergy, Immunology and Rheumatology, Taichung, Taiwan(Chen D.-Y.) Department of Medical Education, Taichung Veterans General Hospital, Taichung, T):649. Available from: https://www.embase.com/search/results?subaction=viewrecord&id=L612777706&from=export

77.	Chen Y-J, Wu C-Y, Shen J-L, Chen T-T, Chang Y-T. Association between traditional systemic antipsoriatic drugs and tuberculosis risk in patients with psoriasis with or without psoriatic arthritis: Results of a nationwide cohort study from Taiwan. J Am Acad Dermatol. 2013 Jul;69(1):25–33.

78.	Chiacchio T, Petruccioli E, Vanini V, Cuzzi G, Massafra U, Baldi G, et al. Characterization of QuantiFERON-TB-Plus results in latent tuberculosis infected patients with or without immune-mediated inflammatory diseases. J Infect [Internet]. 2019;79(1):15–23. Available from: https://www.embase.com/search/results?subaction=viewrecord&id=L2001850076&from=export

79.	Chiu H-Y, Hsueh P-R, Tsai T-F. Clinical experience of QuantiFERON®-TB Gold testing in patients with psoriasis treated with tumour necrosis factor blockers in Taiwan. Br J Dermatol [Internet]. 2011;164(3):553–9. Available from: https://www.embase.com/search/results?subaction=viewrecord&id=L361401016&from=export

80.	Chiu YM, Lang HC, Lin HY, Yang MT, Fang CH, Yang YW, et al. Risk of tuberculosis, serious infection and lymphoma with disease-modifying biologic drugs in rheumatoid arthritis patients in Taiwan. Int J Rheum Dis. 2014;17 Suppl 3:9–19.

81.	Cho H, Kim YW, Suh C-H, Jung J-Y, Um Y-J, Jung J-H, et al. Concordance between the tuberculin skin test and interferon gamma release assay (IGRA) for diagnosing latent tuberculosis infection in patients with systemic lupus erythematosus and patient characteristics associated with an indeterminate IGRA. Lupus. 2016 Oct 11;25(12):1341–8.

82.	Cho SI, Kang S, Kim YE, Lee JY, Jo SJ. Ustekinumab does not increase tuberculosis risk: Results from a national database in South Korea. J Am Acad Dermatol [Internet]. 2020;82(5):1243–5. Available from: https://www.embase.com/search/results?subaction=viewrecord&id=L2005079027&from=export

83.	Chung TT, Ko HJ, Lau CS, Chung HY. A retrospective study on the risk of tuberculosis in patients with rheumatoid arthritis. Rheumatol Int [Internet]. 2020;40(6):983–90. Available from: https://www.embase.com/search/results?subaction=viewrecord&id=L2004752066&from=export

84.	Ciang NC-O, Chan SCW, Lau CS, Chiu ETF, Chung HY. Risk of tuberculosis in patients with spondyloarthritis: data from a centralized electronic database in Hong Kong. BMC Musculoskelet Disord. 2020 Dec 10;21(1):832.

85.	Cohen S, Tanaka Y, Mariette X, Curtis JR, Lee EB, Nash P, et al. Long-term safety of tofacitinib up to 9.5 years: A comprehensive integrated analysis of the ra clinical development program. Arthritis Rheumatol [Internet]. 2018;70((Cohen S.) Metroplex Clinical Research Center, University of Texas, Southwestern Medical Center, Dallas, TX, United States(Tanaka Y.) University of Occupational and Environmental Health, Kitakyushu, Japan(Mariette X.) Paris-Sud University, Le Kremlin Bicê):1076–7. Available from: https://www.embase.com/search/results?subaction=viewrecord&id=L626434975&from=export

86.	Conti A, Piaserico S, Gisondi P, Odorici G, Galdo G, Lasagni C, et al. Management of long-term therapy with biological drugs in psoriatic patients with latent tuberculosis infection in real life setting. Dermatol Ther [Internet]. 2017;30(5). Available from: https://www.embase.com/search/results?subaction=viewrecord&id=L616477900&from=export

87.	Coskunol I, Baysak A, Dalli A, Uluorman F, Can G. Anti-TNF-alpha therapy in patients with latent tuberculosis incidence. Eur Respir J [Internet]. 2015;46((Coskunol I.; Baysak A.; Dalli A.; Uluorman F.; Can G.)). Available from: https://www.embase.com/search/results?subaction=viewrecord&id=L72108619&from=export

88.	Costantino F, M de CB, Rat AC, Loeuille D, Dintinger H, Béné MC, et al. Screening for latent tuberculosis infection in patients with chronic inflammatory arthritis: discrepancies between tuberculin skin test and interferon-γ release assay results. J Rheumatol. 2013;40(12):1986–93.

89.	Costantino F, Faure G, De Carvalho M, Ra A, Dintinger H, Loeuille D, et al. High Level of Disease Activity in Chronic Inflammatory Rheumatisms Increases the Rate of Indeterminate Interferon-Gamma-Release Assay Results for Latent Tuberculosis Infection Detection. Arthritis Rheum. 2010;62(supp 10):768.

90.	Cuomo G, D’Abrsca V, Iacono D, Pantano I. The conversion rate of tuberculosis screening tests during biological therapies in patients with rheumatoid arthritis. Clin Rheumatol [Internet]. 2017;36(2):457–61. Available from: https://www.embase.com/search/results?subaction=viewrecord&id=L613201839&from=export

91.	Dashdamirova S, Hatemi AI, Erzin YZ, Tuncer M, Celik AF. TUberculosis infection in inflammatory bowel disease patients who received biologic agents: A single center experience of 20 years. United Eur Gastroenterol J [Internet]. 2019;7(8):629–30. Available from: https://www.embase.com/search/results?subaction=viewrecord&id=L632138113&from=export

92.	Davì S, Verazza S, Consolaro A, Insalaco A, Gerloni V, Cimaz R, et al. Evaluation of the disease course of italian children with juvenile idiopathic arthritis treated with etanercept: Preliminary results in 1019 patients. Ann Rheum Dis [Internet]. 2015;74((Davì S.; Verazza S.; Consolaro A.; Alpigiani M.G.; Martini A.; Ravelli A.) Istituto Giannina Gaslini, Genova, Italy(Insalaco A.) Ospedale Bambin Gesù, Roma, Italy(Gerloni V.) Istituto Gaetano Pini, Milano, Italy(Cimaz R.) Az.Ospedaliera-Universitaria Mey):1239–40. Available from: https://www.embase.com/search/results?subaction=viewrecord&id=L72154428&from=export

93.	De Castro ML, Hernández V, Pineda JR, Pereira S, Martínez-Cadilla J, Sanromán L, et al. Tuberculosis infection in inflammatory bowel disease patients after anti-TNF therapy in a high tuberculosis prevalence rate area. J Crohn’s Colitis [Internet]. 2014;8:S191–2. Available from: https://www.embase.com/search/results?subaction=viewrecord&id=L71388969&from=export

94.	De Francisco R, Arias M, Pando A, Palacios J, Escalante P, Perez-Martinez I, et al. Interferon-gamma release assays (T-SPOT.TB and QuantiFERON-TB GOLD in tube) versus tuberculin skin testing for detection of latent tuberculosis in patients with inflammatory bowel disease. J Crohn’s Colitis [Internet]. 2011;5(1):S52–3. Available from: https://www.embase.com/search/results?subaction=viewrecord&id=L70476847&from=export

95.	De Francisco R, Arias-Guillén M, Castaño-García A, Pérez-Martínez I, Palacios JJ, Rolle-Sóñora V, et al. Risk of tuberculosis in patients with inflammatory bowel disease receiving biologics using two interferon-γ release assays as monitoring. J Crohn’s Colitis [Internet]. 2019;13((De Francisco R.; Castaño-García A.; Pérez-Martínez I.; Martínez-González S.; Jiménez-Beltrán V.; Rodríguez-Ferreiro N.; Flórez-Díez P.; Suárez A.; Riestra S.) Hospital Universitario Central De Asturias, Gastroenterology, Oviedo, Spain(De Francisco R.; Ro):S480–1. Available from: https://www.embase.com/search/results?subaction=viewrecord&id=L628298313&from=export

96.	De Leon DP. Comparison of IGRAs with TST for the detection of LTBI in RA patients in a TB endemic population. Int J Tuberc Lung Dis [Internet]. 2010;14(6 SUPPL. 1):S40–1. Available from: https://www.embase.com/search/results?subaction=viewrecord&id=L360026350&from=export

97.	de Medeiros VLS, Santos FCF, Montenegro LML, Silva M da C, Souza VMO de, Lima Neto RG de, et al. TST conversions and systemic interferon-gamma increase after methotrexate introduction in psoriasis patients. PLoS One. 2020 Dec 3;15(12):e0242098.

98.	Del Tedesco E, Oussalah A, Laharie D, Biroulet LP, Phelip JM, Roblin X. Interferon gamma release assay (Igra) and/or tuberculin skin test (Tst) in inflammatory bowel disease population: Discordance and performance. Best strategy for detecting tuberculosis. Gastroenterology [Internet]. 2010;138(5):S672–3. Available from: https://www.embase.com/search/results?subaction=viewrecord&id=L70397965&from=export

99.	Dixon WG, Hyrich KL, Watson KD, Lunt M, Galloway J, Ustianowski A, et al. Drug-specific risk of tuberculosis in patients with rheumatoid arthritis treated with anti-TNF therapy: Results from the British Society for Rheumatology Biologics Register (BSRBR). Ann Rheum Dis [Internet]. 2010;69(3):522–8. Available from: https://www.embase.com/search/results?subaction=viewrecord&id=L358461415&from=export

100.	Dogan C, Kiral N, Cömert S, Fidan A, Salepci B, Caglayan B. The incidence of tuberculosis in patients taking anti TNF alpha therapy. Eur Respir J [Internet]. 2011;38. Available from: https://www.embase.com/search/results?subaction=viewrecord&id=L72117157&from=export

101.	Eddarami J, Ichchou L. Quantiferon TB gold test in diagnosis of latent tuberculosis infection among a Moroccan population with spondyloarthritis. Ann Rheum Dis [Internet]. 2018;77((Eddarami J.; Ichchou L.) Department of Rheumatology, Mohamed VI University Hospital, Mohammed I University, Oujda, Morocco):1563. Available from: https://www.embase.com/search/results?subaction=viewrecord&id=L623993657&from=export

102.	Edet A, Turner S. Factors associated with positive Mantoux results in NHS healthcare workers. Occup Med (Chic Ill). 2018 May 23;68(4):286–9.

103.	Ehrenstein B, Nessyt F, Hartung W, Meyringer R, Fleck M. Utility of an interferon-gamma-release assay to detect latent tuberculosis in patients evaluated for immunosuppressive therapy with biologic agentsa retrospective analysis of routine clinical practice in a low-prevalence population. Arthritis Rheum. 2009;60(supp 10):980.

104.	Elbek O, Uyar M, AydIn N, Börekçi S, Bayram N, Bayram H, et al. Increased risk of tuberculosis in patients treated with antitumor necrosis factor alpha. Clin Rheumatol [Internet]. 2009;28(4):421–6. Available from: https://www.embase.com/search/results?subaction=viewrecord&id=L50353525&from=export

105.	Escalante P, Kooda KJ, Khan R, Aye SS, Christianakis S, Arkfeld DG, et al. Diagnosis of Latent Tuberculosis Infection with T-SPOT®.TB in a Predominantly Immigrant Population with Rheumatologic Disorders. Lung. 2015 Feb 16;193(1):3–11.

106.	Fajardo Hermosillo LD. Factors associated with active and latent tuberculosis, tuberculin skin test conversions during anti-tumour necrosis factor therapy in patients with rheumatic diseases in a Mexican cohort. Ann Rheum Dis [Internet]. 2016;75((Fajardo Hermosillo L.D.) Rheumatology, IMSS, Guadalajara, Mexico):1010. Available from: https://www.embase.com/search/results?subaction=viewrecord&id=L612777177&from=export

107.	Favalli EG, Desiati F, Atzeni F, Sarzi-Puttini P, Caporali R, Pallavicini FB, et al. Serious infections during anti-TNFα treatment in rheumatoid arthritis patients. Autoimmun Rev [Internet]. 2009;8(3):266–73. Available from: https://www.embase.com/search/results?subaction=viewrecord&id=L50333574&from=export

108.	Ferrante M, Panés J, Baert F, Louis E, Kaser A, Gustafson D, et al. Long-term safety and efficacy of risankizumab treatment in patients with crohn’s disease: Interim results of the ongoing phase 2 open-label extension study. United Eur Gastroenterol J [Internet]. 2018;6(8):A122–3. Available from: https://www.embase.com/search/results?subaction=viewrecord&id=L625972434&from=export

109.	Fildan A, Toma C, Tofolean D, Arghir O, Dantes E. Tb risk in patients with biological therapy for psoriasis. Chest [Internet]. 2016;150(4):201A-. Available from: https://www.embase.com/search/results?subaction=viewrecord&id=L613468796&from=export

110.	Foeldvari I, Constantin T, Vojinovic J, Horneff G, Dehoorne J, Susic G, et al. An open-label extension study to assess the long-term safety of etanercept in pediatric patients with extended oligo, enthesitis related, and psoriatic juvenile idiopathic arthritis: 6-year data from the clipper studies. Arthritis Rheumatol [Internet]. 2017;69((Foeldvari I.; Constantin T.; Vojinovic J.; Horneff G.; Dehoorne J.; Susic G.; Kobusinska K.; Panaviene V.V.; Zuber Z.; Stanevicha V.; Chasnyk V.; Martini A.; Ruperto N.) Paediatric Rheumatology International Trials Organisation (PRINTO), Genoa, Italy(Ped). Available from: https://www.embase.com/search/results?subaction=viewrecord&id=L618916111&from=export

111.	Fortes FML, Sorte NB, Mariano VD, Andrade LD, Oliveira FA, Santos MCA, et al. Active tuberculosis in inflammatory bowel disease patients under treatment from an endemic area in Latin America. World J Gastroenterol [Internet]. 2020;26(44):6993–7004. Available from: https://www.embase.com/search/results?subaction=viewrecord&id=L2010268729&from=export

112.	Foster RA, Shah M, Hale M, Grant W, Abdulrehman A, Kuet KP, et al. Interferon   release assay (IGRA) testing in mycobacterium tuberculosis screening prior to anti-tnf treatment: experience across 3 medical specialities. Gut. 2011 Apr 1;60(Suppl 1):A144–5.

113.	Frolova X, Borisov S. Mycobacterium tuberculosis infection in patients with inflammatory bowel disease who received Anti-Tumor Necrosis Factor treatment in Russia. Eur Respir J [Internet]. 2020;56((Frolova X., ks.frolova@icloud.com) Moscow Research and Clinical Center for TB Control, Moscow Government Health Department, Moscow, Russian Federation(Borisov S.) Moscow Research and Clinical Center for TB Control, Moscow Government Health Department, Mo). Available from: https://www.embase.com/search/results?subaction=viewrecord&id=L633805646&from=export

114.	Fuchs I, Avnon L, Freud T, Abu-Shakra M. Repeated tuberculin skin testing following therapy with TNF-alpha inhibitors. Clin Rheumatol [Internet]. 2009;28(2):167–72. Available from: https://www.embase.com/search/results?subaction=viewrecord&id=L50272174&from=export

115.	Fuentes Coronel A, Fradejas Salazar P, Lopez Ramos C, Ochoa Sangrador C, Rascarachi G, Julian Gomez L, et al. USEFULNESS OF REPEATING TESTING FOR LATENT TUBERCULOSIS INFECTION IN PATIENTS WITH INFLAMMATORY BOWEL DISEASE (IBD). CORRELATION BETWEEN TUBERCULIN SKIN TEST (TST)/BOOSTER AND QUANTIFERON-TB (QFT). United Eur Gastroenterol J. 2017 Oct 29;5(5_suppl):P0350.

116.	Fujikawa A, Fujii T, Mimura S, Takahashi R, Sakai M, Suzuki S, et al. Tuberculosis Contact Investigation Using Interferon-Gamma Release Assay with Chest X-Ray and Computed Tomography. PLoS One. 2014 Jan 14;9(1):e85612.

117.	Gabriele F, Trachana M, Simitsopoulou M, Pratsidou-Gertsi P, Iosifidis E, Pana ZD, et al. Performance of QuantiFERON®-TB Gold In-Tube assay in children receiving disease modifying anti-rheumatic drugs. World J Pediatr [Internet]. 2017;13(5):472–8. Available from: https://www.embase.com/search/results?subaction=viewrecord&id=L616973651&from=export

118.	Gao Y, Zhang S, Shao LY, Qiu HM, Bai FM, He DY, et al. PP-211 Evaluation of latent Mycobacterium tuberculosis infection screening using TSPOT®. TB assay and TST in IMID patients prior to initiation of anti-TNF alpha therapy. Int J Infect Dis. 2011 Jul;15:S103.

119.	García-Gasalla M, Payeras-Cifre A, Cifuentes-Luna C, Navarro-Fernández V, Morey Torrandell C, Fernández-Baca V, et al. Use of Quantiferon-TB-Gold in Tube ® test for detecting latent tuberculosis in patients considered as candidates for anti-TNF therapy in routine clinical practice. Enferm infecc microbiol clín (Ed impr) [Internet]. 2013;31(2):76–81. Available from: https://pesquisa.bvsalud.org/portal/resource/pt/ibc-110420

120.	García-Sancho F MC, García-García L, Jiménez-Corona ME, Palacios-Martínez M, Ferreyra-Reyes LD, Canizales-Quintero S, et al. Is tuberculin skin testing useful to diagnose latent tuberculosis in BCG-vaccinated children? Int J Epidemiol. 2006 Dec 1;35(6):1447–54.

121.	Garcovich S, Ruggeri A, D’Agostino M, Ardito F, De Simone C, Delogu G, et al. Clinical applicability of Quantiferon-TB-Gold testing in psoriasis patients during long-term anti-TNF-alpha treatment: A prospective, observational study. J Eur Acad Dermatology Venereol [Internet]. 2012;26(12):1572–6. Available from: https://www.embase.com/search/results?subaction=viewrecord&id=L51653784&from=export

122.	Garziera G, Morsch ALB, Otesbelgue F, Staub FL, Palominos PE, Brenol C V, et al. Latent tuberculosis infection and tuberculosis in patients with rheumatic diseases treated with anti-tumor necrosis factor agents. Clin Rheumatol [Internet]. 2017;36(8):1891–6. Available from: https://www.embase.com/search/results?subaction=viewrecord&id=L616669841&from=export

123.	Gazel U, Kocakaya D, Topcu I, Karatas H, Karabacak M, Atagunduz P, et al. Is the risk of tuberculosis increased in behçet’s disease compared to other rheumatological disorders after anti-TNF-α Treatment. Arthritis Rheumatol [Internet]. 2019;71((Gazel U.) Marmara University, Faculty of Medicine, Department of Rheumatology, Istanbul, Turkey(Kocakaya D.) Marmara University, Faculty of Medicine, Department of Chest Diseases, Istanbul, Turkey(Topcu I.) Marmara University, School of Medicine, Istanbu):3029–30. Available from: https://www.embase.com/search/results?subaction=viewrecord&id=L633058512&from=export

124.	Gheorghe C, Dimitriu A, Gheorghe L. Latent tuberculosis infection in IBD patients receiving treatment with anti TNF-alfa agents. J Gastrointest Liver Dis [Internet]. 2019;28:9. Available from: https://www.embase.com/search/results?subaction=viewrecord&id=L632511320&from=export

125.	Girit S. Screening for Latent Tuberculosis in Children With Immune-Mediated Inflammatory Diseases Treated With Anti-Tumor Necrosis Factor Therapy: Comparison of Tuberculin Skin and T-SPOT Tuberculosis Tests. Arch Rheumatol. 2020 Mar 10;35(1):20–8.

126.	Gîrleanu I, Singeap A-M, Cojocariu C, Stoica O, Sfarti C, Trifan A. Efficacy of anti-TNF alpha therapy in patients receiving tuberculosis chemoprophylaxis. J Crohn’s Colitis [Internet]. 2013;7((Gîrleanu I.; Singeap A.-M.; Cojocariu C.; Stoica O.; Sfarti C.; Trifan A.) University of Medicine and Pharmacy Gr. T. Popa, Gastroenterology, Iasi, Romania):S216–7. Available from: https://www.embase.com/search/results?subaction=viewrecord&id=L70992045&from=export

127.	Gisondi P, Pezzolo E, Lo Cascio G, Girolomoni G. Latent tuberculosis infection in patients with chronic plaque psoriasis who are candidates for biological therapy. Br J Dermatol [Internet]. 2014;171(4):884–90. Available from: https://www.embase.com/search/results?subaction=viewrecord&id=L605657598&from=export

128.	Gogna A, Gulat S, Goel A. Tuberculosis in patients with rheumatc diseases and biological therapies: A single-center experience. Indian J Rheumatol [Internet]. 2018;13(6):S113-. Available from: https://www.embase.com/search/results?subaction=viewrecord&id=L626163731&from=export

129.	Gomes CMF, Terreri MT, Moraes-Pinto MI de, Barbosa C, Machado NP, Melo MR, et al. Incidence of active mycobacterial infections in Brazilian patients with chronic inflammatory arthritis and negative evaluation for latent tuberculosis infection at baseline - A longitudinal analysis after using TNFa blockers. Mem Inst Oswaldo Cruz. 2015 Nov;110(7):921–8.

130.	Gomes CMF, Terreri MT, Moraes-Pinto MI, Pinheiro MM. Performance of 4 methods for screening of latent tuberculosis infection in patients with chronic inflammatory arthritis under TNFα inhibitors: a 24-month prospective study. Adv Rheumatol. 2021 Dec 27;61(1):71.

131.	Gómez-Reino JJ, Carmona L, Rodríguez Valverde V, Mola EM, Montero MD. Treatment of rheumatoid arthritis with tumor necrosis factor inhibitors may predispose to significant increase in tuberculosis risk: A multicenter active-surveillance report. Arthritis Rheum [Internet]. 2003;48(8):2122–7. Available from: https://www.embase.com/search/results?subaction=viewrecord&id=L36959191&from=export

132.	González-Díaz V, Vázquez del Mercado M, Martín-Márquez B, Pérez-Gómez H, Morfin-Otero M, Rodríguez-Noriega E, et al. Efficiency of interferon-γ release assay for screening for latent tuberculosis in patients with systemic lupus erythematosus. Lupus. 2013;22(1):48.

133.	Greveson K, Murray C, Goodhand J, Cropley I, Murthy S, Hamilton M, et al. The use of commercial interferon-γ release assays to screen for mycobacterial infection in inflammatory bowel disease patients initiating anti-TNF agents. Gut [Internet]. 2012;61((Greveson K.; Murray C.; Goodhand J.; Hamilton M.) Centre for Gastroenterology, Royal Free Hospital, London, United Kingdom(Cropley I.) Infectious Diseases, Royal Free Hospital, London, United Kingdom(Murthy S.; Lipman M.) Respiratory Medicine, Royal Free):A223-. Available from: https://www.embase.com/search/results?subaction=viewrecord&id=L71122418&from=export

134.	Greveson K, Murray C, Toumpanakis C, Epstein O, Baptista L, Cropley I, et al. Screening patients initiating Anti-TNF agents for mycobacterial infection using interferon-gamma release assays (IGRA): The experience of a large inflammatory bowel disease service. Gastroenterology [Internet]. 2010;138(5):S526-. Available from: https://www.embase.com/search/results?subaction=viewrecord&id=L70397298&from=export

135.	Gudjónsdóttir MJ, Kötz K, Nielsen RS, Wilmar P, Olausson S, Wallmyr D, et al. Relation between BCG vaccine scar and an interferon-gamma release assay in immigrant children with “positive” tuberculin skin test (≥10 mm). BMC Infect Dis. 2016 Dec 6;16(1):540.

136.	Guidi L, Andrisani G, Papa A, Ardito F, Marzo M, Felice C, et al. Screening inflammatory bowel disease patients for latent tubercolosis in Italy: Comparison of quantiFERON-TB gold versus tuberculin skin test. Gastroenterology [Internet]. 2010;138(5):S526. Available from: https://www.embase.com/search/results?subaction=viewrecord&id=L70397299&from=export

137.	Hacioglu A, Borekci S, Melikoglu M, Ozguler Y, Esatoglu SN, Ugurlu S, et al. Screening for latent tuberculosis before starting TNF-alpha inhibitors in a population with high BCG vaccination rates. Rheumatol Int [Internet]. 2022;42(8):1443–51. Available from: https://www.embase.com/search/results?subaction=viewrecord&id=L2013079422&from=export

138.	Hakimian S, Popov Y, Rupawala A, Salomon-Escoto K, Hatch S, Pellish R. The conundrum of indeterminate QuantiFERON-TB Gold results before anti-tumor necrosis factor initiation. Biol Targets Ther. 2018 Feb;Volume 12:61–7.

139.	Hanta I, Ozbek S, Kuleci S, Kocabas A. The evaluation of latent tuberculosis in rheumatologic diseases for anti-TNF therapy: experience with 192 patients. Clin Rheumatol. 2008 Sep 5;27(9):1083–6.

140.	Hanta I, Ozbek S, Kuleci S, Seydaoglu G, Ozyilmaz E. Detection of latent tuberculosis infection in rheumatologic diseases before anti-TNFα therapy: tuberculin skin test versus IFN-γ assay. Rheumatol Int. 2012 Nov 18;32(11):3599–603.

141.	Hanta I, Ozbek S, Baydar O, Kuleci S. The effect of anti TNF-alpha therapy on tuberculin skin test reaction. Eur Respir J [Internet]. 2011;38. Available from: https://www.embase.com/search/results?subaction=viewrecord&id=L72117156&from=export

142.	Hatzara C, Hadziyannis E, Kandili A, Koutsianas C, Makris A, Georgiopoulos G, et al. Frequent conversion of tuberculosis screening tests during anti-tumour necrosis factor therapy in patients with rheumatic diseases. Ann Rheum Dis [Internet]. 2015;74(10):1848–53. Available from: https://www.embase.com/search/results?subaction=viewrecord&id=L606194421&from=export

143.	Havlucu Y, Kurhan F, Pirildar T, Goktalay T, Coskun AS, Celik P, et al. Pulmonary infections in patients receiving treatment of tumor necrosis factor alpha antagonists. Eur Respir J [Internet]. 2015;46((Havlucu Y.; Kurhan F.; Pirildar T.; Goktalay T.; Coskun A.S.; Celik P.; Yorgancioglu A.)). Available from: https://www.embase.com/search/results?subaction=viewrecord&id=L72108450&from=export

144.	He D, Bai F, Zhang S, Jiang T, Shen J, Zhu Q, et al. High incidence of tuberculosis infection in rheumatic diseases and impact for chemoprophylactic prevention of tuberculosis activation during biologics therapy. Clin Vaccine Immunol [Internet]. 2013;20(6):892–9. Available from: https://www.embase.com/search/results?subaction=viewrecord&id=L369009686&from=export

145.	Hejazi M-E, Ahmadzadeh A, Khabbazi A, Ebrahimi A, Farmani M, Hejazi Y. Tuberculin skin test conversion in patients under treatment with anti-tumor necrotizing factor alpha agents. BMC Infect Dis. 2020 Dec 2;20(1):464.

146.	Henriques A, Mourão F, Monteiro J, Espinheira MC, Pinto Pais I, Fernandes R, et al. Latent tuberculosis testing in paediatric patients with inflammatory bowel disease treated with anti-tumour necrosis factor agents: Do we need new testing recommendations? United Eur Gastroenterol J [Internet]. 2019;7(8):961. Available from: https://www.embase.com/search/results?subaction=viewrecord&id=L632137374&from=export

147.	Hernández M V, Descalzo MA, Cañete JD, Sanmarti R. When can biological therapy be resumed in patients with rheumatic conditions who develop tuberculosis infection during tumour necrosis factors antagonists therapy? study based on the biobadaser data registry. Arthritis Rheum [Internet]. 2012;64((Hernández M.V.; Cañete J.D.; Sanmarti R.) Hospital Clínic of Barcelona, Barcelona, Spain(Descalzo M.A.) Spanish Society of Rheumatology, Madrid, Spain):S701-. Available from: https://www.embase.com/search/results?subaction=viewrecord&id=L70999637&from=export

148.	Hizel K, Maral I, Karakus R, Aktas F. The influence of BCG immunisation on tuberculin reactivity and booster effect in adults in a country with a high prevalence of tuberculosis. Clin Microbiol Infect. 2004 Nov;10(11):981–4.

149.	Hofland RW, Thijsen SFT, Verhagen MAMT, Schenk Y, Bossink AWJ. Tuberculosis during TNF-α inhibitor therapy, despite screening: Table 1. Thorax. 2013 Nov;68(11):1079–80.

150.	Hong S, Kim YJ, Koo BS, Seo WJ, Kim Y, Lee C, et al. Resuming TNF antagonists in patients who developed tuberculosis. Int J Rheum Dis [Internet]. 2014;17((Hong S.; Kim Y.J.; Koo B.S.; Kim Y.; Lee C.; Yoo B.) Rheumatology, University of Ulsan College of Medicine Asan Medical Center, Seoul, South Korea(Seo W.J.) Rheumatology, Seoul Veterans Hospital, Seoul, South Korea):105. Available from: https://www.embase.com/search/results?subaction=viewrecord&id=L71477438&from=export

151.	Hong SN, Kim HJ, Kim KH, Han S ‐J., Ahn IM, Ahn HS. Risk of incident *Mycobacterium tuberculosis* infection in patients with inflammatory bowel disease: a nationwide population‐based study in South Korea. Aliment Pharmacol Ther. 2017 Jan 7;45(2):253–63.

152.	Horowitz HW, Luciano BB, Kadel JR, Wormser GP. Tuberculin skin test conversion in hospital employees vaccinated with bacille Calmette-Guérin: Recent Mycobacterium tuberculosis infection or booster effect? Am J Infect Control. 1995 Jun;23(3):181–7.

153.	Hou JK, Kramer JR, Richardson P, Sansgiry S, El-Serag HB. Tuberculosis Screening and Reactivation among a National Cohort of Patients with Inflammatory Bowel Disease Treated with Tumor Necrosis Factor Alpha Antagonists. Inflamm Bowel Dis [Internet]. 2017;23(2):254–60. Available from: https://www.embase.com/search/results?subaction=viewrecord&id=L613789565&from=export

154.	Hradsky O, Ohem J, Zarubova K, Mitrova K, Durilova M, Kotalova R, et al. Disease activity is an important factor for indeterminate interferon-γ release assay results in children with inflammatory bowel disease. J Pediatr Gastroenterol Nutr [Internet]. 2014;58(3):320–4. Available from: https://www.embase.com/search/results?subaction=viewrecord&id=L52812532&from=export

155.	Hsia EC, Schluger N, Cush JJ, Chaisson RE, Matteson EL, Xu S, et al. Interferon-γ release assay versus tuberculin skin test prior to treatment with golimumab, a human anti-tumor necrosis factor antibody, in patients with rheumatoid arthritis, psoriatic arthritis, or ankylosing spondylitis. Arthritis Rheum [Internet]. 2012;64(7):2068–77. Available from: https://www.embase.com/search/results?subaction=viewrecord&id=L365143154&from=export

156.	Hsia EC, Cush JJ, Matteson EL, Beutler A, Doyle MK, Hsu B, et al. Comprehensive tuberculosis screening program in patients with inflammatory arthritides treated with golimumab, a human anti-tumor necrosis factor antibody, in phase III clinical trials. Arthritis Care Res [Internet]. 2013;65(2):309–13. Available from: https://www.embase.com/search/results?subaction=viewrecord&id=L368242075&from=export

157.	Hsiao C-Y, Chiu H-Y, Wang T-S, Tsai T-F. Serial QuantiFERON-TB Gold testing in patients with psoriasis treated with ustekinumab. PLoS One. 2017 Sep 8;12(9):e0184178.

158.	Hsieh T-Y, Chen Y-M, Chen H-H, Chen Y-R, Hung W-T, Chen Y-J, et al. Positive quantiferon conversion might be related to prior anti-TNF treatment in patients with psoriatic arthritis and psoriasis treated with ustekinumab: A cross-specialty, real-world study. Int J Rheum Dis [Internet]. 2018;21((Hsieh T.-Y.; Chen Y.-M.; Chen H.-H.; Hung W.-T.; Chen Y.-J.; Hsieh C.-W.; Huang W.-N.; Chen Y.-H.; Lin C.-T.; Lai K.-L.; Chen Y.-H.) Division of Allergy, Immunology and Rheumatology, Taichung Veterans General Hospital, Taichung, Taiwan(Chen Y.-R.) Divisi):89. Available from: https://www.embase.com/search/results?subaction=viewrecord&id=L624303799&from=export

159.	Huang S-F, Chen M-H, Wang F-D, Tsai C-Y, Fung C-P, Su W-J. Efficacy of isoniazid salvage therapy for latent tuberculosis infection in patients with immune-mediated inflammatory disorders – A retrospective cohort study in Taiwan. J Microbiol Immunol Infect. 2018 Dec;51(6):784–93.

160.	Husni E, Kavanaugh A, Nantel F, Harrison D, Kim L, Lo K, et al. Intravenous golimumab in adult patients with active psoriatic arthritis: Efficacy and safety through 1 year. J Rheumatol [Internet]. 2018;45(7):994. Available from: https://www.embase.com/search/results?subaction=viewrecord&id=L623151722&from=export

161.	Iba A, Tomio J, Yamana H, Sugiyama T, Yoshiyama T, Kobayashi Y. Tuberculosis screening and management of latent tuberculosis infection prior to biologic treatment in patients with immune-mediated inflammatory diseases: A longitudinal population-based analysis using claims data. Heal Sci Reports [Internet]. 2020;3(4). Available from: https://www.embase.com/search/results?subaction=viewrecord&id=L2007589232&from=export

162.	Igari H, Ishikawa S, Tsuyuzaki M, Suzuki K, Matsumura R. The analysis of quantiferon-TB gold plus (4th generation QFT) in comparison with TSPOT in rheumatoid arthritis for latent tuberculosis infection. Am J Respir Crit Care Med. 2017;195.

163.	Igari H, Matsumura R, Nakazawa T, Ishikawa S, Yamagishi F. The utility of interferon gamma release assays in rheumatoid arthritis for latent tuberculosis infection comparison between quantiferon-TB git and T-Spot.TB. Am J Respir Crit Care Med [Internet]. 2015;191((Igari H.) Chiba University Hospital, Chiba, Japan(Igari H.; Matsumura R.; Nakazawa T.; Ishikawa S.; Yamagishi F.) National Hospital Organization, Chiba-East Hospital, Chiba, Japan). Available from: https://www.embase.com/search/results?subaction=viewrecord&id=L72053339&from=export

164.	Ines M, Besma H, Sabrine L, Sarra A, Emna BJ, Jamel A, et al. Evaluation of Tuberculin skin test size and risk of tuberculosis in children household contact. In: Tuberculosis. European Respiratory Society; 2018. p. PA2737.

165.	Jacobs S, Warman A, Richardson R, Yacoub W, Lau A, Whittaker D, et al. The Tuberculin Skin Test Is Unreliable in School Children BCG-vaccinated in Infancy and at Low Risk of Tuberculosis Infection. Pediatr Infect Dis J. 2011 Sep;30(9):754–8.

166.	Jauregui-Amezaga A, Turon F, Ordás I, Gallego M, Feu F, Ricart E, et al. Risk of developing tuberculosis under anti-TNF treatment despite latent infection screening. J Crohn’s Colitis [Internet]. 2013;7(3):208–12. Available from: https://www.embase.com/search/results?subaction=viewrecord&id=L52046416&from=export

167.	Jeong DH, Kang J, Jung YJ, Yoo B, Lee C-K, Kim Y-G, et al. Comparison of latent tuberculosis infection screening strategies before tumor necrosis factor inhibitor treatment in inflammatory arthritis: IGRA-alone versus combination of TST and IGRA. PLoS One. 2018 Jul 5;13(7):e0198756.

168.	Jo K-W, Hong YK, Jung YJ, Lee S-D, Kim WS, Kim DS, et al. Incidence of tuberculosis among anti-TNF users in patients with previous history of tuberculosis. Eur Respir J [Internet]. 2013;42((Jo K.-W., heathcliff6800@hanmail.net; Jung Y.J., tazo76@naver.com; Lee S.-D., sdlee@amc.seoul.kr; Kim W.S., wskim2@amc.seoul.kr; Kim D.S., dskim@amc.seoul.kr; Shim T.S., shimts@amc.seoul.kr) Department of Pulmonary and Critical Care Medicine, Asan Medica). Available from: https://www.embase.com/search/results?subaction=viewrecord&id=L71843992&from=export

169.	Joza K, Gallego C, Blumenfeld A, Mancini P, Muñoz L, Pinelli C, et al. Incidencia de infección tuberculosa latente en un estudio de contactos escolares comparando dos puntos de corte de la prueba de tuberculina. Rev Am Med Respir. 2020;20(4):358–63.

170.	Julian AN, López JA, Calzada-Hernández J, Cuadros EN, Peña MJM, Carpi FJM. Diagnosis of tuberculosis infection in pediatric patients treated with inhibitors of the tumour necrosis factor alpha. A multicenter national study comparing tuberculin skin test and igra tests. Pediatr Rheumatol [Internet]. 2014;12. Available from: https://www.embase.com/search/results?subaction=viewrecord&id=L71686979&from=export

171.	Jung H-J, Kim T-J, Kim H-S, Cho Y-N, Jin H-M, Kim M-J, et al. Analysis of predictors influencing indeterminate whole-blood interferon-gamma release assay results in patients with rheumatic diseases. Rheumatol Int [Internet]. 2014;34(12):1711–20. Available from: https://www.embase.com/search/results?subaction=viewrecord&id=L53157750&from=export

172.	Jung SM, Ju JH, Park M-S, Kwok S-K, Park K-S, Kim H-Y, et al. Risk of tuberculosis in patients treated with anti-tumor necrosis factor therapy: A nationwide study in South Korea, a country with an intermediate tuberculosis burden. Int J Rheum Dis [Internet]. 2015;18(3):323–30. Available from: https://www.embase.com/search/results?subaction=viewrecord&id=L604058326&from=export

173.	Jung YJ, Lee JY, Jo K-W, Yoo B, Lee C-K, Kim Y-G, et al. The ‘either test positive’’ strategy for latent tuberculous infection before anti-tumour necrosis factor treatment.’ Int J Tuberc Lung Dis. 2014 Apr 1;18(4):428–34.

174.	Jung YJ, Woo HI, Jeon K, Koh W-J, Jang DK, Cha HS, et al. The Significance of Sensitive Interferon Gamma Release Assays for Diagnosis of Latent Tuberculosis Infection in Patients Receiving Tumor Necrosis Factor-α Antagonist Therapy. PLoS One. 2015 Oct 16;10(10):e0141033.

175.	Kamat N, Kedia S, Ghoshal UC, Makharia G, Sood A, Midha V, et al. Effectiveness and safety of Adalimumab biosimilar in inflammatory bowel disease: A multi-center study. Indian J Gastroenterol [Internet]. 2018;37((Kamat N.; Kedia S.; Makharia G.; Ahuja V., vineet.aiims@gmail.com) Departments of Gastroenterology, All India Institute of Medical Sciences, New Delhi, India(Ghoshal U.C.) Department of Gastroenterology, Sanjay Gandhi Postgraduate Institute of Medical Sc):A44–5. Available from: https://www.embase.com/search/results?subaction=viewrecord&id=L625623893&from=export

176.	Kaneko S, Tsuruta N, Yamaguchi K, Miyagi T, Takahashi K, Higashi Y, et al. Mycobacterium tuberculosis</i> infection in psoriatic patients treated with biologics: Real‐world data from 18 Japanese facilities. J Dermatol. 2020 Feb 24;47(2):128–32.

177.	Kang J, Jeong DH, Han M, Yang S-K, Byeon J-S, Ye BD, et al. Incidence of Active Tuberculosis within One Year after Tumor Necrosis Factor Inhibitor Treatment according to Latent Tuberculosis Infection Status in Patients with Inflammatory Bowel Disease. J Korean Med Sci. 2018;33(47).

178.	Kaptan Y, Suner A, Taş MN, Oksel F, Aksu K, Sayiner A. Tuberculosis despite latent infection screening and treatment in patients receiving TNF inhibitor therapy. Clin Rheumatol. 2021 Sep 20;40(9):3783–8.

179.	Karataş Toğral A, Muştu Koryürek Ö, Şahin M, Bulut C, Yağci S, Ekşioğlu HM. Association of clinical properties and compatibility of the <scp>Q</scp> uanti <scp>FERON</scp> ‐ <scp>TB G</scp> old <scp>I</scp> n‐ <scp>T</scp> ube test with the tuberculin skin test in patients with psoriasis. Int J Dermatol. 2016 Jun 24;55(6):629–33.

180.	Kaur M, Singapura P, Kalakota N, Cruz G, Shukla R, Ahsan S, et al. Factors That Contribute to Indeterminate Results From the QuantiFERON-TB Gold In-Tube Test in Patients With Inflammatory Bowel Disease. Clin Gastroenterol Hepatol. 2018 Oct;16(10):1616-1621.e1.

181.	Kay J, Fleischmann RM, Keystone EC, Rahman MU, Hsia EC, Doyle MK, et al. Safety of Golimumab, a new human anti-TNF antibody, in rheumatoid arthritis, psoriatic arthritis, and ankylosing spondylitis: An analysis of pooled data From randomized, double-blind, placebo-controlled studies. Arthritis Rheum [Internet]. 2009;60((Kay J.; Fleischmann R.M.; Keystone E.C.; Rahman M.U.; Hsia E.C.; Doyle M.K.; Hsu B.) University of Massachusetts Memorial, Medical Center, Worcester, MA, United States(Kay J.; Fleischmann R.M.; Keystone E.C.; Rahman M.U.; Hsia E.C.; Doyle M.K.; Hsu B.) M):790. Available from: https://www.embase.com/search/results?subaction=viewrecord&id=L70374468&from=export

182.	Ke W-M, Chen L-S, Parng I-M, Chen W-W, On AWF. Risk of tuberculosis in rheumatoid arthritis patients on tumour necrosis factor-alpha inhibitor treatment in Taiwan. Int J Tuberc Lung Dis [Internet]. 2013;17(12):1590-1595+i. Available from: https://www.embase.com/search/results?subaction=viewrecord&id=L370317342&from=export

183.	Khraishi M, Nüßlein H, Lorenz H, Alten R, Bensen W, Bessette L, et al. Efficacy and safety of abatacept treatment for rheumatoid arthritis in a real-life setting in European and Canadian populations: A 6-month interim analysis of the ACTION study. J Rheumatol [Internet]. 2012;39(8):1728. Available from: https://www.embase.com/search/results?subaction=viewrecord&id=L70873078&from=export

184.	Kilinc AA, Onal P, Oztosun B, Yildiz M, Adrovic A, Sahin S, et al. Determination of tuberculin skin test for isoniazid prophylaxis in BCG vaccinated children who are using anti‐TNF agents for rheumatologic diseases. Pediatr Pulmonol. 2020 Oct 23;55(10):2689–96.

185.	Kim E-M, Uhm W-S, Bae S-C, Yoo D-H, Kim T-H. Incidence of tuberculosis among Korean patients with ankylosing spondylitis who are taking tumor necrosis factor blockers. J Rheumatol [Internet]. 2011;38(10):2218–23. Available from: https://www.embase.com/search/results?subaction=viewrecord&id=L362693905&from=export

186.	Kim E-M, Choi C-B, Sung Y-K, Uhm W-S, Jun J-B, Kim T-H, et al. Incidence of tuberculosis in Korean patients with ankylosing spondylitis treated with tumor necrosis factor blockers. Arthritis Rheum [Internet]. 2010;62((Kim E.-M.; Choi C.-B.; Sung Y.-K.; Uhm W.-S.; Jun J.-B.; Kim T.-H.; Bae S.-C.) Hanyang University, Hospital for Rheumatic Diseases, Seoul, South Korea):1941. Available from: https://www.embase.com/search/results?subaction=viewrecord&id=L70381223&from=export

187.	Kim ES, Song GA, Cho KB, Park KS, Kim KO, Jang BI, et al. Significant risk and associated factors of active tuberculosis infection in Korean patients with inflammatory bowel disease using anti-TNF agents. World J Gastroenterol [Internet]. 2015;21(11):3308–16. Available from: https://www.embase.com/search/results?subaction=viewrecord&id=L603429374&from=export

188.	Kim H-C, Jo K-W, Jung YJ, Yoo B, Lee C-K, Kim Y-G, et al. Diagnosis of latent tuberculosis infection before initiation of anti-tumor necrosis factor therapy using both tuberculin skin test and QuantiFERON-TB Gold In Tube assay. Scand J Infect Dis. 2014 Nov 8;46(11):763–9.

189.	Kim HW, Park JK, Yang J-A, Yoon YI, Lee EY, Song YW, et al. Comparison of tuberculosis incidence in ankylosing spondylitis and rheumatoid arthritis during tumor necrosis factor inhibitor treatment in an intermediate burden area. Clin Rheumatol. 2014 Sep 22;33(9):1307–12.

190.	Kim HW, Kwon OC, Han SH, Park M-C. Positive conversion of interferon-γ release assay in patients with rheumatic diseases treated with biologics. Rheumatol Int. 2020 Mar 9;40(3):471–9.

191.	Kim J, Im JP, Yim J-J, Lee CK, Park D Il, Eun CS, et al. Clinical Features and Outcomes of Tuberculosis in Inflammatory Bowel Disease Patients Treated with Anti-tumor Necrosis Factor Therapy. Korean J Gastroenterol. 2020;75(1):29.

192.	Kim J, Won S, Choi C, Sung Y, Song GG, Bae S. Evaluation of the usefulness of interferon‐gamma release assays and the tuberculin skin test for the detection of latent *Mycobacterium tuberculosis* infections in Korean rheumatic patients who are candidates for biologic agents. Int J Rheum Dis. 2015 Mar 28;18(3):315–22.

193.	Kim J-H, Cho S-K, Han M, Choi C-B, Kim T-H, Jun J-B, et al. Factors Influencing Discrepancies Between the QuantiFERON-TB Gold in Tube Test and the Tuberculin Skin Test in Korean Patients with Rheumatic Diseases. Semin Arthritis Rheum. 2013 Feb;42(4):424–32.

194.	Kyeong-Hee KIM, Sung-Won LEE, CHUNG W-T, Byoung-Gwon KIM, Kwang-Sook WOO, Jin-Yeong HAN, et al. Serial Interferon-gamma Release Assays for the Diagnosis of Latent Tuberculosis Infection in Patients Treated with Immunosuppressive Agents. Korean J Lab Med [Internet]. 2011;271–8. Available from: http://dx.doi.org/10.3343/kjlm.2011.31.4.271

195.	Kimball AB, Pariser D, Kricorian G, Gelfand JM. Final results from OBSERVE-5, a 5-year observational registry of patients with moderate to severe plaque psoriasis receiving etanercept. J Am Acad Dermatol [Internet]. 2014;70(5):AB170-. Available from: https://www.embase.com/search/results?subaction=viewrecord&id=L71390764&from=export

196.	Kimball AB, Kerdel F, Adams D, Mrowietz U, Gelfand JM, Gniadecki R, et al. Adalimumab for the treatment of moderate to severe hidradenitis suppurativa: A parallel randomized trial. Ann Intern Med [Internet]. 2012;157(12):846–55. Available from: https://www.embase.com/search/results?subaction=viewrecord&id=L366301713&from=export

197.	Kiwanuka JP. Interpretation of tuberculin skin-test results in the diagnosis of tuberculosis in children. Afr Health Sci. 2005 Jun;5(2):152–6.

198.	Kleinert S, Tony H-P, Krueger K, Detert J, Mielke F, Rockwitz K, et al. Screening for latent tuberculosis infection: performance of tuberculin skin test and interferon-γ release assays under real-life conditions. Ann Rheum Dis. 2012 Nov;71(11):1791–5.

199.	Koike T, Harigai M, Inokuma S, Ishiguro N, Ryu J, Takeuchi T, et al. Post-marketing surveillance program of tocilizumab for RA in Japan interim analyses of 3,881 patients. Arthritis Rheum [Internet]. 2010;62((Koike T.) U Occupa and Environ. Hlth., Kitakyushu, Japan(Inokuma S.) Hokkaido University, Japan(Takei S.) Japanese Red Cross Medical Center, Japan(Takeuchi T.) Kagoshima University, Kagoshima City, Japan(Ishiguro N.) Keio University, Toyko, Japan(Ryu J.)):399. Available from: https://www.embase.com/search/results?subaction=viewrecord&id=L70381639&from=export

200.	Koo BS, Lim Y-C, Lee M-Y, Jeon J-Y, Yoo H-J, Oh I-S, et al. The risk factors and incidence of major infectious diseases in patients with ankylosing spondylitis receiving tumor necrosis factor inhibitors. Mod Rheumatol [Internet]. 2021;31(6):1192–201. Available from: https://www.embase.com/search/results?subaction=viewrecord&id=L2010498660&from=export

201.	Koo BS, So MW, Seo WJ, Kim Y-G, Lee C-K, Yoo B. Reactivation of latent tuberculosis infection and new infection of tuberculosis in patients with anti-tumor necrosis factor therapy for tuberculosis-endemic area. Ann Rheum Dis. 2013 Jun 23;71(Suppl 3):512.1-512.

202.	Kruczak K, Skucha W, Duplaga M, Sanak M, Nizankowska-Mogilnicka E. Quantiferon-Tbgit (QFT) vs tuberculosis skin test (TST) in diagnosis of latent tuberculosis (Tb) infection (LTBI) in patients treated by oral glucocorticosteroids. Am J Respir Crit Care Med [Internet]. 2010;181(1). Available from: https://www.embase.com/search/results?subaction=viewrecord&id=L70842228&from=export

203.	Kumar P, Vuyyuru SK, Kante B, Kedia S, Sahu P, Ranjan MK, et al. Efficacy and safety of biosimilar versus originator infliximab in patients with inflammatory bowel disease: A real-world cohort analysis. Indian J Gastroenterol [Internet]. 2022;41(5):446–55. Available from: https://www.embase.com/search/results?subaction=viewrecord&id=L2020117919&from=export

204.	Kurt OK, Kurt B, Talay F, Tug T, Soy M, Bes C, et al. Intermediate to long-term follow-up results of INH chemoprophylaxis prior to anti-TNF-alpha therapy in a high-risk area for tuberculosis. Wien Klin Wochenschr. 2013 Oct 6;125(19–20):616–20.

205.	Kurtz T, Feil AC, Nascimento LS, de Oliveira Abreu P, Scotta MC, Pinto LA. Effect of neonatal bacille Calmette-Guérin on the tuberculin skin test reaction in the first 2 years of life. Int J Tuberc Lung Dis. 2019 Mar 1;23(3):344–8.

206.	Kwon M, Sung M, Kwon Y-J, Song YG, Lee S-W, Park M-C, et al. Active Tuberculosis Risk With Tumor Necrosis Factor Inhibitors After Treating Latent Tuberculosis. JCR J Clin Rheumatol. 2014 Mar;20(2):68–73.

207.	Lai H-C, Cheng K-S, Chang C-H, Feng C-L, Chen T-W, Chou J-W. Latent tuberculosis and active tuberculosis infection in patients with inflammatory bowel disease treating by biological agents: An experience of a medical centre in Taiwan. J Crohn’s Colitis [Internet]. 2019;13((Lai H.-C.) China Medical University Hospital, Department of Chinese Medicine, Taichung, Taiwan(Cheng K.-S.; Chang C.-H.; Feng C.-L.; Chou J.-W.) China Medical University Hospital, Division of Gastroenterology and Hepatology, Taichung, Taiwan(Cheng K.-S.;):S548-. Available from: https://www.embase.com/search/results?subaction=viewrecord&id=L628297655&from=export

208.	Laurindo I, Ribeiro ACM, Moraes JCB, Saad CGS, Bonfiglioli KR, Souza FHC, et al. Latent tuberculosis screening and treatment in rheumatoid arthritis patients eligible for anti-TNF therapy in endemic areas: Does it work? Arthritis Rheum [Internet]. 2012;64((Laurindo I.; Ribeiro A.C.M.; Moraes J.C.B.; Saad C.G.S.; Bonfiglioli K.R.; Souza F.H.C.; Calich A.L.G.; Waisberg M.G.; Guedes L.K.N.; Bonfa E.) Faculdade de Medicina, Universidade de São Paulo, São Paulo, Brazil):S75-. Available from: https://www.embase.com/search/results?subaction=viewrecord&id=L70998172&from=export

209.	Lawrance IC, Bampton PA, Sparrow M, Gearry RB, Leong RW, Andrews JM, et al. Serious infective complications in anti-TNFα Treated Inflammatory Bowel Disease Patients. Gastroenterology [Internet]. 2009;136(5):A189-. Available from: https://www.embase.com/search/results?subaction=viewrecord&id=L70151433&from=export

210.	Lee CK, Wong SH V, Lui G, Tang W, Tam LS, Ip M, et al. A Prospective Study to Monitor for Tuberculosis During Anti-tumour Necrosis Factor Therapy in Patients With Inflammatory Bowel Disease and Immune-mediated Inflammatory Diseases. J Crohns Colitis. 2018;12(8):954–62.

211.	Lee EH, Kang YA, Leem AY, Park MS, Kim YS, Kim SK, et al. Active Tuberculosis Incidence and Characteristics in Patients Treated with Tumor Necrosis Factor Antagonists According to Latent Tuberculosis Infection. Sci Rep. 2017;7(1):6473.

212.	Lee H, Park HY, Jeon K, Jeong B-H, Hwang J-W, Lee J, et al. QuantiFERON-TB Gold In-Tube Assay for Screening Arthritis Patients for Latent Tuberculosis Infection before Starting Anti-Tumor Necrosis Factor Treatment. PLoS One. 2015 Mar 6;10(3):e0119260.

213.	Lee HW, Lee YJ, Kim SJ, Park JS, Cho Y-J, Yoon H Il, et al. Comparing tuberculin skin test and interferon γ release assay (T-SPOT.TB) to diagnose latent tuberculosis infection in household contacts. Korean J Intern Med. 2017 May 1;32(3):486–96.

214.	Lee J, Kim E, Jang EJ, Lee C-H, Lee EY, Im JP, et al. Efficacy of Treatment for Latent Tuberculosis in Patients Undergoing Treatment with a Tumor Necrosis Factor Antagonist. Ann Am Thorac Soc. 2017 May;14(5):690–7.

215.	Lee J, Joo SH, Song YW. PREVENTION OF ACTIVE TUBERCULOSIS INFECTION USING INTERFERON GAMMA RELEASING ASSAY (IGRA) IN PATIENTS WITH RHEUMATOID ARTHRITIS UNDERGOING TUMOR NECROSIS FACTOR-ALPHA INHIBITORS TREATMENT. In: Poster Presentations. BMJ Publishing Group Ltd and European League Against Rheumatism; 2019. p. 604.1-604.

216.	Lee JY, Oh K, Hong HS, Kim K, Hong SW, Park JH, et al. Risk and characteristics of tuberculosis after anti-tumor necrosis factor therapy for inflammatory bowel disease: a hospital-based cohort study from Korea. BMC Gastroenterol. 2021 Dec 20;21(1):390.

217.	Lee SJ, Baek K, Lee YJ, Lee S, Kim H, Lee SG, et al. A large pooled safety analysis of 3 post-marketing studies conducted in inflammatory bowel disease patients treated with biosimilar infliximab (CT-P13). United Eur Gastroenterol J [Internet]. 2019;7(8):363. Available from: https://www.embase.com/search/results?subaction=viewrecord&id=L632137361&from=export

218.	Lee SW, Oh SY, Lee JB, Choi CM, Kim HJ. Tuberculin Skin Test Distribution following a Change in BCG Vaccination Policy. PLoS One. 2014 Jan 23;9(1):e86419.

219.	Li H, Xin H, Qian S, Li X, Zhang H, Li M, et al. Testing of tuberculosis infection among Chinese adolescents born after terminating the Bacillus Calmette–Guérin booster vaccination: subgroup analysis of a population-based cross-sectional study. Front Med. 2017 Dec 3;11(4):528–35.

220.	Liao T-L, Lin C-H, Chen Y-M, Chang C-L, Chen H-H, Chen D-Y. Different Risk of Tuberculosis and Efficacy of Isoniazid Prophylaxis in Rheumatoid Arthritis Patients with Biologic Therapy: A Nationwide Retrospective Cohort Study in Taiwan. PLoS One. 2016 Apr 11;11(4):e0153217.

221.	Lim CH, Chen H-H, Chen Y-H, Chen D-Y, Huang W-N, Tsai J-J, et al. The risk of tuberculosis disease in rheumatoid arthritis patients on biologics and targeted therapy: A 15-year real world experience in Taiwan. PLoS One. 2017 Jun 1;12(6):e0178035.

222.	Lim C-H, Lin C-H, Chen D-Y, Chen Y-M, Chao W-C, Liao T-L, et al. One-Year Tuberculosis Risk in Rheumatoid Arthritis Patients Starting Their First Tumor Necrosis Factor Inhibitor Therapy from 2008 to 2012 in Taiwan: A Nationwide Population-Based Cohort Study. PLoS One. 2016 Nov 10;11(11):e0166339.

223.	Lima C, Garcia K, Facas B, Azevedo M, Queiroz N, Barros L, et al. Active tuberculosis infection in patients with inflammatory bowel disease followed in a referral center. Am J Gastroenterol [Internet]. 2019;114((Lima C.) Hospital Das Clinicas, Faculdade De Medicina Da Cidade De São Paulo, São Paulo, Brazil(Garcia K.; Facas B.; Gasparini R.) Faculdade De Medicina, Universidade De São Paulo, São Paulo, Brazil(Azevedo M.; Barros L.; Alexandre C.; Milani L.; Damião):S20-. Available from: https://www.embase.com/search/results?subaction=viewrecord&id=L630874337&from=export

224.	Lima EV de A. Avaliação do teste T-SPOT.TB no diagnóstico de infecção tuberculosa latente em pacientes com psoríase. [São Paulo]: Universidade de São Paulo; 2010.

225.	Logarajah V, Liwanag J, Chiou FK. Prevalence, management and outcome of mycobacterium tuberculosis infections in paediatric patients with inflammatory bowel disease in Singapore. J Pediatr Gastroenterol Nutr [Internet]. 2019;69((Logarajah V.; Liwanag J.; Chiou F.K.) KK Women’s and Children’s Hospital, Paediatric Gastroenterology, Hepatology and Nutrition, Singapore, Singapore):141. Available from: https://www.embase.com/search/results?subaction=viewrecord&id=L629585318&from=export

226.	Long W, Cai F, Wang X, Zheng N, Wu R. High risk of activation of latent tuberculosis infection in rheumatic disease patients. Infect Dis (Auckl) [Internet]. 2020;52(2):80–6. Available from: https://www.embase.com/search/results?subaction=viewrecord&id=L2003520405&from=export

227.	Lopes DM de A, Pinheiro VGF, Monteiro HSA, Queiroz JAN, Madeira L dos S, Lopes MM de A. Diagnóstico e tratamento da tuberculose latente em pacientes com doenças inflamatórias crônicas e uso de imunobiológicos inibidores do TNF-α. J Bras Pneumol. 2011 Jun;37(3):308–16.

228.	Lopes DM de A, Pinheiro VGF, Monteiro HSA. Diagnosis and treatment of latent tuberculosis infection in patients undergoing treatment with immunobiologic agents: a four-year experience in an endemic area. J Bras Pneumol. 2019;45(6).

229.	Lorenzetti R, Zullo A, Hassan C, Campo S, Bruzzese V. Risk of tuberculosis following anti-TNF therapy: Pooled-data analysis of randomized controlled trials on IBD, rheumatic diseases, and psoriasis. Dig Liver Dis [Internet]. 2013;45((Lorenzetti R.; Zullo A.; Hassan C.; Campo S.; Bruzzese V.) UOC di Gastroenterologia PTP Nuovo Regina Margherita, Roma, Italy):S147-. Available from: https://www.embase.com/search/results?subaction=viewrecord&id=L71037328&from=export

230.	Losi M, Meacci M, Ferrari D, D’Amico R, Giuggioli D, Luppi F, et al. Interferon-gamma release assays for the diagnosis of latent tuberculosis infection in patients with rheumatic diseases. Clin Microbiol Infect [Internet]. 2010;16((Losi M.; Meacci M.; Ferrari D.; D’Amico R.; Giuggioli D.; Luppi F.; Meccugni B.; Manzini C.; Ferri C.; Roversi P.; Richeldi L.; Rumpianesi F.) Modena, Italy):S543-. Available from: https://www.embase.com/search/results?subaction=viewrecord&id=L70196443&from=export

231.	Lupaşcu A, Chiosa A-M, Popa I, Popa RC, Mihai C, Prelipcean CC. The frequency of adverse events during anti-TNF therapy in patients with inflammatory bowel disease. J Gastrointest Liver Dis [Internet]. 2018;27((Lupaşcu A.; Chiosa A.-M.; Popa I.; Popa R.C.; Mihai C.; Prelipcean C.C.)):13–4. Available from: https://www.embase.com/search/results?subaction=viewrecord&id=L632511585&from=export

232.	Machado J, Ministro P, Araújo R, Cancela E, Castanheira A, Silva A. Diagnosis of latent tuberculosis in patients with inflammatory bowel disease: Prospective comparison between tuberculin skin test and IGRA test QuantiFERON TB-Gold®. J Crohn’s Colitis [Internet]. 2011;5(1):S64. Available from: https://www.embase.com/search/results?subaction=viewrecord&id=L70476878&from=export

233.	Mahmoud I, Dridi L, Saidane O, Sahli H, Tekaya R, Abdelmoula L, et al. Latent tuberculosis screening and management before biologic agents instauration and risk of reactivation: The experience of an endemic tuberculosis country. Ann Rheum Dis [Internet]. 2013;72((Mahmoud I.; Dridi L.; Saidane O.; Sahli H.; Tekaya R.; Abdelmoula L.; Chaabouni L.; Zouari R.) Rheumatology, Charles Nicolle Hospital, Tunis, Tunisia). Available from: https://www.embase.com/search/results?subaction=viewrecord&id=L71334786&from=export

234.	Makay B, Gucenmez OA, Çaǧlar I, Bayram SN, Gülez N, Devrim I. Latent tuberculosis infection in children with pediatric rheumatologic diseases treated with canakinumab. Ann Rheum Dis [Internet]. 2019;78((Makay B.; Gucenmez O.A.; Gülez N.) Dr. Behçet Uz Children’s Hospital, Department of Pediatric Rheumatology, Izmir, Turkey(Çaǧlar I.; Bayram S.N.; Devrim I.) Dr. Behçet Uz Children’s Hospital, Department of Pediatric Infectious Diseases, Izmir, Turkey):561–2. Available from: https://www.embase.com/search/results?subaction=viewrecord&id=L628843712&from=export

235.	Malaviya AN, Rawat R, Sadhana, Baghel S, Agarwal D, Sharma A, et al. Recommended strategy for the screening of tuberculosis infection (TBI) and latent TB infection (LTBI) among patients receiving anti-tumor necrosis factor-A (ITNF) is highly successful. Indian J Rheumatol [Internet]. 2010;5(3):S6-. Available from: https://www.embase.com/search/results?subaction=viewrecord&id=L70312708&from=export

236.	Malaviya A, Thakaran R, Rawat R, Kapoor S, Garg S, Baghel S, et al. Real life experience of a screening strategy for latent tuberculosis before treatment with biologicals in indian patients with rheumatic diseases. Indian J Rheumatol. 2018;13(4):233.

237.	Malinová J, Hájková M, Hatalová A, Šteňová E. Risk of latent tuberculosis in the cohort of patients with rheumatoid arthritis in Slovakia. Epidemiol Mikrobiol Imunol [Internet]. 2021;70(2):83–90. Available from: https://www.embase.com/search/results?subaction=viewrecord&id=L2014937475&from=export

238.	Manadan AM, Joyce K, Sequeira W, Block JA. Etanercept therapy in patients with a positive tuberculin skin test. Clin Exp Rheumatol [Internet]. 2007;25(5):743–5. Available from: https://www.embase.com/search/results?subaction=viewrecord&id=L350284848&from=export

239.	Mancuso JD, Mody RM, Olsen CH, Harrison LH, Santosham M, Aronson NE. The Long-term Effect of Bacille Calmette-Guérin Vaccination on Tuberculin Skin Testing. Chest. 2017 Aug;152(2):282–94.

240.	Mankia S, Peters JE, Kang S, Moore S, Ehrenstein MR. Tuberculosis and anti-TNF treatment: Experience of a central London hospital. Clin Rheumatol [Internet]. 2011;30(3):399–401. Available from: https://www.embase.com/search/results?subaction=viewrecord&id=L51123035&from=export

241.	Mañosa M, Domènech E, Cabré E. Current incidence of active tuberculosis in IBD patients treated with anti-TNF agents: Still room for improvement. J Crohn’s Colitis. 2013 Nov;7(10):e499–500.

242.	Marano C, Hsia E, Xu S, Sandborn W, Rutgeerts P. Interferon-Y Release Assay versusTuberculin Skin Test in Patients with Moderate-to-Severely Active Ulcerative Colitis: Results from the PURSUIT UC Program. Can J Gastroenterol Hepatol. 2016;(Program and Abstracts from the Canadian Digestive Diseases Week 2016):A273.

243.	Mariano V, Andrade L, Oliveira F, Santos M, Pereira F, Dos Santos C, et al. Tuberculosis in inflammatory bowel disease patients from a referral center in Salvador-Bahia, Brazil. Am J Gastroenterol [Internet]. 2019;114((Mariano V.; Oliveira F.; Santos M.; Pereira F.; Dos Santos C.) University of the State of Bahia (UNEB), Salvador, Bahia, Brazil(Andrade L.) Faculty of Technology and Sciences (FTC), Salvador, Brazil(Almeida N.; Surlo V.; Fortes F.M.; Pimentel A.; Fontes):S20–1. Available from: https://www.embase.com/search/results?subaction=viewrecord&id=L629361904&from=export

244.	Mariette X, Baron G, Liote F, Goupille P, Flipo R-M, Combe B, et al. Before beginning anti-TNF, a better targeted screening and a twice decrease frequency of latent tuberculosis (TB) with IFN gamma release assays (IGRA) compared with tuberculin skin test. Results in 396 patients from the etat study. Arthritis Rheum [Internet]. 2010;62:376. Available from: https://www.embase.com/search/results?subaction=viewrecord&id=L70380403&from=export

245.	Mariette X, Vencovsky J, Lortholary O, Gomez-Reino J, M de L, Ralston P, et al. The incidence of tuberculosis in patients treated with certolizumab pegol across indications: impact of baseline skin test results, more stringent screening criteria and geographic region. RMD Open. 2015;1(1):e000044-.

246.	Martin J, Walsh C, Gibbs A, McDonnell T, Fearon U, Keane J, et al. Comparison of interferon γ release assays and conventional screening tests before tumour necrosis factor α blockade in patients with inflammatory arthritis. Ann Rheum Dis. 2010 Jan;69(01):181–5.

247.	Mateo L, Minguez S, Latorre I, Dominguez J, Molto A, Garcia E, et al. Usefulness of in vitro interferon-release assays (IGRAS) for diagnosis of latent tuberculosis infection in rheumatic patients scheduled for anti-TNF-treatment. Arthritis Rheum [Internet]. 2009;60((Mateo L.; Latorre I.; Dominguez J.; Grados D.) Hospital Germans Trias i Pujol, Badalona, Spain(Minguez S.; Garcia E.) Hospital Universitari Germans Trias i Pujol, Badalona, Spain(Molto A.) M.D., Resident, Badalona, Spain):996. Available from: https://www.embase.com/search/results?subaction=viewrecord&id=L70374862&from=export

248.	Matsumura R, Igari H, Nakazawa T, Ishikawa S, Tsuyuzaki M, Suzuki K, et al. Comparative utility of interferon-γ release assay, QuantiFERON&lt;SUP&gt;®&lt;/SUP&gt; TB-GIT and T-SPOT&lt;SUP&gt;®&lt;/SUP&gt;.&lt;I&gt;TB&lt;/I&gt; in rheumatoid arthritis. Int J Tuberc Lung Dis. 2016 Nov 1;20(11):1546–53.

249.	McInnes IB, Mease PJ, Reich K, Nash P, Widmer A, Abrams K, et al. Secukinumab demonstrates a consistent safety profile with up to 5 years’ treatment in patients with psoriatic arthritis and moderate-tosevere plaque psoriasis: Updated pooled safety analyses. Rheumatol (United Kingdom) [Internet]. 2019;58((McInnes I.B.) Institute of Infection,Immunity and Inflammation, University of Glasgow, Glasgow, United Kingdom(Mease P.J.) Rheumatology Department, Swedish Medical Center and University of Washington, Seattle, WA, United States(Reich K.) Dermatology, Der):iii150-. Available from: https://www.embase.com/search/results?subaction=viewrecord&id=L628382427&from=export

250.	Mckay A, Kraut A, Murdzak C, Yassi A. Determinants of Tuberculin Reactivity among Health Care Workers: Interpretation of Positivity following BCG Vaccination. Can J Infect Dis. 1999;10(2):134–9.

251.	Mease PJ, McInnes IB, Reich K, Nash P, Andersson M, Abrams K, et al. Secukinumab demonstrates consistent safety over long-term exposure in patients with psoriatic arthritis and moderate to severe plaque psoriasis: Updated pooled safety analyses. Arthritis Rheumatol [Internet]. 2017;69((Mease P.J.) Swedish Medical Center, University of Washington, Seattle, WA, United States(McInnes I.B.) University of Glasgow, Glasgow, United Kingdom(Reich K.) Dermatologikum Hamburg, Hamburg, Germany(Nash P.) University of Queensland, Brisbane, Australi). Available from: https://www.embase.com/search/results?subaction=viewrecord&id=L618910822&from=export

252.	Megna M, Ferrillo M, Ruggiero A, Cinelli E, Gallo L, Fabbrocini G. QuantiFERON TB-gold conversion rate among psoriasis patients under biologics: a 9-year retrospective study. Int J Dermatol [Internet]. 2021;60(3):352–7. Available from: https://www.embase.com/search/results?subaction=viewrecord&id=L2006808675&from=export

253.	Mehta B, Zapantis E, Petryna O, Efthimiou P. Screening Optimization of Latent Tuberculosis Infection in Rheumatoid Arthritis Patients. Arthritis. 2015 Jul 29;2015:1–8.

254.	Mehta B, Sohn B, Efthimiou P V. Combining tuberculin skin test and interferon gamma release assays for latent tuberculosis infection screening may be necessary for the exclusion of latent tuberculosis in a high risk individuals with rheumatoid arthritis. Arthritis Rheum [Internet]. 2011;63(10). Available from: https://www.embase.com/search/results?subaction=viewrecord&id=L70785124&from=export

255.	Melath S, Ismajli M, Smith R, Patel I, Steuer A. Screening for latent TB in patients with rheumatic disorders prior to biologic agents in a ‘high-risk’ TB population: comparison of two interferon gamma release assays. Rheumatol Int. 2014 Jan 28;34(1):149–50.

256.	Ministro P, Machado J, Araújo R, Castanheira A, Cancela EM, Silva A. Diagnosis of Latent Tuberculosis in Patients With Inflammatory Bowel Disease: Prospective Comparison Between Tuberculin Skin Test and Interferon Gamma Release Assay (IGRA) Test. Gastroenterology. 2011 May;140(5):S-776.

257.	Miossi R, Bonfiglioli KR, Saad CGS, Ribeiro AC, Moraes JCB, Bonfá E. Latent tuberculosis screening and treatment in ankylosing spondylitis patients eligible for anti-TNF therapy in endemic area. Arthritis Rheum [Internet]. 2013;65((Miossi R.; Bonfiglioli K.R.; Saad C.G.S.; Ribeiro A.C.; Moraes J.C.B.; Bonfá E.) Faculdade de Medicina, Universidade de São Paulo, São Paulo, Brazil):S645-. Available from: https://www.embase.com/search/results?subaction=viewrecord&id=L71318990&from=export

258.	Mittal C, Kaur N. QuantiFERON TB gold testing for latent tuberculosis is more frequently indeterminate in patients with inflammatory bowel disease. Inflamm Bowel Dis [Internet]. 2013;19((Mittal C.; Kaur N.) Henry Ford Health System, Detroit, MI, United States):S62-. Available from: https://www.embase.com/search/results?subaction=viewrecord&id=L71355901&from=export

259.	Mohan AK, Coté TR, Block JA, Manadan AM, Siegel JN, Braun MM. Tuberculosis following the use of etanercept, a tumor necrosis factor inhibitor. Clin Infect Dis [Internet]. 2004;39(3):295–9. Available from: https://www.embase.com/search/results?subaction=viewrecord&id=L39050474&from=export

260.	Molnár T, Farkas K, Nagy F, Szepes Z, Bálint A, Bor R, et al. Frequency and outcome of tuberculosis in patients with inflammatory bowel disease receiving infliximab in our tertiary center. United Eur Gastroenterol J [Internet]. 2013;1(1):A516-. Available from: https://www.embase.com/search/results?subaction=viewrecord&id=L72264603&from=export

261.	Mulero P, Caminero AB, Neri Crespo MJ, Fernández-Herranz R, Téllez Lara N. Latent tuberculosis seems not to reactivate in multiple sclerosis patients on natalizumab. J Neuroimmunol. 2012 Feb;243(1–2):103–5.

262.	Munoz L, Casas S, Juanola X, Bordas X, Martinez C, Santin M. Prevention of Anti-Tumor Necrosis Factor-Associated Tuberculosis: A 10-Year Longitudinal Cohort Study. Clin Infect Dis. 2015 Feb 1;60(3):349–56.

263.	Mustapha MG, Garba AM, Rabasa AI, Gimba MS. Prevalence of Mantoux test positivity among apparently healthy children in Maiduguri, Nigeria. SAJCH South African J Child Heal [Internet]. 2009;3(3):80–2. Available from: https://www.embase.com/search/results?subaction=viewrecord&id=L358053457&from=export

264.	Mutlu P, Sevinç C, Kilinç O, Sabri Uçan E. Follow up results of latent tuberculosis infection in patients treated with anti-TNF-alpha. Nobel Med [Internet]. 2014;10(1):47–52. Available from: https://www.embase.com/search/results?subaction=viewrecord&id=L372862260&from=export

265.	Nasr I, Goel R, Ward M, Fong S, Patel K, Sastrillo M, et al. Indeterminate and inconclusive results are common when using interferon gamma release assay as screening for TB in patients with IBD. Gut [Internet]. 2014;63((Nasr I.; Goel R.; Ward M.; Fong S.; Patel K.; Sastrillo M.; Anderson S.; Sanderson J.; Irving P.) Gastroenterology, Guy’s and St Thomas’ NHS Foundation Trust, London, United Kingdom):A77-. Available from: https://www.embase.com/search/results?subaction=viewrecord&id=L71542237&from=export

266.	Nisar MK, Rafiq A, Östör AJK. Biologic therapy for inflammatory arthritis and latent tuberculosis: real world experience from a high prevalence area in the United Kingdom. Clin Rheumatol [Internet]. 2015;34(12):2141–5. Available from: https://www.embase.com/search/results?subaction=viewrecord&id=L606613509&from=export

267.	Nobre CA, Callado MRM, Lima JRC, Gomes KWP. Tuberculosis infection in rheumatic patients with infliximab therapy: Experience with 157 patients. Rheumatol Int [Internet]. 2012;32(9):2769–75. Available from: https://www.embase.com/search/results?subaction=viewrecord&id=L365658636&from=export

268.	Nusslein H, Alten R, Galeazzi M, Lorenz HM, Boumpas D, Nurmohamed MT, et al. Real-world efficacy and safety of abatacept treatment for rheumatoid arthritis: 12-month interim analysis of the action study. Rheumatol (United Kingdom) [Internet]. 2013;52:i90–1. Available from: https://www.embase.com/search/results?subaction=viewrecord&id=L71070895&from=export

269.	Ocal S, Demir N, Ataman S, Demir AU, Suldur N, Kaya A, et al. Differences in the manegement of latent tuberculosis infection in patients treated with anti-TNFα in Turkey. Eur Respir J [Internet]. 2012;40((Ocal S.; Demir N.; Ataman S.; Demir A.U.; Suldur N.; Kaya A.; Kayacan O.)). Available from: https://www.embase.com/search/results?subaction=viewrecord&id=L71926216&from=export

270.	Opris D, Mazilu D, Saulescu I, Ionescu R. Is tuberculosis screening sufficient for preventing TB reactivation in biologic treated patients? Ann Rheum Dis [Internet]. 2014;73((Opris D.; Mazilu D.; Saulescu I.; Ionescu R.) Rheumatology, Sfanta Maria Hospital, Bucharest, Romania). Available from: https://www.embase.com/search/results?subaction=viewrecord&id=L71552074&from=export

271.	Ortaköylü M, Bahadir A, Soy D, Caglar E, Paker N, Alkan F, et al. Concordance of the Interferon-γ Release Assay (IGRA) and the Tuberculin Skin Test (TST) for the Screening of Tuberculosis Infection in the Inflammatory Rheumatic Disease (IRD) Population. Chest. 2012 Oct;142(4):211A.

272.	Owen L, Thi AA, Bouri S, Wolfson P, Abbara A, John L, et al. Active tuberculosis in patients with inflammatory bowel disease after treatment with anti-TNF therapy. Gut [Internet]. 2016;65((Owen L.; Thi A.A.; Bouri S.; Wolfson P.; Hart A.L.) Department of Inflammatory Bowel Disease, St Mark’s Hospital, London, United Kingdom(Abbara A.; John L.; Davidson R.N.) Department of Infectious Diseases, Northwick Park Hospital, London, United Kingdom):A252–3. Available from: https://www.embase.com/search/results?subaction=viewrecord&id=L613507142&from=export

273.	Özlü C, Türkuçar S, Asrak HK, Dündar HA, Ünsal ŞE, Belet N. Tuberculosis risk in the biologic era: tuberculin skin test conversion rates in children with rheumatologic diseases. Turk J Pediatr. 2021 Dec 25;63(6):978–85.

274.	Paluch-Oleś J, Magryś A, Kozioł-Montewka M, Koszarny A, Majdan M. Identification of latent tuberculosis infection in rheumatic patients under consideration for treatment with anti-TNF-α agents. Arch Med Sci. 2013;1:112–7.

275.	Papay P, Primas C, Eser A, Novacek G, Winkler S, Frantal S, et al. Retesting for latent tuberculosis in patients with inflammatory bowel disease treated with TNF‐α inhibitors. Aliment Pharmacol Ther. 2012 Nov 15;36(9):858–65.

276.	Papay P, Eser A, Winkler S, Frantal S, Primas C, Miehsler W, et al. Predictors of indeterminate IFN-γ release assay in screening for latent TB in inflammatory bowel diseases. Eur J Clin Invest. 2011 Oct;41(10):1071–6.

277.	Papay P, Eser A, Miehsler W, Lichtenberger C, Mikulits A, Dejaco C, et al. Immunosuppressive (IS) therapy impacts the results of QuantiFERON® and tuberculin skin test in routine screening for latent tuberculosis (LTB) in patients with inflammatory bowel diseases (IBD). Gastroenterology [Internet]. 2009;136(5):A195-. Available from: https://www.embase.com/search/results?subaction=viewrecord&id=L70151457&from=export

278.	Papp K, Gooderham M, Morita A, Kivitz A, Sinvhal R, Topp A, et al. Safety and efficacy results from the open label extension of a phase 2 trial of risankizumab, a selective IL-23p19 inhibitor in patients with active psoriatic arthritis. Arthritis Rheumatol [Internet]. 2019;71((Papp K.) K. Papp Clinical Research and Probity Medical Research, Waterloo, ON, Canada(Gooderham M.) School of Medicine, Queen ’ s University, Kingston, ON and Skin Centre for Dermatology, Probity Medical Research, Peterborough, ON, Canada(Morita A.) Dept):5111–3. Available from: https://www.embase.com/search/results?subaction=viewrecord&id=L633057937&from=export

279.	PARK JH, SEO GY, LEE JS, KIM T-H, YOO D-H. Positive Conversion of Tuberculin Skin Test and Performance of Interferon Release Assay to Detect Hidden Tuberculosis Infection During Anti-Tumor Necrosis Factor Agent Trial. J Rheumatol. 2009 Oct;36(10):2158–63.

280.	Park JW, Curtis JR, Lee H, Lee J-K, Song YW, Lee EB. Risk-benefit analysis of isoniazid monotherapy to prevent tuberculosis in patients with rheumatic diseases exposed to prolonged, high-dose glucocorticoids. PLoS One. 2020 Dec 31;15(12):e0244239.

281.	Patricia M, Baraniuk AO, Aikawa NE, Bombarda S, Seiscento M, Silva CAA, et al. Tuberculin skin test response in patients with juvenile idiopathic arthritis. Ann Rheum Dis [Internet]. 2015;74((Patricia M.; Baraniuk A.O.) Ponificia Universidade Católica Do Paraná, Curitiba, Brazil(Patricia M.) Hospital Universitário Evangélico De Curitiba, Curitiba, Brazil(Aikawa N.E.; Silva C.A.A.; Goldenstein-Schainberg C.) Rheumathology, São Paulo, Brazil(Bo):841. Available from: https://www.embase.com/search/results?subaction=viewrecord&id=L72153276&from=export

282.	Pattanaik SS, Muhammed H, Aggarwal A, Lawrence A, Agarwal V, Misra DP, et al. Causes of death in SLE: Analysis of inpatient death from 2000-2018 in a tertiary care Hospital in India. Arthritis Rheumatol [Internet]. 2019;71((Pattanaik S.S.) Sanjay Gandhi Post Graduate Institute of Medical Sciences, Lucknow, Uttar Pradesh, India(Muhammed H.; Agarwal V.; Misra D.P.) SGPGI, Lucknow, Uttar Pradesh, India(Aggarwal A.) Sanjay Gandhi Postgraduate Institute of Medical Sciences, Luck):2805–6. Available from: https://www.embase.com/search/results?subaction=viewrecord&id=L633058073&from=export

283.	Piñeiro R, Mellado MJ, Cilleruelo MJ, García-Ascaso M, Medina-Claros A, García-Hortelano M. Tuberculin skin test in bacille Calmette–Guérin-vaccinated children: how should we interpret the results? Eur J Pediatr. 2012 Nov 1;171(11):1625–32.

284.	Piovezani Ramos G, Stroh G, Al Bawardy B, Faubion WA, Papadakis KA, Escalante P. Outcomes of treatment for latent tuberculosis infection in patients with inflammatory bowel disease receiving biologic therapy. United Eur Gastroenterol J [Internet]. 2017;5(5):A753–4. Available from: https://www.embase.com/search/results?subaction=viewrecord&id=L619890113&from=export

285.	Ponce de Leon D. Attenuated response to purified protein derivative in patients with rheumatoid arthritis: study in a population with a high prevalence of tuberculosis. Ann Rheum Dis. 2005 Sep 1;64(9):1360–1.

286.	Prignano F, Bartoloni A, Bartalesi F, Gori A, Ricceri F, Cavallo A, et al. Latent tuberculosis infection in psoriasis and other dermatological immunomediated diseases: a combined approach by <scp>Q</scp> uanti <scp>FERON</scp> ^®^ ‐ <scp>TB G</scp> old and tuberculin skin tests. Int J Dermatol. 2014 Aug 5;53(8).

287.	Puig L, Tsai T-F, Bhutani T, Uy J, Ramachandran P, Song M, et al. Safety in moderate-to-severe plaque psoriasis patients with latent tuberculosis treated with guselkumab and anti-tuberculosis treatments concomitantly: results from pooled phase 3 VOYAGE 1 & VOYAGE 2 trials. J Eur Acad Dermatology Venereol [Internet]. 2020;34(8):1744–9. Available from: https://www.embase.com/search/results?subaction=viewrecord&id=L2004926030&from=export

288.	Puri AS, Desai D, Sood A, Sachdeva S. Infliximab-induced tuberculosis in patients with UC: Experience from India—a country with high prevalence of tuberculosis. J Gastroenterol Hepatol [Internet]. 2017;32(6):1191–4. Available from: https://www.embase.com/search/results?subaction=viewrecord&id=L616607250&from=export

289.	Ramos GP, Stroh G, Al-Bawardy B, Faubion WA, Papadakis KA, Escalante P. Outcomes of treatment for latent tuberculosis infection in patients with inflammatory bowel disease receiving biologic therapy. Inflamm Bowel Dis [Internet]. 2018;24(10):2272–7. Available from: https://www.embase.com/search/results?subaction=viewrecord&id=L625011579&from=export

290.	Ramos L, Hernandez Camba A, Carrillo Palau M, Alonso I, Hernandez Alvarez-Buylla N, Quintero Carrion E. Outcome of treatment with biological agents in Crohn’s disease: 117 patients in 5 years from a tertiary referral center. J Crohn’s Colitis [Internet]. 2014;8((Ramos L.; Hernandez Camba A.; Carrillo Palau M.; Alonso I.; Hernandez Alvarez-Buylla N.; Quintero Carrion E.) Hospital Universitario de Canarias, Gastroenterology, La Laguna, SantaCruz de Tenerife, Spain):S231-. Available from: https://www.embase.com/search/results?subaction=viewrecord&id=L71389061&from=export

291.	Ramos S, Nogueira A, Dias A, Gonçalves AF, Gaio AR, Duarte R. Tuberculosis screening in patients receiving biological therapy. Acta Reum Port. 2015;40(3):234–40.

292.	Raza SH, Islam S, Freire A, Pattanaik D, Stuart J. Conversion rate of tuberculosis screening tests in patients with rheumatic diseases while receiving anti-tumor necrosis factor alpha (anti-TNFα) agents. Arthritis Rheumatol [Internet]. 2016;68((Raza S.H.) Rheumatology, University of Tennessee, Health Science Center, Memphis, TN, United States(Islam S.) University of Tennessee, Health Science Center, Memphis, TN, United States(Freire A.) Pulmonology-Critical Care, University of Tennessee, Heal):3510–2. Available from: https://www.embase.com/search/results?subaction=viewrecord&id=L613888472&from=export

293.	Ritchlin C, Helliwell P, Boehncke W-H, Hsia E, Kollmeier A, Subramanian R, et al. Guselkumab, an IL-23 Inhibitor That Specifically Binds to the IL23p19-Subunit, for Active Psoriatic Arthritis: One Year Results of a Phase 3, Randomized, Double-Blind, Placebo-Controlled Study of Patients Who Were Biologic-Naïve or TNFα Inhibitor-Experien. Arthritis Rheumatol [Internet]. 2020;72(SUPPL 10):2705–9. Available from: https://www.embase.com/search/results?subaction=viewrecord&id=L634233101&from=export

294.	Romiti R, Valenzuela F, Chouela EN, Xu W, Pangallo B, Moriarty SR, et al. Prevalence and outcome of latent tuberculosis in patients receiving ixekizumab: integrated safety analysis from 11 clinical trials of patients with plaque psoriasis. Br J Dermatol [Internet]. 2019;181(1):202–3. Available from: https://www.embase.com/search/results?subaction=viewrecord&id=L626736386&from=export

295.	Rotar Z, Svetina P, Tomsic M, Hočevar A, Prapotnik S. Tuberculosis among patients treated with TNF inhibitors for rheumatoid arthritis, ankylosing spondylitis and psoriatic arthritis in Slovenia: A cohort study. BMJ Open [Internet]. 2020;10(2). Available from: https://www.embase.com/search/results?subaction=viewrecord&id=L630825912&from=export

296.	Rotar Ž, Tomsic M. Incidence rate of tuberculosis in rheumatoid arthritis patients treated with TNF-α inhibitors-data from the slovenian national BioRx.Si registry. Ann Rheum Dis [Internet]. 2016;75((Rotar Ž.; Tomsic M.) Department of Rheumatology, University Medical Centre Ljubljana, Ljubljana, Slovenia):1016–7. Available from: https://www.embase.com/search/results?subaction=viewrecord&id=L612777848&from=export

297.	Rousset S, Treiner E, Moulis G, Pugnet G, Astudillo L, Paricaud K, et al. High rate of indeterminate results of the QuantiFERON-TB Gold in-tube test, third generation, in patients with systemic vasculitis. Rheumatol (United Kingdom) [Internet]. 2020;59(5):1006–10. Available from: https://www.embase.com/search/results?subaction=viewrecord&id=L632124143&from=export

298.	Ruksenaite J, Ward T, Tsaknis G, Hussain S-F. Prevention of latent TB reactivation in rheumatology patients prior to anti-TNF therapy: Evaluation of a risk stratification strategy in a low prevalence area. Eur Respir J [Internet]. 2015;46((Ruksenaite J.; Ward T.; Tsaknis G.; Hussain S.-F.)). Available from: https://www.embase.com/search/results?subaction=viewrecord&id=L72108624&from=export

299.	Sahin S, Bektas S, Belhan E, Hopurcuoglu D, Adrovic A, Barut K, et al. Evaluation of long term safety and indications of biologic agents in juvenile rheumatic diseases: A single center experience in Turkey. Pediatr Rheumatol [Internet]. 2017;15((Sahin S.; Bektas S.; Belhan E.; Hopurcuoglu D.; Adrovic A.; Barut K.; Kasapcopur O.) Department of Pediatric Rheumatology, Istanbul University, Cerrahpasa Medical School, Istanbul, Turkey):168. Available from: https://www.embase.com/search/results?subaction=viewrecord&id=L618421501&from=export

300.	Sakiyama M, Kozaki Y, Komatsu T, Niwa K, Suzuki H, Ota M, et al. Specificity of tuberculin skin test improved by BCG immunization schedule change in Japan. J Infect Chemother. 2021 Sep;27(9):1306–10.

301.	Salinas MJH, Benzaquén N, Pirola JP, Alvarellos A, Caeiro F, Álvarez C, et al. Tuberculosis in patients treatedwith biological drugs. J Clin Rheumatol [Internet]. 2016;22(3):159–60. Available from: https://www.embase.com/search/results?subaction=viewrecord&id=L614627707&from=export

302.	Sanchez Riera L, Wilson N, Al Izzi M, Hussein I, Nuhaily S, Qahtani N, et al. Quantiferon-tb more useful than tuberculin skin test for latent tuberculosis screening: A hospital experience. Ann Rheum Dis [Internet]. 2014;73((Sanchez Riera L.; Wilson N.; Hussein I.; Nuhaily S.; Qahtani N.; Ibrahim N.; Aneja R.; Khan T.; Gonuguntla S.; Maashari H.; Waheeduddin S.; Al Maini M.) Department of Rheumatology, Mafraq Hospital, Abu Dhabi, United Arab Emirates(Al Izzi M.) Mediclinic W). Available from: https://www.embase.com/search/results?subaction=viewrecord&id=L71553207&from=export

303.	Sánchez‐Moya A, Dauden E. Incidence of tuberculosis infection in psoriatic patients on anti‐TNF therapy: report of a case series with 144 patients. J Eur Acad Dermatology Venereol. 2011 Jun 14;25(6):730–3.

304.	Saoula H, Boutaleb A, Bouchelghoum T, Ait Hamadouche N, Zmiri Y, Chemanedji F, et al. Tuberculosis infection in IBD patients on antitnf therapy: An Algerian retrospective study. J Crohn’s Colitis [Internet]. 2015;9((Saoula H.; Boutaleb A.; Bouchelghoum T.; Ait Hamadouche N.; Zmiri Y.; Chemanedji F.; Aissaoui M.; Osmane R.; Hamidouche D.; Salah A.; Malaoui L.; Mahiou H.; Nakmouche M.) CHU of Bab El Oued, Gastroenterology, Algiers, Algeria(Gamar L.; Kecili L.; Bounab):S379–80. Available from: https://www.embase.com/search/results?subaction=viewrecord&id=L71994973&from=export

305.	Sargın G, Şentürk T, Ceylan E, Telli M, Çildağ S, Doğan H. Tst, Quantiferon-tb Gold test and t-spot.Tb test for detecting latent tuberculosis infection in patients with rheumatic disease prior to anti-tnf therapy. Tuberk Toraks. 2018 Jun 30;66(2):136–43.

306.	Sartori NS, Picon P, Papke A, Neyeloff JL, da Silva Chakr RM. A population-based study of tuberculosis incidence among rheumatic disease patients under anti-TNF treatment. PLoS One. 2019 Dec 2;14(12):e0224963.

307.	Sauzullo I, Mengoni F, Scrivo R, Valesini G, Potenza C, Skroza N, et al. Usefulness of interferon-gamma release assays for latent tuberculosis screening in patients candidate for TNF-a therapy. Clin Microbiol Infect. 2010;16(Suppl 2):O332.

308.	Sayyahfar S, Karimi A, Fahimzad A, Shamshiri AR. Comparison of Tuberculin Skin Test result and interferon gamma response to human PPD in BCG scar positive and negative children. J Epidemiol Glob Health. 2013;4(1):45.

309.	Schneeberger EE, Salas A, Medina LF, Zacariaz JB, Mantilla RD, Sarmiento-Monroy JC, et al. Real-world use of tofacitinib in rheumatoid arthritis: Data from Latin America. J Clin Rheumatol [Internet]. 2018;24(3):S73–4. Available from: https://www.embase.com/search/results?subaction=viewrecord&id=L622213742&from=export

310.	Schwartzman S, Sandoval D, Kronbergs A, Lisse J, Patel H, Xu W, et al. Long-Term Safety Profile of Ixekizumab Treatment in Patients with Axial Spondyloarthritis. Arthritis Rheumatol [Internet]. 2020;72(SUPPL 10):1778–80. Available from: https://www.embase.com/search/results?subaction=viewrecord&id=L634231775&from=export

311.	Seong S-S, Choi C-B, Woo J-H, Kang WB, Joung C-L, Uhm W-S, et al. Incidence of tuberculosis in Korean patients with Rheumatoid Arthritis (RA): Effects of RA itself and of tumor necrosis factor blockers. J Rheumatol [Internet]. 2007;34(4):706–11. Available from: https://www.embase.com/search/results?subaction=viewrecord&id=L46641374&from=export

312.	Shah I, Kathwate J, Shetty N. Comparison of tuberculin skin test and QuantiFERON-TB Gold In-Tube test in Bacillus Calmette–Guerin-vaccinated children. Lung India. 2020;37(1):24.

313.	Shaikh S, Bensen W, Chow A, Dixit S, Fortin I, Haraoui B, et al. Safety and effectiveness of TNF-alpha inhibitor therapy with certolizumab pegol observed in daily practice in adult rheumatoid arthritis patients in Canada-first interim analysis of the noninterventional fast can study. J Rheumatol [Internet]. 2013;40(6):961. Available from: https://www.embase.com/search/results?subaction=viewrecord&id=L71621540&from=export

314.	Shen Y, Ma H-F, Luo D, Guan J-L. The T-SPOT.TB assay used for screening and monitoring of latent tuberculosis infection in patients with Behçet’s disease pre- and post-anti-TNF treatment: A retrospective study. J Chinese Med Assoc [Internet]. 2019;82(5):375–80. Available from: https://www.embase.com/search/results?subaction=viewrecord&id=L627747190&from=export

315.	Shimabuco AY, Medeiros-Ribeiro AC de, Miossi R, Bonfiglioli KR, Moraes JCB de, Gonçalves CR, et al. Ankylosing spondylitis and psoriatic arthritis: revisiting screening of latent tuberculosis infection and its follow-up during anti-tumor necrosis factor therapy in an endemic area. Clinics [Internet]. 2020;75:e1870–e1870. Available from: http://www.scielo.br/scielo.php?script=sci_arttext&pid=S1807-59322020000100292

316.	Shin A, Lee YJ, Lee EB, Song YW, Kim SC, Kang EH. Tuberculosis risk with biologics by screening-guided preventive strategy in rheumatoid arthritis under intermediate tuberculosis burden. Rheumatology. 2021 Jun 18;60(6):2755–64.

317.	Shobha V, Chandrashekara S, Rao V, Desai A, Jois R, Dharmanand BG, et al. Biologics and risk of tuberculosis in autoimmune rheumatic diseases: A real-world clinical experience from India. Int J Rheum Dis [Internet]. 2019;22(2):280–7. Available from: https://www.embase.com/search/results?subaction=viewrecord&id=L623777183&from=export

318.	Shobha V, Rao V, Desai A, Jois R, Srikantiah C, Dharmanand BG, et al. Tuberculosis profile in biologics experienced patients-Karnataka Biologics Cohort (KBC) study group experience. Indian J Rheumatol [Internet]. 2016;11(5):S43–4. Available from: https://www.embase.com/search/results?subaction=viewrecord&id=L619195068&from=export

319.	Shu D, Zhang Z, Zhou EY, Ma X, Zhao Y. Is chemoprophylaxis necessary for all latent tuberculosis infection patients receiving IL-17 inhibitors? A cohort study. Dermatol Ther [Internet]. 2020;33(6). Available from: https://www.embase.com/search/results?subaction=viewrecord&id=L2007411076&from=export

320.	Sichletidis L, Settas L, Spyratos D, Chloros D, Patakas D. Tuberculosis in patients receiving anti-TNF agents despite chemoprophylaxis. Int J Tuberc Lung Dis [Internet]. 2006;10(10):1127–32. Available from: https://www.embase.com/search/results?subaction=viewrecord&id=L44519351&from=export

321.	Siffledeen JS, Wotton C, Travis S, Goldacre M. High incidence of TB in large hospital database-based studies of IBD patients in the ERA of biologics. Gastroenterology [Internet]. 2012;142(5):S671. Available from: https://www.embase.com/search/results?subaction=viewrecord&id=L71107808&from=export

322.	Simon TA, Boers M, Hochberg M, Baker N, Skovron ML, Ray N, et al. Comparative risk of malignancies and infections in patients with rheumatoid arthritis initiating abatacept versus other biologics: a multi-database real-world study. Arthritis Res Ther. 2019 Dec 8;21(1):228.

323.	Simon TA, Dong L, Winthrop KL. Risk of opportunistic infections in patients with rheumatoid arthritis initiating abatacept: cumulative clinical trial data. Arthritis Res Ther. 2021 Dec 11;23(1):17.

324.	Sleiman R, Al-Tannir M, Dakdouki G, Ziade F, Assi NA, Rajab M. Interpretation of the Tuberculin Skin Test in Bacille Calmette-Gu??rin Vaccinated and Nonvaccinated School Children. Pediatr Infect Dis J. 2007 Feb;26(2):134–8.

325.	Slogotskaya L, Bogorodskaya E, Ivanova D, Sevostyanova T. Comparative sensitivity of the test with tuberculosis recombinant allergen, containing ESAT6-CFP10 protein, and Mantoux test with 2 TU PPD-L in newly diagnosed tuberculosis children and adolescents in Moscow. PLoS One. 2018 Dec 21;13(12):e0208705.

326.	Slouma M, Mahmoud I, Saidane O, Bouden S, Abdelmoula L. Latent tuberculosis infection screening prior to biological treatment in Tunisian patients. Therapie [Internet]. 2017;72(5):573–8. Available from: https://www.embase.com/search/results?subaction=viewrecord&id=L614877425&from=export

327.	So H, Yuen CS, Yip RM. Comparison of a commercial interferon-gamma release assay and tuberculin skin test for the detection of latent tuberculosis infection in Hong Kong arthritis patients who are candidates for biologic agents. Hong Kong Med J. 2017 Jan 27;

328.	Soare A, Gheorghiu AM, Aramă V, Bumbăcea D, Dobrotă R, Oneaţă R, et al. Risk of active tuberculosis in patients with inflammatory arthritis receiving TNF inhibitors: a look beyond the baseline tuberculosis screening protocol. Clin Rheumatol. 2018 Sep 17;37(9):2391–7.

329.	Socaci A, Nini G, Borgazi E, Tudorache V. Comparison between tuberculin skin testing and QuantiFERON Gold-In Tube for detection of latent tuberculosis in rheumatoid arthritis patients in a TB endemic population. Eur Respir J [Internet]. 2011;38. Available from: https://www.embase.com/search/results?subaction=viewrecord&id=L72122552&from=export

330.	Solovan C, Timofte A, Chiticariu E, Boghian L, Stoia C. Tuberculin skin test as a tool for detecting latent tuberculosis in patients with psoriasis in a highly endemic setting. Br J Dermatol [Internet]. 2011;165(6):e17–8. Available from: https://www.embase.com/search/results?subaction=viewrecord&id=L70610787&from=export

331.	Son CN, Kim TH, Jun JB, Sung IH, Yoo DH. Repeated interferon gamma release assays in Ankylosing Spondylitis. Int J Rheum Dis [Internet]. 2014;17((Son C.N.; Kim T.H.; Jun J.B.; Yoo D.H.) Rheumatology, Hanyang University Hospital for Rheumatic Diseases, Seoul, South Korea(Sung I.H.) Orthopaedic Surgery, Hanyang University Hospital for Rheumatic Diseases, Seoul, South Korea):78. Available from: https://www.embase.com/search/results?subaction=viewrecord&id=L71477346&from=export

332.	SONG Y-J, Soo-Kyung CHO, Hyoungyoung KIM, Hye-Won KIM, Eunwoo NAM, Sang-Cheol BAE, et al. Risk of Tuberculosis Development in Patients with Rheumatoid Arthritis Receiving Targeted Therapy:a Prospective Single Center Cohort Study. J Korean Med Sci [Internet]. 2021;e70–e70. Available from: http://dx.doi.org/10.3346/jkms.2021.36.e70

333.	Soriano ER, Elewski B, Baddley J, Deodhar A, Magrey M, Rich P, et al. Lack of reactivation of tuberculosis in 12,319 patients with psoriasis, psoriatic arthritis and ankylosing spondylitis treated with secukinumab: An ad hoc analysis of pooled safety data from 28 clinicaltrials. J Clin Rheumatol [Internet]. 2020;20(3 SUPPL 1):S65–6. Available from: https://www.embase.com/search/results?subaction=viewrecord&id=L633472080&from=export

334.	Sousa M, Ladeira I, Fernandes C, Ponte A, Rodrigues A, Silva AP, et al. Latent and active tuberculosis in patients with inflammatory bowel disease under anti-TNF-data from a centre with high incidence of tuberculosis. J Crohn’s Colitis [Internet]. 2019;13((Sousa M.; Ladeira I.; Fernandes C.; Ponte A.; Rodrigues A.; Silva A.P.; Rodrigues J.; Silva J.; Gomes C.; Carvalho J.) Centro Hospitalar De Vila Nova De Gaia E Espinho, Vila Nova De Gaia, Portugal):S150-. Available from: https://www.embase.com/search/results?subaction=viewrecord&id=L628410901&from=export

335.	Stone CD, Chen J, Armbrecht ES. Utility of annual testing for tuberculosis infection during treatment with anti-tumor necrosis factor alpha medications. Gastroenterology [Internet]. 2012;142(5):S206-. Available from: https://www.embase.com/search/results?subaction=viewrecord&id=L71105997&from=export

336.	Subramani R, Datta M, Swaminathan S. Does effect of BCG vaccine decrease with time since vaccination and increase tuberculin skin test reaction? Indian J Tuberc. 2015 Oct;62(4):226–9.

337.	Sundbaum JK, Arkema E V, Bruchfeld J, Jonsson J, Askling J, Baecklund E. Tuberculosis in biologic-naïve patients with rheumatoid arthritis: Risk factors and tuberculosis characteristics. J Rheumatol [Internet]. 2021;48(8):1243–50. Available from: https://www.embase.com/search/results?subaction=viewrecord&id=L2013980494&from=export

338.	Sürücüoğlu S, Türel Ermertcan A, Çetinarslan T, Özkütük N. The reliability of tuberculin skin test in the diagnosis of latent tuberculosis infection in psoriasis patients: A case‐control study. Dermatol Ther. 2020 Jul 18;33(4).

339.	Sztajnbok F, Boechat N, Oliveira S, Ribeiro S, Rodrigues M, Diniz C, et al. Latent tuberculosis infection in patients with juvenile idiopathic arthritis undergoing methotrexate therapy: a longitudinal study with TST and ELISPOT. Pediatr Rheumatol. 2013 Dec 5;11(S2):P67.

340.	TAKEDA N, NOJIMA T, TERAO C, YUKAWA N, KAWABATA D, KOICHIROOMURA K, et al. Interferon-c release assay for diagnosing Mycobacterium tuberculosisdiseases in patients with systemic lupus erythematosus. Int J Rheum Dis. 2010;13(Suppl. 1):0146.

341.	Tam L-S, Leung C-C, Ying SK, Lee GK, Yim C-W, Leung Y-Y, et al. Risk of tuberculosis in patients with rheumatoid arthritis in Hong Kong--the role of TNF blockers in an area of high tuberculosis burden. Clin Exp Rheumatol. 2010;28(5):679–85.

342.	Tamborenea MN, Tate G, Mysler E, Debonis J, Schijedman A. Prevalence of positive ppd in a cohort of rheumatoid arthritis patients. Rheumatol Int [Internet]. 2010;30(5):613–6. Available from: https://www.embase.com/search/results?subaction=viewrecord&id=L50570842&from=export

343.	Tamura N, Kuwana M, Atsumi T, Takei S, Harigai M, Fujii T, et al. Infection events in japanese patients with rheumatoid arthritis treated with tofacitinib: Interim all-case post-marketing surveillance. Arthritis Rheumatol [Internet]. 2018;70((Tamura N.) Juntendo University, Tokyo, Japan(Kuwana M.) Nippon Medical School, Tokyo, Japan(Atsumi T.) Hokkaido University, Sapporo, Japan(Takei S.) Medical Center for Children, Kagoshima, Japan(Harigai M.) Tokyo Women’s Medical University, Tokyo, Japan():1672–4. Available from: https://www.embase.com/search/results?subaction=viewrecord&id=L626435950&from=export

344.	Tan Y, CH’NG S, ZAIN M, BAHARUDDIN H, AHMAD A, LAU I, et al. Quantiferon TB Gold as a screening tool for tuberculosis in rheumatology patients at a tertiary referral hospital for rheumatologic diseases. Int J Rheum Dis. 2016;19(Suppl. 2):APL16-0787.

345.	Tang I, So H, Luk L, Wong V, Pang S, Lao V, et al. Comparison of single and dual latent tuberculosis screening strategies before biologic and targeted therapy in patients with rheumatic diseases: a retrospective cohort study. Hong Kong Med J. 2020 Apr 2;

346.	Taxonera C, Ponferrada Á, Riestra S, Bermejo F, Saro C, Martín-Arranz MD, et al. Serial Tuberculin Skin Tests Improve the Detection of Latent Tuberculosis Infection in Patients With Inflammatory Bowel Disease. J Crohn’s Colitis. 2018 Nov 15;12(11):1270–9.

347.	Taylor P, Genovese M, Keystone E, Schlichting D, Beattie S, Macias W. Baricitinib, an oral janus kinase inhibitor, in the treatment of rheumatoid arthritis: Safety and efficacy in an open-label, long-term extension study1. Ann Rheum Dis [Internet]. 2014;73. Available from: https://www.embase.com/search/results?subaction=viewrecord&id=L71324959&from=export

348.	Thaci D, Williams D, Kerdel F, Wu J, Bereswill M, Sundaram M. Fourth-year interim safety and patient outcomes from ESPRIT, a 10-year postmarketing surveillance registry of adalimumab for moderate to severe psoriasis. J Am Acad Dermatol [Internet]. 2014;70(5):AB170. Available from: https://www.embase.com/search/results?subaction=viewrecord&id=L71390766&from=export

349.	Thi AA, Abbara A, Bouri S, Collin SM, Wolfson P, Owen L, et al. Challenges in screening for latent tuberculosis in inflammatory bowel disease prior to biologic treatment: A UK cohort study. Frontline Gastroenterol [Internet]. 2018;9(3):234–40. Available from: https://www.embase.com/search/results?subaction=viewrecord&id=L624483611&from=export

350.	Thomas K, Hadziyannis E, Hatzara C, Makris A, Tsalapaki C, Lazarini A, et al. Conversion and Reversion Rates of Tuberculosis Screening Assays in Patients With Rheumatic Diseases and Negative Baseline Screening Under Long-Term Biologic Treatment. Pathog Immun. 2020 Feb 26;5(1):34.

351.	Thomas K, Makris A, Tsalapaki C, Lazarini A, Klavdianou K, Antonatou K, et al. Frequent conversion and reverse conversion of tuberculin skin test but not of an interferon gamma release assay (t-spot. tb) during long term biologic treatment of rheumatic patients. Ann Rheum Dis [Internet]. 2017;76((Thomas K.; Makris A.; Tsalapaki C.; Lazarini A.; Klavdianou K.; Antonatou K.; Koutsianas C.; Hatzara C.; Hadziyannis E.; Vassilopoulos D.) Joint Rheumatology Program, Clinical Immunology-Rheumatology Unit, 2nd Department of Medicine and Laboratory, Natio):1376. Available from: https://www.embase.com/search/results?subaction=viewrecord&id=L621424118&from=export

352.	Tomio J, Yamana H, Matsui H, Yamashita H, Yoshiyama T, Yasunaga H. Tuberculosis screening prior to anti-tumor necrosis factor therapy among patients with immune-mediated inflammatory diseases in Japan: a longitudinal study using a large-scale health insurance claims database. Int J Rheum Dis [Internet]. 2017;20(11):1674–83. Available from: https://www.embase.com/search/results?subaction=viewrecord&id=L620194124&from=export

353.	Tsai T-F, Ho V, Song M, Szapary P, Kato T, Wasfi Y, et al. The safety of ustekinumab treatment in patients with moderate-to-severe psoriasis and latent tuberculosis infection. Br J Dermatol [Internet]. 2012;167(5):1145–52. Available from: https://www.embase.com/search/results?subaction=viewrecord&id=L365948131&from=export

354.	Tubach F, Salmon D, Ravaud P, Allanore Y, Goupille P, Bréban M, et al. Risk of tuberculosis is higher with anti-tumor necrosis factor monoclonal antibody therapy than with soluble tumor necrosis factor receptor therapy: The three-year prospective French research axed on tolerance of biotherapies registry. Arthritis Rheum [Internet]. 2009;60(7):1884–94. Available from: https://www.embase.com/search/results?subaction=viewrecord&id=L354872542&from=export

355.	Turner D, Koletzko S, Winter H, Baldassano RN, Dubinsky M, Faubion WA, et al. Safety of adalimumab in children and adolescents with moderate to severe Crohn’s disease: Interim results of the CAPE registry. J Pediatr Gastroenterol Nutr [Internet]. 2018;66((Turner D.) Institute of Pediatric Gastroenterology, Shaare Zedek Medical Center, Jerusalem, Israel(Koletzko S.) Dr. von Hauner Children’s Hospital, University Medical Center, Munich, Germany(Winter H.) Department of Pediatrics, Boston, United States(Bald):81–3. Available from: https://www.embase.com/search/results?subaction=viewrecord&id=L622343641&from=export

356.	Ukatu N, Hannaway M, Sink J, Rupley K, Gottlieb A. High incidence of latent tuberculosis infection in indigent patients receiving biologics in New York City. J Am Acad Dermatol [Internet]. 2019;81(4):AB220-. Available from: https://www.embase.com/search/results?subaction=viewrecord&id=L2002642761&from=export

357.	Uslu S, Can G, Cefle A, Yilmaz S, Kocaer SB, Inel TY, et al. The efficacy and safety of anti-TNFα treatment in ankylosing spondylitis patients with late onset compared to those with adult onset; The Data from TURKBIO Registry. Arthritis Rheumatol [Internet]. 2019;71:2707–9. Available from: https://www.embase.com/search/results?subaction=viewrecord&id=L633058156&from=export

358.	Vadan R, Cojocaru ML, Gheorghe C, Diculescu M, Stanel I, Zamfirescu N, et al. Tuberculosis in inflammatory bowel disease patients under treatment with anti TNF alfa agents: A frequent complication despite rigorous screening. United Eur Gastroenterol J [Internet]. 2016;4(5):A454-. Available from: https://www.embase.com/search/results?subaction=viewrecord&id=L619934791&from=export

359.	Vaidya B, Nakarmi S. Simultaneous Adalimumab and Antitubercular Treatment for Latent Tubercular Infection: An Experience from Nepal. Int J Rheumatol [Internet]. 2019;2019((Vaidya B., drbinitvaidya@yahoo.com; Nakarmi S., sweedie.shweta@gmail.com) Department of Rheumatology, National Center for Rheumatic Diseases (NCRD), Nepal). Available from: https://www.embase.com/search/results?subaction=viewrecord&id=L627661221&from=export

360.	Vajravelu RK, Osterman MT, Aberra FN, Roy JA, Lichtenstein GR, Mamtani R, et al. Indeterminate QuantiFERON-TB Gold Increases Likelihood of Inflammatory Bowel Disease Treatment Delay and Hospitalization. Inflamm Bowel Dis. 2018 Jan 1;24(1):217–26.

361.	Vajravelu RK, Osterman MT, Aberra F, Lichtenstein GR, Lewis J, Scott FI. Predictors of indeterminate quantiferon-TB gold testing in inflammatory bowel disease and impact on clinical outcomes. Gastroenterology [Internet]. 2015;148(4):S253. Available from: https://www.embase.com/search/results?subaction=viewrecord&id=L71885195&from=export

362.	Valido A, Dinis JS, Saavedra MJ, Fonseca JE. Tuberculosis under anti-TNF therapy: case series of a center (reporting the experience from the period 2006-2019). Acta Reum Port. 2020;45(4):281–7.

363.	van Vollenhoven RF, Hahn BH, Tsokos GC, Lipsky P, Fei K, Gordon RM, et al. Maintenance of Efficacy and Safety of Ustekinumab Through One Year in a Phase II Multicenter, Prospective, Randomized, Double-Blind, Placebo-Controlled Crossover Trial of Patients With Active Systemic Lupus Erythematosus. Arthritis Rheumatol [Internet]. 2020;72(5):761–8. Available from: https://www.embase.com/search/results?subaction=viewrecord&id=L2004533868&from=export

364.	Vassilopoulos D, Tsikrika S, Hatzara C, Podia V, Kandili A, Stamoulis N, et al. Comparison of Two Gamma Interferon Release Assays and Tuberculin Skin Testing for Tuberculosis Screening in a Cohort of Patients with Rheumatic Diseases Starting Anti-Tumor Necrosis Factor Therapy. Clin Vaccine Immunol. 2011 Dec;18(12):2102–8.

365.	Vassilopoulos D, Stamoulis N, Hadziyannis E, Archimandritis AJ. Usefulness of enzyme-linked immunospot assay (Elispot) compared to tuberculin skin testing for latent tuberculosis screening in rheumatic patients scheduled for anti-tumor necrosis factor treatment. J Rheumatol [Internet]. 2008;35(7):1271–6. Available from: https://www.embase.com/search/results?subaction=viewrecord&id=L352000770&from=export

366.	Velasco-Arnaiz E, Soriano-Arandes A, Latorre I, Altet N, Domínguez J, Fortuny C, et al. Performance of Tuberculin Skin Tests and Interferon-γ Release Assays in Children Younger Than 5 Years. Pediatr Infect Dis J. 2018 Dec;37(12):1235–41.

367.	Vélez P, Jaller JJ, Otero Escalante WJ, García EG, Rojo R, Chapman D, et al. Tofacitinib, an oral Janus kinase inhibitor, in patients from Colombia with rheumatoid arthritis: Pooled efficacy and safety analyses of data from phase III studies. Rev Colomb Reumatol [Internet]. 2018;25(4):233–44. Available from: https://www.embase.com/search/results?subaction=viewrecord&id=L2001344108&from=export

368.	Vencovsky J, Klein M, Jarosova K, Forejtova S, Becvar R, Sedova L, et al. Performance of Quantiferon test and analysis of its individual components in patients with inflammatory rheumatic diseases treated with anti-TNF drugs. Int J Rheum Dis [Internet]. 2010;13((Vencovsky J.; Klein M.; Jarosova K.; Forejtova S.; Becvar R.; Sedova L.; Svobodova R.; Putova I.; Pavelka K.) Institute of Rheumatology, Praha, Czech Republic):85. Available from: https://www.embase.com/search/results?subaction=viewrecord&id=L70197874&from=export

369.	Vortia E, Uko VE, Yen-Lieberman B, Frawley J, Worley SE, Danziger-Isakov L, et al. Low Indeterminate Rates Associated with Use of the QuantiFERON-TB Gold In-Tube Test in Children with Inflammatory Bowel Disease on Long-term Infliximab. Inflamm Bowel Dis [Internet]. 2018;24(4):877–82. Available from: https://www.embase.com/search/results?subaction=viewrecord&id=L621433377&from=export

370.	Wang P-H, Lin C-H, Chang T-H, Wu C-S. Chest roentgenography is complementary to interferon-gamma release assay in latent tuberculosis infection screening of rheumatic patients. BMC Pulm Med. 2020 Dec 31;20(1):232.

371.	Wang X, Wong SH, Wang X-S, Tang W, Liu C-Q, Niamul G, et al. Risk of tuberculosis in patients with immune-mediated diseases on biological therapies: A population-based study in a tuberculosis endemic region. Rheumatol (United Kingdom) [Internet]. 2019;58(5):803–10. Available from: https://www.embase.com/search/results?subaction=viewrecord&id=L628244454&from=export

372.	Winthrop KL, Harigai M, Genovese MC, Lindsey S, Takeuchi T, Fleischmann R, et al. Infections in baricitinib clinical trials for patients with active rheumatoid arthritis. Ann Rheum Dis [Internet]. 2020;79(10):1290–7. Available from: https://www.embase.com/search/results?subaction=viewrecord&id=L632867039&from=export

373.	Winthrop KL, Park S-H, Gul A, Cardiel MH, Gomez-Reino JJ, Tanaka Y, et al. Tuberculosis and other opportunistic infections in tofacitinib-treated patients with rheumatoid arthritis. Ann Rheum Dis [Internet]. 2016;75(6):1133–8. Available from: https://www.embase.com/search/results?subaction=viewrecord&id=L606017682&from=export

374.	Winthrop K, Calabrese L, Van Den Bosch F, Yamaoka K, Selmi C, Song Y, et al. Characterization of Serious Infections with Upadacitinib in Patients with Rheumatoid Arthritis. Arthritis Rheumatol [Internet]. 2020;72(SUPPL 10):460–2. Available from: https://www.embase.com/search/results?subaction=viewrecord&id=L634235254&from=export

375.	Wolf WA, Cotton CC, Runge T, Eluri S, Weimer E, Schmitz J, et al. Immunosuppression increases the odds of an indeterminate tuberculosis screen. Gastroenterology [Internet]. 2015;148(4):S473. Available from: https://www.embase.com/search/results?subaction=viewrecord&id=L71885953&from=export

376.	Wong A, Stock DA. Bacille calmette-guerin vaccination in infancy may influence a tuberculin skin test performed in adolescence or adulthood. Am J Respir Crit Care Med [Internet]. 2015;191. Available from: https://www.embase.com/search/results?subaction=viewrecord&id=L72053341&from=export

377.	Wong SH, Zheng L, Ip M, Tang W, Choi J, Kee KM, et al. The effect immunosuppressive drugs on performance of interferon-gamma release assay for tuberculosis screening in inflammatory bowel disease. United Eur Gastroenterol J [Internet]. 2013;1(1):A214-. Available from: https://www.embase.com/search/results?subaction=viewrecord&id=L72263498&from=export

378.	Wu C-Y, Chiu H-Y, Tsai T-F. The seroconversion rate of QuantiFERON-TB Gold In-Tube test in psoriatic patients receiving secukinumab and ixekizumab, the anti-interleukin-17A monoclonal antibodies. PLoS One. 2019 Dec 27;14(12):e0225112.

379.	Wu R, Long W. Activation of latent tuberculosis infection in rheumatic disease patients: A risk analysis. Eur J Immunol [Internet]. 2019;49((Wu R.) First Affiliated Hospital, Nanchang University, Department of Immunology and Rheumatology, NanChang, China(Long W.) First Affiliated Hospital, Nanchang University, NanChang, China):895–6. Available from: https://www.embase.com/search/results?subaction=viewrecord&id=L631548478&from=export

380.	Wu R. Efficacy and safety of prophylactic treatment on activiation of latent tuberculosis during glucocorticoid therapy in patients with rheumatic diseases. In: Poster Presentations. BMJ Publishing Group Ltd and European League Against Rheumatism; 2017. p. 1042.3-1043.

381.	Xiao X, Da G, Xie X, Liu X, Zhang L, Zhou B, et al. Tuberculosis in patients with systemic lupus erythematosus–a 37‐year longitudinal survey‐based study. J Intern Med. 2021 Jul 19;290(1):101–15.

382.	Yamamoto S, Nagatani K, Sato T, Iwamoto M, Takatori S, Minota S. Unaffected reaction level in tuberculin skin test by long-term therapy with tumor necrosis factor inhibitors for rheumatoid arthritis. Int J Rheum Dis [Internet]. 2017;20(5):584–8. Available from: https://www.embase.com/search/results?subaction=viewrecord&id=L616852170&from=export

383.	Yamamoto S, Takatori S, Iwamoto M, Nagatani K, Ikenoya K, Minota S. No change in the levels of ppd reaction during treatment with tnf inhibitors in rheumatoid arthritis patients in Japan, a country with high morbidity of tuberculosis. Arthritis Rheum [Internet]. 2011;63(10). Available from: https://www.embase.com/search/results?subaction=viewrecord&id=L70786243&from=export

384.	Yang QY, Liu YJ, Xu Y, Zhang L, Wang CD. Monitoring frequency of interferon gamma release assay for tuberculosis surveillance following infliximab therapy in patients with Crohn’s disease. J Dig Dis [Internet]. 2021;22(8):473–80. Available from: https://www.embase.com/search/results?subaction=viewrecord&id=L2013354051&from=export

385.	Ye L, Liu J, Lin Z, Cao Q. Does infliximab therapy increase incidence of tuberculosis in patients with inflammatory bowel disease in an endemic area: A nationwide study from China. J Crohn’s Colitis [Internet]. 2017;11((Ye L.; Liu J.; Lin Z.; Cao Q.) Inflammatory Bowel Disease Center, Sir Run Run Shaw Hospital, Zhejiang University, Hangzhou, China):S369–70. Available from: https://www.embase.com/search/results?subaction=viewrecord&id=L621116327&from=export

386.	Yeo Y, Jo K-W, Lee S-D, Kim WS, Kim DS, Shim TS. Initiation of anti-TNF therapy within 3 weeks of LTBI treatment in patients with immune-mediated inflammatory diseases. Eur Respir J [Internet]. 2014;44. Available from: https://www.embase.com/search/results?subaction=viewrecord&id=L71851887&from=export

387.	Yilmaz N, Aydin S, Inanc N, Direskeneli H, Yavuz S. Comparison of quantiferon-tb gold test and tuberculin skin test for identification of latent mycobacterium tuberculosis infection in lupus patients. Arthritis Rheum [Internet]. 2009;60:286. Available from: https://www.embase.com/search/results?subaction=viewrecord&id=L70373290&from=export

388.	Yonekura CL, Oliveira RDR, Titton DC, Ranza R, Ranzolin A, Hayata AL, et al. Incidence of tuberculosis among patients with rheumatoid arthritis using TNF blockers in Brazil: data from the Brazilian Registry of Biological Therapies in Rheumatic Diseases (Registro Brasileiro de Monitoração de Terapias Biológicas - BiobadaBrasil). Rev Bras Reum Engl Ed. 2017;57 Suppl 2:477–83.

389.	Yoo H-G, Yu HM, Jun JB, Jeon H-S, Yoo W-H. Risk factors of severe infections in patients with rheumatoid arthritis treated with leflunomide. Mod Rheumatol [Internet]. 2013;23(4):709–15. Available from: https://www.embase.com/search/results?subaction=viewrecord&id=L52112766&from=export

390.	Yoo IK, Choung RS, Hyun JJ, Kim SY, Jung SW, Koo JS, et al. Incidences of serious infections and tuberculosis among patients receiving anti-tumor necrosis factor-α therapy. Yonsei Med J [Internet]. 2014;55(2):442–8. Available from: https://www.embase.com/search/results?subaction=viewrecord&id=L372427055&from=export

391.	Yoo J-W, Jo K-W, Kang B-H, Kim MY, Yoo B, Lee C-K, et al. Mycobacterial diseases developed during anti-tumour necrosis factor - A therapy. Eur Respir J [Internet]. 2014;44(5):1289–95. Available from: https://www.embase.com/search/results?subaction=viewrecord&id=L612034736&from=export

392.	Zanova E, Kozakova D, Rybar I, Solovic I, Rovensky J. Four years of experience with quantiferon-TB gold test in patients with biological treatment. Ann Rheum Dis [Internet]. 2015;74((Zanova E.; Kozakova D.; Rybar I.; Solovic I.; Rovensky J.) National Institute of Rheumatic Diseases, Piestany, Slovakia):1032–3. Available from: https://www.embase.com/search/results?subaction=viewrecord&id=L72153824&from=export

393.	Zerôncio M, Blake A, Rana-Khan Q, Palo W, Bhayat F. Tuberculosis in patients treated with vedolizumab: Clinical trial and post-marketing case series. J Crohn’s Colitis [Internet]. 2017;11((Zerôncio M.) Clínica de Oncologia de Natal, Dept. of Gastroenterology, Natal, Brazil(Blake A.) Takeda Development Center Europe Ltd, Global Pharmacovig-ilance, London, United Kingdom(Rana-Khan Q.) Takeda Pharmaceutical International AG Singapore, Emergin):S410–1. Available from: https://www.embase.com/search/results?subaction=viewrecord&id=L621115782&from=export

394.	Zingaropoli MA, Pasculli P, Iannetta M, Perri V, Tartaglia M, Crisafulli SG, et al. Infectious risk in multiple sclerosis patients treated with disease-modifying therapies: A three-year observational cohort study. Mult Scler J - Exp Transl Clin. 2022 Jan 4;8(1):205521732110657.
