# Supplementary material for: Brazilian recommendations for the management of tuberculosis infection in immune-mediated inflammatory diseases
Source: Adv Rheumatol. 2025 Mar 20;65:18. doi: 10.1186/s42358-025-00449-4 (PMC13488801; doi:10.1186/s42358-025-00449-4)
Supplement: Supplementary file 2 [file 42358_2025_449_MOESM2_ESM.docx]

# **Brazilian recommendations for the management of tuberculosis infection in immune-mediated inflammatory diseases - Supplementary material**

**Appendix 1**

**Team members**

**Core Leadership Team (CLT)**

| **NAME** | **PROFICIENCY** |
| --- | --- |
| Viviane Angelina de Souza (VAS) | Rheumatologist |
| Gecilmara Cristina Salviato Pileggi (GCSP) | Rheumatologist |
| Virginia Fernandes Moça Trevisani (VFMT) | Rheumatologist |
| Ana Luiza Mendes Amorim Caparroz (ALMAC) | Pediatric Rheumatologist |
| Anna Carolina Faria Moreira Gomes Tavares (ACFMGT) | Pediatric Rheumatologist |
| Ana Karla Guedes de Melo (AKGM) | Rheumatologist |
| Odirlei Andre Monticielo (OAM) | Rheumatologist |
| Ricardo Machado Xavier (RMX) | Rheumatologist |
| Marcelo de Medeiros Pinheiro (MMP) | Rheumatologist |
| Ana Cristina de Medeiros-Ribeiro (ACMR) | Rheumatologist |
| Maria Fernanda Brandao de Resende Guimaraes (MFBRG) | Rheumatologist |
| Flavio Sztajnbok (FS) | Pediatric Rheumatologist |
| Liliana Andrade Chebli (LAC) | Gastroenterologist |
| Anete Trajman (AT) | Internist |
| Sidney Bombarda (SB) | Pulmonologist |

**Literature Review Team (LRT)**

| **NAME** | **PROFICIENCY** |
| --- | --- |
| Adriana Maria Kakehasi (AMK) | Rheumatologist |
| Ana Cristina de Medeiros-Ribeiro (ACMR) | Rheumatologist |
| Ana Karla Guedes de Melo (AKGM) | Rheumatologist |
| Ana Luiza Mendes Amorim Caparroz (ALMAC) | Pediatric Rheumatologist |
| Anna Carolina Faria Moreira Gomes Tavares (ACFMGT) | Pediatric Rheumatologist |
| Blanca Elena Rios Gomes Bica (BERGB) | Pediatric Rheumatologist |
| Claudia Diniz Lopes Marques (CDLM) | Rheumatologist |
| Flavio Sztajnbok (FS) | Pediatric Rheumatologist |
| Gecilmara Cristina Salviato Pileggi (GCSP) | Pediatric Rheumatologist |
| Joana Starling de Carvalho (JSC) | Rheumatologist |
| Ketty Lysie Libardi Lira Machado (KLLLM) | Rheumatologist |
| Liliana Andrade Chebli (LAC) | Gastroenterologist |
| Lilian David de Azevedo Valadares (LDAV) | Rheumatologist |
| Marcelo de Medeiros Pinheiro (MMP) | Rheumatologist |
| Maria Cecilia de Carvalho Bortoletto (MCCB) | Dermatologist |
| Maria Fernanda Brandão de Resende Guimarães (MFBRG) | Rheumatologist |
| Rejane Maria Rodrigues de Abreu Vieira (RMRAV) | Rheumatologist |
| Ricardo Machado Xavier (RMX) | Rheumatologist |
| Sidney Bombarda (SB) | Pulmonologist |
| Odirlei Andre Monticielo (OAM) | Rheumatologist |
| Valéria Maria Augusto (VMA) | Pulmonologist |
| Vitor Alves Cruz (VAC) | Rheumatologist |
| Viviane Angelina de Souza (VAS) | Rheumatologist |

**Voting Panel**

| **NAME** | **PROFICIENCY** |
| --- | --- |
| Adriana Maria Kakehasi (AMK) | Rheumatologist |
| Ana Cristina de Medeiros-Ribeiro (ACMR) | Rheumatologist |
| Ana Karla Guedes de Melo (AKGM) | Rheumatologist |
| Ana Luiza Bierrenbach (ALB) | Epidemiologist |
| Ana Luiza Mendes Amorim Caparroz (ALMAC) | Pediatric Rheumatologist |
| Anna Carolina Faria Moreira Gomes Tavares (ACFMGT) | Pediatric Rheumatologist |
| Ana Paula Monteiro Gomides Reis (APMGR) | Rheumatologist |
| Anete Trajman (AT) | Internist |
| Blanca Elena Rios Gomes Bica (BERGB) | Pediatric Rheumatologist |
| Claudia Diniz Lopes Marques (CDLM) | Rheumatologist |
| Cristina Flores (CF) | Gastroenterologist |
| Denise Silva Rodrigues (DSR) | Infectious diseases specialist |
| Eduardo dos Santos Paiva (ESP) | Rheumatologist |
| Eliana Dias Matos (EDM) | Pulmonologist |
| Fernanda Dockhorn Costa Johansen (FDCJ) | Ministry of Health |
| Flavio Sztajnbok (FS) | Pediatric Rheumatologist |
| Gecilmara Cristina Salviato Pileggi (GCSP) | Pediatric Rheumatologist |
| Helio Arthur Bacha (HAB) | Infectious diseases specialist |
| Joana Starling de Carvalho (JSC) | Rheumatologist |
| José Roberto Provenza (JRP) | Rheumatologist |
| Ketty Lysie Libardi Lira Machado (KLLLM) | Rheumatologist |
| Licia Maria Henrique da Mota (LMHM) | Rheumatologist |
| Liliana Andrade Chebli (LAC) | Gastroenterologist |
| Lilian David de Azevedo Valadares (LDAV) | Rheumatologist |
| Marcelo de Medeiros Pinheiro (MMP) | Rheumatologist |
| Marco Antônio Araújo da Rocha Loures (MAARL) | Rheumatologist |
| Margareth Maria Pretti Dalcolmo (MMPD) | Pulmonologist |
| Maria Cecilia de Carvalho Bortoletto (MCCB) | Dermatologist |
| Maria Fernanda Brandão de Resende Guimarães (MFBRG) | Rheumatologist |
| Max Igor Banks Ferreira Lopes (MIBFL) | Infectious diseases specialist |
| Rejane Maria Rodrigues de Abreu Vieira (RMRAV) | Rheumatologist |
| Ricardo Machado Xavier (RMX) | Rheumatologist |
| Ricardo Romiti (RR) | Dermatologist |
| Rogerio Saad-Hossne (RSH) | Gastroenterologist |
| Rozana Mesquita Ciconelli (RMC) | Rheumatologist |
| Sidney Bombarda (SB) | Pulmonologist |
| Odirlei Andre Monticielo (OAM) | Rheumatologist |
| Valderilio Feijó Azevedo (VFA) | Rheumatologist |
| Valéria Maria Augusto (VMA) | Pulmonologist |
| Virginia Fernandes Moça Trevisani (VFMT) | Rheumatologist |
| Vitor Alves Cruz (VAC) | Rheumatologist |
| Viviane Angelina de Souza (VAS) | Rheumatologist |

**Search strategies and flowcharts of the study selection processes for each clinical question**

**CLINICAL QUESTION 1**

Search strategy

*(Tuberculosis OR Tuberculoses OR Koch's Disease OR Koch Disease OR Kochs Disease OR Tuberculous OR Tuberculoma OR Tb) AND ((Steroids OR Prednisolone OR Methylprednisolone OR Glucocorticoids OR Dexamethasone OR Pregnadienes OR Prednisone OR Pregnadienediols OR Pregnadienetriols) OR (Immunosuppressive Agents OR Antirheumatic Agents OR Azathioprine OR Cyclophosphamide OR Antirheumatic Agents OR Cyclosporine OR Cyclosporin OR Tacrolimus OR Mycophenolic Acid OR Mycophenolate) OR (Methotrexate OR Immunosuppressive Agents OR Antirheumatic Agents OR Leflunomide OR Sulfasalazine OR Sulfasalazin OR Sulphasalazine OR Chloroquine OR Chlorochin OR Hydroxychloroquine) OR (Anti-CD20 OR Veltuzumab OR Obinutuzumab OR Antibodies, Monoclonal, Humanized OR Ocrelizumab OR Ofatumumab OR Antibodies, Monoclonal OR Rituximab OR Belimumab OR Rilonacept OR Interleukin-1 OR IL-1 OR Canakinumab OR Interleukin-6 OR IL-6 OR Tocilizumab OR Anti-TNF-alpha OR Anti TNF-alpha OR Etanercept OR Infliximab OR Adalimumab OR Golimumab OR Certolizumab OR Abatacept OR CTLA-4 OR Secukinumab OR Interleukin-17 OR IL-17 OR Ixekizumab OR Brodalumab OR Ustekinumab OR Interleukin-12 OR IL-12 OR Interleukin-23 OR IL-23 OR Risankizumab OR Guselkumab) OR (JAK inhibitor OR JAK inhibitors OR Janus Kinase Inhibitors OR Upadacitinib OR Antirheumatic Agents OR Protein Kinase Inhibitors OR Tofacitinib OR Baricitinib) OR (Vedolizumab OR Antibodies, Monoclonal, Humanized OR Integrins OR Integrin)) AND (risk or risks OR probability OR risk factor OR risk factors OR risk assessment OR prevalence OR prevalences OR epidemiology OR epidemiologies OR epidemiologic OR incidence OR rate OR data collection OR vital statistics OR metadata OR records OR data)*

Flowchart of the study selection process

**CLINICAL QUESTION 2**

Search strategy

*(Tuberculosis OR Tuberculoses OR Koch's Disease OR Koch Disease OR Kochs Disease OR Tuberculous OR Tuberculoma OR Tb) AND ((Steroids OR Prednisolone OR Methylprednisolone OR Glucocorticoids OR Dexamethasone OR Pregnadienes OR Prednisone OR Pregnadienediols OR Pregnadienetriols) OR (Immunosuppressive Agents OR Antirheumatic Agents OR Azathioprine OR Cyclophosphamide OR Antirheumatic Agents OR Cyclosporine OR Cyclosporin OR Tacrolimus OR Mycophenolic Acid OR Mycophenolate) OR (Methotrexate OR Immunosuppressive Agents OR Antirheumatic Agents OR Leflunomide OR Sulfasalazine OR Sulfasalazin OR Sulphasalazine OR Chloroquine OR Chlorochin OR Hydroxychloroquine) OR (Anti-CD20 OR Veltuzumab OR Obinutuzumab OR Antibodies, Monoclonal, Humanized OR Ocrelizumab OR Ofatumumab OR Antibodies, Monoclonal OR Rituximab OR Belimumab OR Rilonacept OR Interleukin-1 OR IL-1 OR Canakinumab OR Interleukin-6 OR IL-6 OR Tocilizumab OR Anti-TNF-alpha OR Anti TNF-alpha OR Etanercept OR Infliximab OR Adalimumab OR Golimumab OR Certolizumab OR Abatacept OR CTLA-4 OR Secukinumab OR Interleukin-17 OR IL-17 OR Ixekizumab OR Brodalumab OR Ustekinumab OR Interleukin-12 OR IL-12 OR Interleukin-23 OR IL-23 OR Risankizumab OR Guselkumab) OR (JAK inhibitor OR JAK inhibitors OR Janus Kinase Inhibitors OR Upadacitinib OR Antirheumatic Agents OR Protein Kinase Inhibitors OR Tofacitinib OR Baricitinib) OR (Vedolizumab OR Antibodies, Monoclonal, Humanized OR Integrins OR Integrin)) AND (risk or risks OR probability OR risk factor OR risk factors OR risk assessment OR prevalence OR prevalences OR epidemiology OR epidemiologies OR epidemiologic OR incidence OR rate OR data collection OR vital statistics OR metadata OR records OR data)*

Flowchart of the study selection process

**CLINICAL QUESTION 3**

Search strategy

*((Tuberculosis OR Tuberculoses OR Latent Tuberculosis OR Koch's Disease OR Koch Disease OR Kochs Disease OR Tuberculous OR Tuberculoma OR (Laten* AND (Tb* OR Tubercul*)) OR Ltb* OR Tubercul*) AND ((Steroids OR Prednisolone OR Methylprednisolone OR Glucocorticoids OR Dexamethasone OR Pregnadienes OR Prednisone OR Pregnadienediols OR Pregnadienetriols) OR (Immunosuppressive Agents OR Antirheumatic Agents OR Azathioprine OR Cyclophosphamide OR Antirheumatic Agents OR Cyclosporine OR Cyclosporin OR Tacrolimus OR Mycophenolic Acid OR Mycophenolate) OR (Methotrexate OR Immunosuppressive Agents OR Antirheumatic Agents OR Leflunomide OR Sulfasalazine OR Sulfasalazin OR Sulphasalazine OR Chloroquine OR Chlorochin OR Hydroxychloroquine) OR (Anti-CD20 OR Veltuzumab OR Obinutuzumab OR Antibodies, Monoclonal, Humanized OR Ocrelizumab OR Ofatumumab OR Antibodies, Monoclonal OR Rituximab OR Belimumab OR Rilonacept OR Interleukin-1 OR IL-1 OR Canakinumab OR Interleukin-6 OR IL-6 OR Tocilizumab OR Anti-TNF-alpha OR Anti TNF-alpha OR Etanercept OR Infliximab OR Adalimumab OR Golimumab OR Certolizumab OR Abatacept OR CTLA-4 OR Secukinumab OR Interleukin-17 OR IL-17 OR Ixekizumab OR Brodalumab OR Ustekinumab OR Interleukin-12 OR IL-12 OR Interleukin-23 OR IL-23 OR Risankizumab OR Guselkumab) OR (JAK inhibitor OR JAK inhibitors OR Janus Kinase Inhibitors OR Upadacitinib OR Antirheumatic Agents OR Protein Kinase Inhibitors OR Tofacitinib OR Baricitinib) OR (Vedolizumab OR Antibodies, Monoclonal, Humanized OR Integrins OR Integrin)) AND ((Antitubercular Agents OR Isoniazid OR Rifampin OR Rifampicin OR Antibiotics, Antitubercular OR Rifapentine OR Quinolones OR Fluoroquinolones OR Ciprofloxacin OR Ofloxacin OR Nalidixic Acid OR Quinolinones OR Ketoquinolines OR Oxoquinolines) OR (Reactivation OR recurrence OR relapse)))*

Flowchart of the study selection process

**CLINICAL QUESTION 4**

Search strategy

*(((LTBI OR Latent Tuberculosis OR (Laten* AND (Tb* OR Tubercul*)) OR Ltb*)) AND (quantiferon* OR interferon* OR IGRA* OR ELISPOT OR t spot*) AND (Diagnosis/broad[filter] OR Therapy/broad[filter] OR Prognosis/broad[filter] OR comparative study OR epidemiologic methods OR evaluation study)*

Flowchart of the study selection process

**CLINICAL QUESTION 5**

Search strategy

*((quantiferon* OR QFT* OR t spot* OR Enzyme-Linked Immunosorbent Assay OR Interferon-gamma Release Tests) OR ((interferon* OR IFN*) AND gamma AND (release* OR test OR assay*)) OR ((y-interferon OR interferon-y) AND (release* OR test OR assay*)) OR IGRA*) AND (((laten* AND (tb OR tubercul*)) OR Latent Tuberculosis OR ltb* OR tubercul*)) AND (TUBERCULIN* OR PPD)*

Flowchart of the study selection process

**CLINICAL QUESTION 6**

Search strategy

*((((quantiferon* OR QFT* OR t spot* OR Enzyme-Linked Immunosorbent Assay OR Interferon-gamma Release Tests) OR ((interferon* OR IFN*) AND gamma AND (release* OR test OR assay*)) OR ((y-interferon OR interferon-y) AND (release* OR test OR assay*)) OR IGRA*) AND (((laten* AND (tb OR tubercul*)) OR Latent Tuberculosis OR ltb* OR tubercul*))) AND (((rheumatoid AND arthritis) OR (sjogren AND syndrome) OR (juvenile AND rheumatoid AND arthritis) OR (psoriatic AND arthritis) OR spondylarthritis OR (systemic AND sclerosis) OR psoriasis OR dermatomyositis OR myositis OR (systemic AND lupus AND erythematosus) OR vasculitis OR (inflammatory AND bowel AND disease) OR (crohn AND disease) OR (ulcerative AND colitis)))) AND ((((sensitiv*[Title/Abstract]) OR (sensitivity and specificity [MeSH Terms]) OR (specificity [Title/Abstract]))))*

Flowchart of the study selection process

**CLINICAL QUESTION 7**

Search strategy

*(((((((((rheumatoid AND arthritis) OR (sjogren AND syndrome) OR (juvenile AND rheumatoid AND arthritis) OR (psoriatic AND arthritis) OR spondylarthritis OR (systemic AND sclerosis) OR psoriasis OR dermatomyositis OR myositis OR (systemic AND lupus AND erythematosus) OR vasculitis OR (inflammatory AND bowel AND disease) OR (crohn AND disease) OR (ulcerative AND colitis))))) OR (((Anti-CD20 OR Veltuzumab OR Obinutuzumab OR Antibodies, Monoclonal, Humanized OR Ocrelizumab OR Ofatumumab OR Antibodies, Monoclonal OR Rituximab OR Belimumab OR Rilonacept OR Interleukin-1 OR IL-1 OR Canakinumab OR Interleukin-6 OR IL-6 OR Tocilizumab OR Anti-TNF-alpha OR tumor necrosis factor inhibitors OR Anti TNF-alpha OR Etanercept OR Infliximab OR Adalimumab OR Golimumab OR Certolizumab OR Abatacept OR CTLA-4 OR Secukinumab OR Interleukin-17 OR IL-17 OR Ixekizumab OR Brodalumab OR Ustekinumab OR Interleukin-12 OR IL-12 OR Interleukin-23 OR IL-23 OR Risankizumab OR Guselkumab OR bDMARDs))))) AND ((((((quantiferon* OR QFT* OR t spot* OR Enzyme-Linked Immunosorbent Assay OR Interferon-gamma Release Tests) OR ((interferon* OR IFN*) AND gamma AND (release* OR test OR assay*)) OR ((y-interferon OR interferon-y) AND (release* OR test OR assay*)) OR IGRA*)))) AND (((((laten* AND (tb OR tubercul*)) OR Latent Tuberculosis OR ltb* OR tubercul*))))))) OR ((((((((quantiferon* OR QFT* OR t spot* OR Enzyme-Linked Immunosorbent Assay OR Interferon-gamma Release Tests) OR ((interferon* OR IFN*) AND gamma AND (release* OR test OR assay*)) OR ((y-interferon OR interferon-y) AND (release* OR test OR assay*)) OR IGRA*)))) AND ((((((rheumatoid AND arthritis) OR (sjogren AND syndrome) OR (juvenile AND rheumatoid AND arthritis) OR (psoriatic AND arthritis) OR spondylarthritis OR (systemic AND sclerosis) OR psoriasis OR dermatomyositis OR myositis OR (systemic AND lupus AND erythematosus) OR vasculitis OR (inflammatory AND bowel AND disease) OR (crohn AND disease) OR (ulcerative AND colitis))))) OR (((Anti-CD20 OR Veltuzumab OR Obinutuzumab OR Antibodies, Monoclonal, Humanized OR Ocrelizumab OR Ofatumumab OR Antibodies, Monoclonal OR Rituximab OR Belimumab OR Rilonacept OR Interleukin-1 OR IL-1 OR Canakinumab OR Interleukin-6 OR IL-6 OR Tocilizumab OR Anti-TNF-alpha OR tumor necrosis factor inhibitors OR Anti TNF-alpha OR Etanercept OR Infliximab OR Adalimumab OR Golimumab OR Certolizumab OR Abatacept OR CTLA-4 OR Secukinumab OR Interleukin-17 OR IL-17 OR Ixekizumab OR Brodalumab OR Ustekinumab OR Interleukin-12 OR IL-12 OR Interleukin-23 OR IL-23 OR Risankizumab OR Guselkumab OR bDMARDs)))))) AND (((Indeterminate OR diagnostic errors OR undetermined OR uncertain OR indefinite OR inaccurate OR imprecise OR undecided OR indecisive OR borderline))))*

Flowchart of the study selection process

**CLINICAL QUESTION 8**

Search strategy

*((Mantoux OR PPD OR Tuberculin OR TST) AND ("false negative" OR "false positive" OR "cut off" OR cut-off OR "5 mm" OR "5mm"))*

Flowchart of the study selection process

**CLINICAL QUESTION 9**

Search strategy

*(Tuberculosis OR Tuberculoses OR Latent Tuberculosis OR Koch's Disease OR Koch Disease OR Kochs Disease OR Tuberculous OR Tuberculoma OR (Laten* AND (Tb OR Tubercul*)) OR Ltb OR Tubercul*) AND ((quantiferon* OR QFT* OR t spot* OR Enzyme-Linked Immunosorbent Assay OR Interferon-gamma Release Tests) OR ((interferon* OR IFN*) AND gamma* AND (release* or test* or assay*)) OR ((y-interferon OR interferon-y) AND (release* OR test* OR assay*)) OR IGRA*) OR (Tuberculin* OR PPD OR Mantoux)) AND (Immunosuppressive Agents OR Antirheumatic Agents OR Azathioprine OR Cyclophosphamide OR Antirheumatic Agents OR Cyclosporine OR Cyclosporin OR Tacrolimus OR Mycophenolic Acid OR Mycophenolate OR Methotrexate OR Immunosuppressive Agents OR Antirheumatic Agents OR Leflunomide OR Sulfasalazine OR Sulfasalazin OR Sulphasalazine OR Chloroquine OR Chlorochin OR Hydroxychloroquine OR Anti-CD20 OR Veltuzumab OR Obinutuzumab OR Antibodies, Monoclonal, Humanized OR Ocrelizumab OR Ofatumumab OR Antibodies, Monoclonal OR Rituximab OR Belimumab OR Rilonacept OR Interleukin-1 OR IL-1 OR Canakinumab OR Interleukin-6 OR IL-6 OR Tocilizumab OR Anti-TNF-α OR Anti TNF-alpha OR Etanercept OR Infliximab OR Adalimumab OR Golimumab OR Certolizumab OR Abatacept OR CTLA-4 OR Secukinumab OR Interleukin-17 OR IL-17 OR Ixekizumab OR Brodalumab OR Ustekinumab OR Interleukin-12 OR IL-12 OR Interleukin-23 OR IL-23 OR Risankizumab OR Guselkumab OR JAK inhibitor OR JAK inhibitors OR Janus Kinase Inhibitors OR Upadacitinib OR Antirheumatic Agents OR Protein Kinase Inhibitors OR Tofacitinib OR Baricitinib OR Vedolizumab OR Antibodies, Monoclonal, Humanized OR Integrins OR Integrin OR Steroids OR Prednisolone OR Methylprednisolone OR Glucocorticoids OR Dexamethasone OR Pregnadienes OR Prednisone OR Pregnadienediols OR Pregnadienetriols)*

Flowchart of the study selection process

**CLINICAL QUESTION 10**

Search strategy

*((((((quantiferon* OR QFT* OR t spot* OR Enzyme-Linked Immunosorbent Assay OR Interferon-gamma Release Tests) OR ((interferon* OR IFN*) AND gamma* AND (release* or test* or assay*)) OR ((y-interferon OR interferon-y) AND (release* OR test* OR assay*)) OR IGRA*) OR (Tuberculin* OR PPD OR Mantoux))) AND (((Antitubercular Agents) OR (Isoniazid) OR (Rifampin) OR (Rifampicin) OR (Antibiotics, Antitubercular) OR (Rifapentine) OR (Quinolones) OR (Fluoroquinolones) OR (Ciprofloxacin) OR (Ofloxacin) OR (Nalidixic Acid) OR (Quinolinones) OR (Ketoquinolines) OR (Oxoquinolines)))) AND ((Latent Tuberculosis OR (Laten* AND (Tb OR Tubercul*)) OR Ltb))) AND ((follow-up OR follow up OR followup OR surveillance OR monitor OR monitoring or surveillance OR surveillances OR vigilance OR management))*

Flowchart of the study selection process

**CLINICAL QUESTION 11**

Search strategy

*((((quantiferon* OR QFT* OR "t spot" OR IGRA* OR Tuberculin* OR PPD)) AND (("Immunosuppressive Agents" OR "Antirheumatic Agents" OR Azathioprine OR Cyclophosphamide OR "Antirheumatic Agents" OR Cyclosporine OR Cyclosporin OR Tacrolimus OR "Mycophenolic Acid" OR Mycophenolate OR Methotrexate OR "Immunosuppressive Agents" OR "Antirheumatic Agents" OR Leflunomide OR Sulfasalazine OR Sulfasalazin OR Sulphasalazine OR Chloroquine OR Chlorochin OR Hydroxychloroquine OR Anti-CD20 OR Veltuzumab OR Obinutuzumab OR Antibodies, Monoclonal, Humanized OR Ocrelizumab OR Ofatumumab OR "Antibodies, Monoclonal" OR Rituximab OR Belimumab OR Rilonacept OR Interleukin-1 OR IL-1 OR Canakinumab OR Interleukin-6 OR IL-6 OR Tocilizumab OR Anti-TNF-α OR Anti TNF-alpha OR Etanercept OR Infliximab OR Adalimumab OR Golimumab OR Certolizumab OR Abatacept OR CTLA-4 OR Secukinumab OR Interleukin-17 OR IL-17 OR Ixekizumab OR Brodalumab OR Ustekinumab OR Interleukin-12 OR IL-12 OR Interleukin-23 OR IL-23 OR Risankizumab OR Guselkumab OR JAK inhibitor OR JAK inhibitors OR Janus Kinase Inhibitors OR Upadacitinib OR Protein Kinase Inhibitors OR Tofacitinib OR Baricitinib OR Vedolizumab OR "Antibodies, Monoclonal, Humanized" OR Integrins OR Integrin))) AND (((Tuberculosis OR Tuberculoses OR Latent Tuberculosis OR Koch's Disease OR Koch Disease OR Kochs Disease OR Tuberculous OR Tuberculoma OR (Laten* AND (Tb* OR Tubercul*)) OR Ltb* OR Tubercul*))) AND (Switch*)*

Flowchart of the study selection process

**CLINICAL QUESTION 12**

Search strategy

*((((Antitubercular Agents) OR (Isoniazid) OR (Rifampin) OR (Rifampicin) OR (Antibiotics, Antitubercular) OR (Rifapentine) OR (Quinolones) OR (Fluoroquinolones) OR (Ciprofloxacin) OR (Ofloxacin) OR (Nalidixic Acid) OR (Quinolinones) OR (Ketoquinolines) OR (Oxoquinolines))) AND (((Tuberculosis OR Tuberculoses OR Latent Tuberculosis OR Koch's Disease OR Koch Disease OR Kochs Disease OR Tuberculous OR Tuberculoma OR (Laten* AND (Tb* OR Tubercul*)) OR Ltb* OR Tubercul*)))) AND ((Steroids OR Prednisolone OR Methylprednisolone OR Glucocorticoids OR Dexamethasone OR Pregnadienes OR Prednisone OR Pregnadienediols OR Pregnadienetriol OR (Immunosuppressive Agents) OR (Antirheumatic Agents) OR Azathioprine OR Cyclophosphamide OR Cyclosporine OR Cyclosporin OR Tacrolimus OR Mycophenolic Acid OR Mycophenolate OR Methotrexate OR Leflunomide OR Sulfasalazine OR Sulfasalazin OR Sulphasalazine OR Chloroquine OR Chlorochin OR Hydroxychloroquine OR Anti-CD20 OR Veltuzumab OR Obinutuzumab OR Antibodies, Monoclonal, Humanized OR Ocrelizumab OR Ofatumumab OR Antibodies, Monoclonal OR Rituximab OR Belimumab OR Rilonacept OR Interleukin-1 OR IL-1 OR Canakinumab OR Interleukin-6 OR IL-6 OR Tocilizumab OR Anti-TNF-α OR Anti TNF-alpha OR Etanercept OR Infliximab OR Adalimumab OR Golimumab OR Certolizumab OR Abatacept OR CTLA-4 OR Secukinumab OR Interleukin-17 OR IL-17 OR Ixekizumab OR Brodalumab OR Ustekinumab OR Interleukin-12 OR IL-12 OR Interleukin-23 OR IL-23 OR Risankizumab OR Guselkumab OR JAK inhibitor OR JAK inhibitors OR Janus Kinase Inhibitors OR Upadacitinib OR Antirheumatic Agents OR Protein Kinase Inhibitors OR Tofacitinib OR Baricitinib OR Vedolizumab OR Integrins OR Integrin))*

Flowchart of the study selection process

**CLINICAL QUESTION 13**

Search strategy

((((Antitubercular Agents) OR (Isoniazid) OR (Rifampin) OR (Rifampicin) OR (Antibiotics, Antitubercular) OR (Rifapentine) OR (Quinolones) OR (Fluoroquinolones) OR (Ciprofloxacin) OR (Ofloxacin) OR (Nalidixic Acid) OR (Quinolinones) OR (Ketoquinolines) OR (Oxoquinolines))) AND (((Tuberculosis OR Tuberculoses OR Latent Tuberculosis OR Koch's Disease OR Koch Disease OR Kochs Disease OR Tuberculous OR Tuberculoma OR (Laten* AND (Tb* OR Tubercul*)) OR Ltb* OR Tubercul*)))) AND ((Steroids OR Prednisolone OR Methylprednisolone OR Glucocorticoids OR Dexamethasone OR Pregnadienes OR Prednisone OR Pregnadienediols OR Pregnadienetriol OR (Immunosuppressive Agents) OR (Antirheumatic Agents) OR Azathioprine OR Cyclophosphamide OR Cyclosporine OR Cyclosporin OR Tacrolimus OR Mycophenolic Acid OR Mycophenolate OR Methotrexate OR Leflunomide OR Sulfasalazine OR Sulfasalazin OR Sulphasalazine OR Chloroquine OR Chlorochin OR Hydroxychloroquine OR Anti-CD20 OR Veltuzumab OR Obinutuzumab OR Antibodies, Monoclonal, Humanized OR Ocrelizumab OR Ofatumumab OR Antibodies, Monoclonal OR Rituximab OR Belimumab OR Rilonacept OR Interleukin-1 OR IL-1 OR Canakinumab OR Interleukin-6 OR IL-6 OR Tocilizumab OR Anti-TNF-α OR Anti TNF-alpha OR Etanercept OR Infliximab OR Adalimumab OR Golimumab OR Certolizumab OR Abatacept OR CTLA-4 OR Secukinumab OR Interleukin-17 OR IL-17 OR Ixekizumab OR Brodalumab OR Ustekinumab OR Interleukin-12 OR IL-12 OR Interleukin-23 OR IL-23 OR Risankizumab OR Guselkumab OR JAK inhibitor OR JAK inhibitors OR Janus Kinase Inhibitors OR Upadacitinib OR Antirheumatic Agents OR Protein Kinase Inhibitors OR Tofacitinib OR Baricitinib OR Vedolizumab OR Integrins OR Integrin))

Flowchart of the study selection process

**CLINICAL QUESTION 14**

Search strategy

*((quantiferon* OR QFT* OR t spot* OR IGRA*) OR (Tuberculin* OR PPD)) AND ((BCG OR Bacillus Calmette Guerin OR Calmette OR Calmettes))*

Flowchart of the study selection process
